# Supplementary material for: Rate pressure product as a novel predictor of long-term adverse outcomes in patients after percutaneous coronary intervention: a retrospective cohort study
Source: BMJ Open. 2023 Apr 4;13(4):e067951. doi: 10.1136/bmjopen-2022-067951 (PMC10083747; doi:10.1136/bmjopen-2022-067951)
Supplement: Supplementary data [file bmjopen-2022-067951supp001.pdf]

SUPPLEMENTARY MATERIAL

Supplementary Table 1 Characteristics of participants of the two groups

Variables	ACS				Stable CAD			
	RPP<10 269 (n=1444)	RPP≥10 269 (n=585)	X^2or t	P value	RPP<10 269 (n=2574)	RPP≥10 269 (n=1412)	X^2or t	P value
Age, years	59.57±10.89	60.60±11.10	-1.930	0.054	56.56±10.89	60.63±10.40	-0.835	**<0.001**
Male, n (%)	358(24.8)	169(28.9)	3.634	0.057	603(23.4)	413(29.2)	16.277	**<0.001**
Smoking, n (%)	607(42.0)	228(39.0)	1.611	0.204	1079(41.9)	490(34.7)	19.895	**<0.001**
Alcohol drinking, n (%)	434(30.1)	167(28.5)	0.454	0.500	783(30.4)	377(26.7)	6.115	**0.013**
Diabetes, n (%)	301(20.8)	177(30.3)	20.478	**<0.001**	546(21.2)	423(30.0)	37.900	**<0.001**
Hypertension, n (%)	555(38.4)	309(52.8)	35.239	**<0.001**	923(35.9)	758(53.7)	118.784	**<0.001**
BUN, mmol/L	5.50±1.71	5.56±1.84	-0.695	0.487	5.48±1.61	5.60±1.67	-2.306	**0.021**
Cr, umol/L	76.00±20.77	77.59±21.79	-1.510	0.131	75.65±18.84	75.40±22.02	0.382	0.703
UA, mmol/L	320.62±89.64	328.53±92.28	-1.750	0.080	325.83±89.49	319.64±90.43	2.051	**0.040**
GLU, mmol/L	6.41±3.02	7.22±3.39	-5.209	**<0.001**	6.41±2.97	6.80±3.39	-3.706	**<0.001**
TG, mmol/L	1.88±1.25	2.04±1.39	-2.531	**0.011**	1.88±1.22	1.90±1.33	-0.470	0.638
TC, mmol/L	3.92±1.07	4.04±1.10	-2.089	**0.037**	3.95±1.11	4.00±1.14	-1.371	0.170
LDL-C, mmol/L	2.41±0.87	2.50±0.96	-1.908	0.057	2.45±0.91	2.51±0.95	-1.866	0.062
HDL-C, mmol/L	1.01±0.44	1.01±0.46	0.288	0.774	1.02±0.48	1.03±0.54	-0.361	0.718
CCB, n (%)	184(12.8)	67(11.5)	0.644	0.422	278(10.9)	160(11.4)	0.270	0.603
β-blocker, n (%)	580(40.4)	234(40.2)	0.004	0.948	1051(41.0)	551(39.2)	1.268	0.260
ACEI or ARB, n (%)	325(22.6)	142(24.4)	0.760	0.383	569(22.2)	330(23.5)	0.878	0.349
Statins, n (%)	797(55.8)	303(52.2)	2.123	0.145	1410(55.3)	732(52.2)	3.284	0.070
LVEF, %	61.09±6.88	60.84±7.01	0.707	0.479	61.10±7.21	61.07±6.96	0.111	0.911
Aspirin, n (%)	988(69.0)	396(68.2)	0.134	0.714	1760(68.8)	879(62.6)	15.742	**<0.001**
Clopidogrel, n (%)	436(30.4)	213(36.7)	7.305	**0.007**	792(31.0)	386(27.5)	5.307	**0.021**

Note: BUN, blood urea nitrogen; UA, uric acid; Cr, creatinine; GLU, glucose; TG, triglyceride; TC, total cholesterol; LDL-C, low density lipoprotein cholesterol; HDL-C, high density lipoprotein cholesterol; CCB, calcium channel blocker; ACEI or ARB, angiotensin-converting enzyme inhibitor or angiotensin receptor blocker; LVEF, left ventricular ejection fractions.

Supplementary Table 2 Baseline treatments and procedure characteristics

Variables	Total				ACS				Stable CAD			
	RPP<10 269 (n=4018)	RPP≥10 269 (n=1997)	X^2or t	P value	RPP<10 269 (n=1444)	RPP≥10 269 (n=585)	X^2or t	P value	RPP<10 269 (n=2574)	RPP≥10 269 (n=1412)	X^2or t	P value
New generation stent, n (%)	3793(94.4)	1875(93.9)	0.699	0.403	1369(94.9)	553(94.5)	0.098	0.754	2424(94.2)	1322(93.6)	0.481	0.488
Diameter of stents, mm	2.85±0.37	2.85±0.37	-0.676	0.499	2.87±0.38	2.85±0.87	0.858	0.391	2.84±0.37	2.86±0.38	-1.548	0.122
Length of stents, mm	28.05±6.96	27.88±6.97	0.843	0.399	28.11±6.96	27.51±6.89	1.778	0.075	28.01±6.96	28.04±7.01	-0.143	0.886
Number of stents, n	1.04±0.22	1.04±0.24	-0.858	0.391	1.04±0.22	1.07±0.28	-1.901	0.057	1.04±0.22	1.04±0.22	0.193	0.847
Pre-dilatation, n (%)	3476(86.5)	1729(86.6)	0.003	0.959	1259(87.2)	505(86.3)	0.314	0.575	2217(86.1)	1224(86.7)	0.238	0.626
Post-dilatation, n (%)	2542(63.3)	1217(60.9)	3.115	0.078	913(63.3)	355(60.7)	1.189	0.276	1629(63.3)	862(61.0)	1.949	0.163
Post-dilatation pressure, atm	13.53±3.59	14.61±3.31	-9.342	**<0.001**	13.41±3.33	14.41±3.45	-5.105	**<0.001**	13.60±3.72	14.68±3.24	-7.658	**<0.001**
CTO, n (%)	873(21.7)	529(26.5)	16.885	**<0.001**	284(19.7)	201(34.4)	49.284	**<0.001**	589(22.9)	328(23.2)	0.062	0.804
ML, n (%)	2573(64.1)	1333(66.8)	4.263	**0.039**	972(67.4)	428(73.2)	6.556	**0.010**	1601(62.2)	905(64.1)	1.402	0.236
Number of vascular lesions, n	1.99±0.84	2.05±0.85	-2.617	**0.009**	2.06±0.84	2.18±0.83	-2.928	**0.003**	1.96±0.84	2.00±0.85	-1.606	0.108

Note: CTO, chronic total occlusion lesions; RPP, rate pressure product; MLs, multi-vessel lesions.

Supplementary Table 3 Outcomes comparison between groups

Outcomes	ACS				Stable CAD			
	RPP<10 269 (n=1444)	RPP≥10 269 (n=585)	X^2	P value	RPP<10 269 (n=2574)	RPP≥10 269 (n=1412)	X^2	P value
ACM, n (%)	58(4.0)	30(5.1)	1.240	0.265	123(4.8)	98(6.9)	8.138	**0.004**
CM, n (%)	49(3.4)	25(4.3)	0.918	0.338	96(3.7)	81(5.7)	8.655	**0.003**
MACCEs, n (%)	198(13.7)	94(16.1)	1.876	0.171	346(13.4)	221(15.7)	3.648	0.056
MACEs, n (%)	181(12.5)	84(14.4)	1.220	0.269	313(12.2)	204(14.4)	4.227	**0.040**

Note: ACM, all-cause mortality; CM, cardiac mortality; MACEs, major adverse cardiovascular events; MACCEs, major adverse cardiovascular and cerebrovascular events.

Supplementary Table 4 Mean Survival Time for Outcomes comparison between groups

Outcomes	Mean Survival Time(m)		
	RPP <10 269 (n=4018)	RPP≥10 269 (n=1997)	Overall
ACM	109.512	103.379	107.681
CM	111.362	105.887	109.731
MACCEs	92.150	85.849	90.204
MACEs	94.267	88.089	92.375

Note: ACM, all-cause mortality; CM, cardiac mortality; MACEs, major adverse cardiovascular events; MACCEs, major adverse cardiovascular and cerebrovascular events.

Supplementary Table 5 Multivariable Cox regression analysis for ACM

Variables	ACS					Stable CAD				
	B	SE	Wald	P values	HR (95% CI)	B	SE	Wald	P values	HR (95% CI)
Age	0.027	0.011	5.473	**0.019**	1.027(1.004-1.050)	0.027	0.007	14.141	**<0.001**	1.027(1.013-1.041)
Male	-0.091	0.295	0.095	0.758	0.913(0.513-1.627)	<0.001	0.173	<0.001	0.998	1.000(0.713-1.404)
Smoking	0.077	0.294	0.069	0.793	1.080(0.607-1.922)	-0.030	0.182	0.028	0.867	0.970(0.679-1.386)
Alcohol drinking	0.124	0.301	0.169	0.681	1.132(0.627-2.044)	-0.056	0.196	0.082	0.774	0.945(0.645-1.387)
Diabetes	0.079	0.275	0.083	0.773	1.082(0.631-1.855)	-0.034	0.178	0.037	0.848	0.966(0.681-1.371)
Hypertension	0.286	0.237	1.458	0.227	1.331(0.837-2.116)	0.098	0.146	0.448	0.503	1.103(0.828-1.470)
GLU	-0.012	0.039	0.099	0.753	0.988(0.915-1.066)	-0.016	0.024	0.461	0.497	0.984(0.939-1.031)
TC	-0.177	0.159	1.231	0.267	0.838(0.613-1.145)	0.175	0.092	3.606	0.058	1.191(0.994-1.427)
BUN	0.051	0.061	0.714	0.398	1.053(0.935-1.185)	0.09	0.038	5.459	0.019	1.094(1.015-1.180)
LDL-C	0.116	0.186	0.389	0.533	1.123(0.779-1.619)	-0.262	0.121	4.717	**0.030**	0.769(0.607-0.975)
Post-dilatation	-0.085	0.231	0.135	0.713	0.919(0.584-1.444)	0.257	0.148	3.005	0.083	1.293(0.967-1.728)
Number of vascular lesions	0.287	0.273	1.111	0.292	1.333(0.781-2.275)	0.190	0.170	1.243	0.265	1.209(0.866-1.687)
CTO	0.600	0.253	5.609	**0.018**	1.823(1.109-2.995)	0.263	0.161	2.681	0.102	1.301(0.949-1.783)
ML	-0.002	0.515	<0.001	0.997	0.998(0.363-2.741)	0.038	0.306	0.016	0.900	1.039(0.570-1.894)
Aspirin	-1.979	0.337	34.553	**<0.001**	0.138(0.071-0.267)	-2.194	0.239	84.425	**<0.001**	0.111(0.070-0.178)
RPP	0.249	0.242	1.058	0.304	1.283(0.798-2.064)	0.473	0.142	11.09	**0.001**	1.605(1.215-2.120)

Note: GLU, glucose; TC, total cholesterol; BUN, blood urea nitrogen; LDL-C, low density lipoprotein cholesterol; CTO, chronic total occlusion lesions; MLs, multi-vessel lesions; RPP, rate pressure product.

Supplementary Table 6 Multivariable Cox regression analysis for CM

Variables	ACS					Stable CAD				
	B	SE	Wald	P values	HR (95% CI)	B	SE	Wald	P values	HR (95% CI)
Age	0.020	0.013	2.456	0.117	1.020(0.995-1.045)	0.017	0.008	4.805	**0.028**	1.017(1.002-1.033)
Male	-0.243	0.324	0.561	0.454	0.784(0.415-1.481)	0.065	0.195	0.110	0.740	1.067(0.728-1.564)
Smoking	-0.182	0.322	0.319	0.572	0.833(0.443-1.568)	-0.102	0.207	0.246	0.620	0.903(0.602-1.353)
Alcohol drinking	0.073	0.337	0.047	0.828	1.076(0.555-2.085)	0.065	0.218	0.089	0.766	1.067(0.696-1.636)
Diabetes	0.216	0.300	0.520	0.471	1.241(0.690-2.234)	0.093	0.197	0.224	0.636	1.098(0.747-1.614)
Hypertension	0.301	0.260	1.337	0.248	1.351(0.811-2.249)	-0.024	0.166	0.021	0.884	0.976(0.706-1.350)
GLU	-0.067	0.051	1.755	0.185	0.935(0.847-1.033)	-0.030	0.028	1.156	0.282	0.971(0.919-1.025)
TC	0.024	0.189	0.015	0.901	1.024(0.707-1.483)	0.178	0.104	2.936	0.087	1.195(0.975-1.464)
BUN	0.070	0.065	1.159	0.282	1.073(0.944-1.219)	0.127	0.042	9.039	**0.003**	1.135(1.045-1.232)
LDL-C	-0.078	0.231	0.115	0.735	0.925(0.587-1.455)	-0.242	0.136	3.173	0.075	0.785(0.602-1.025)
Post-dilatation	-0.066	0.255	0.066	0.797	0.936(0.568-1.544)	0.255	0.166	2.358	0.125	1.291(0.932-1.789)
Number of vascular lesions	0.456	0.310	2.165	0.141	1.577(0.860-2.894)	0.264	0.191	1.907	0.167	1.302(0.895-1.893)
CTO	0.629	0.279	5.057	**0.025**	1.875(1.084-3.242)	0.338	0.178	3.613	0.057	1.403(0.990-1.989)
ML	-0.376	0.585	0.413	0.520	0.686(0.218-2.162)	-0.030	0.348	0.007	0.932	0.971(0.491-1.920)
Aspirin	-1.937	0.368	27.721	**<0.001**	0.144(0.070-0.297)	-2.116	0.255	68.802	**<0.001**	0.121(0.073-0.199)
RPP	0.249	0.268	0.862	0.353	1.282(0.758-2.168)	0.550	0.160	11.867	**0.001**	1.733(1.267-2.369)

Note: GLU, glucose; TC, total cholesterol; BUN, blood urea nitrogen; LDL-C, low density lipoprotein cholesterol; CTO, chronic total occlusion lesions; MLs, multi-vessel lesions; RPP, rate pressure product.

Supplementary Table 7 Multivariable Cox regression analysis for MACCEs

Variables	ACS					Stable CAD				
	B	SE	Wald	P values	HR (95% CI)	B	SE	Wald	P values	HR (95% CI)
Age	-0.001	0.006	0.037	0.847	0.999(0.987-1.011)	-0.002	0.004	0.249	0.617	0.998(0.989-1.006)
Male	-0.060	0.155	0.150	0.698	0.942(0.694-1.277)	-0.182	0.115	2.529	0.112	0.833(0.665-1.043)
Smoking	-0.298	0.160	3.441	0.064	0.743(0.542-1.017)	-0.158	0.112	2.015	0.156	0.854(0.686-1.062)
Alcohol drinking	-0.031	0.169	0.033	0.857	0.970(0.696-1.351)	-0.054	0.118	0.208	0.649	0.948(0.752-1.194)
Diabetes	0.078	0.152	0.261	0.610	1.081(0.803-1.455)	0.207	0.106	3.817	0.051	1.230(0.999-1.514)
Hypertension	0.384	0.128	8.964	**0.003**	1.468(1.142-1.887)	0.252	0.091	7.566	**0.006**	1.286(1.075-1.539)
GLU	-0.023	0.023	0.977	0.323	0.978(0.935-1.023)	<0.001	0.014	0.001	0.978	1.000(0.972-1.028)
TC	-0.051	0.094	0.287	0.592	0.951(0.790-1.144)	0.048	0.065	0.553	0.457	1.049(0.925-1.191)
BUN	0.054	0.034	2.537	0.111	1.055(0.988-1.128)	0.052	0.025	4.272	0.039	1.054(1.003-1.107)
LDL-C	-0.036	0.114	0.099	0.753	0.965(0.771-1.207)	-0.158	0.081	3.796	0.051	0.854(0.728-1.001)
Post-dilatation	0.077	0.129	0.354	0.552	1.080(0.838-1.391)	0.038	0.090	0.181	0.671	1.039(0.871-1.239)
No. of vascular lesions	0.256	0.151	2.865	0.091	1.291(0.960-1.736)	0.177	0.108	2.696	0.101	1.194(0.966-1.476)
CTO	0.178	0.149	1.423	0.233	1.195(0.892-1.602)	0.203	0.102	3.928	**0.047**	1.225(1.002-1.497)
ML	-0.095	0.276	0.117	0.732	0.910(0.529-1.564)	-0.035	0.192	0.034	0.854	0.965(0.662-1.407)
Aspirin	-0.463	0.141	10.742	**0.001**	0.629(0.477-0.830)	-0.507	0.101	25.196	**<0.001**	0.602(0.494-0.734)
RPP	0.196	0.136	2.088	0.148	1.216(0.933-1.587)	0.239	0.091	6.956	**0.008**	1.271(1.063-1.518)

Note: GLU, glucose; TC, total cholesterol; BUN, blood urea nitrogen; LDL-C, low density lipoprotein cholesterol; CTO, chronic total occlusion lesions; MLs, multi-vessel lesions; RPP, rate pressure product.

Supplementary Table 8 Multivariable Cox regression analysis for MACEs

Variables	ACS					Stable CAD				
	B	SE	Wald	P values	HR (95% CI)	B	SE	Wald	P values	HR (95% CI)
Age	-0.003	0.006	0.201	0.654	0.997(0.985-1.01)	-0.005	0.005	1.069	0.301	0.995(0.986-1.004)
Male	-0.051	0.164	0.095	0.758	0.951(0.689-1.312)	-0.147	0.121	1.47	0.225	0.864(0.681-1.095)
Smoking	-0.230	0.167	1.894	0.169	0.795(0.573-1.102)	-0.101	0.116	0.751	0.386	0.904(0.720-1.136)
Alcohol drinking	-0.045	0.176	0.066	0.797	0.956(0.677-1.349)	-0.052	0.122	0.183	0.669	0.949(0.747-1.206)
Diabetes	0.068	0.160	0.181	0.670	1.070(0.783-1.464)	0.194	0.111	3.059	0.080	1.214(0.977-1.510)
Hypertension	0.395	0.134	8.625	**0.003**	1.484(1.140-1.932)	0.247	0.096	6.622	**0.010**	1.280(1.061-1.544)
GLU	-0.027	0.024	1.229	0.268	0.973(0.928-1.021)	0.002	0.015	0.019	0.889	1.002(0.974-1.031)
TC	-0.055	0.100	0.303	0.582	0.947(0.778-1.151)	0.050	0.067	0.568	0.451	1.052(0.923-1.199)
BUN	0.050	0.036	1.915	0.166	1.051(0.980-1.127)	0.045	0.027	2.832	0.092	1.046(0.993-1.102)
LDL-C	-0.034	0.121	0.078	0.780	0.967(0.763-1.225)	-0.142	0.084	2.828	0.093	0.868(0.736-1.024)
Post-dilatation	0.005	0.134	0.001	0.971	1.005(0.773-1.307)	0.060	0.094	0.406	0.524	1.062(0.883-1.278)
No. of vascular lesions	0.307	0.158	3.804	0.051	1.360(0.998-1.852)	0.153	0.112	1.864	0.172	1.166(0.935-1.453)
CTO	0.200	0.155	1.668	0.197	1.221(0.902-1.654)	0.240	0.106	5.093	**0.024**	1.271(1.032-1.565)
ML	-0.096	0.291	0.109	0.741	0.908(0.514-1.607)	0.029	0.200	0.021	0.884	1.030(0.696-1.524)
Aspirin	-0.551	0.147	13.991	**<0.001**	0.576(0.432-0.769)	-0.597	0.106	31.853	**<0.001**	0.551(0.448-0.677)
RPP	0.17	0.143	1.423	0.233	1.186(0.896-1.569)	0.274	0.095	8.326	**0.004**	1.315(1.092-1.584)

Note: GLU, glucose; TC, total cholesterol; BUN, blood urea nitrogen; LDL-C, low density lipoprotein cholesterol; CTO, chronic total occlusion lesions; MLs, multi-vessel lesions; RPP, rate pressure product.

Supplementary Table 9 ROC analysis of different parameters for CM

Variables	AUC (95%CI)	Difference between areas (95%CI)	Z value	P value
RPP	0.586(0.573-0.598)	-	**-**	-
HR	0.554(0.541-0.566)	0.032(0.003-0.061)	2.183	**0.029**
SBP	0.511(0.498-0.524)	0.075(0.025-0.125)	2.923	**0.004**

Note: RPP, rate pressure product; HR, heart rate; SBP, systolic blood pressure, AUC, area under curve.
