# Supplementary material for: Rate pressure product as a novel predictor of long-term adverse outcomes in patients after percutaneous coronary intervention: a retrospective cohort study
Source: BMJ Open. 2023 Apr 4;13(4):e067951. doi: 10.1136/bmjopen-2022-067951 (PMC10083747; doi:10.1136/bmjopen-2022-067951)
Supplement: Author's manuscript [file bmjopen-2022-067951.draft_revisions.pdf]

BMJ Open is committed to open peer review. As part of this commitment we make the peer review history of every article we publish publicly available.

When an article is published we post the peer reviewers' comments and the authors' responses online. We also post the versions of the paper that were used during peer review. These are the versions that the peer review comments apply to.

The versions of the paper that follow are the versions that were submitted during the peer review process. They are not the versions of record or the final published versions. They should not be cited or distributed as the published version of this manuscript.

BMJ Open is an open access journal and the full, final, typeset and author-corrected version of record of the manuscript is available on our site with no access controls, subscription charges or pay-per-view fees (http://bmjopen.bmj.com).

If you have any questions on BMJ Open's open peer review process please email info.bmjopen@bmj.com

BMJ Open

Rate pressure product as a novel predictor of long-term adverse outcomes in patients after percutaneous coronary intervention: A retrospective cohort study

Journal:	*BMJ Open*
Manuscript ID	bmjopen-2022-067951
Article Type:	Original research
Date Submitted by the Author:	13-Sep-2022
Complete List of Authors:	Jiang, Zhi-Hui; First Affiliated Hospital of Xinjiang Medical University, Department of Cardiology<br>Aierken, Abudula; Xinjiang Medical University Affiliated First Hospital<br>Wu, Ting-Ting; Xinjiang Medical University Affiliated First Hospital<br>Zheng, Ying-Ying; Xinjiang Medical University Affiliated First Hospital<br>Ma, Yi-Tong ; First Affiliated Hospital of Xingjiang Medical University<br>Xie, Xiang; Xinjiang Medical University Affiliated First Hospital, Department of Cardiology
Keywords:	Coronary heart disease < CARDIOLOGY, Coronary intervention < CARDIOLOGY, Cardiology < INTERNAL MEDICINE

SCHOLARONE™
Manuscripts

I, the Submitting Author has the right to grant and does grant on behalf of all authors of the Work (as defined in the below author licence), an exclusive licence and/or a non-exclusive licence for contributions from authors who are: i) UK Crown employees; ii) where BMJ has agreed a CC-BY licence shall apply, and/or iii) in accordance with the terms applicable for US Federal Government officers or employees acting as part of their official duties; on a worldwide, perpetual, irrevocable, royalty-free basis to BMJ Publishing Group Ltd ("BMJ") its licensees and where the relevant Journal is co-owned by BMJ to the co-owners of the Journal, to publish the Work in this journal and any other BMJ products and to exploit all rights, as set out in our licence.

The Submitting Author accepts and understands that any supply made under these terms is made by BMJ to the Submitting Author unless you are acting as an employee on behalf of your employer or a postgraduate student of an affiliated institution which is paying any applicable article publishing charge ("APC") for Open Access articles. Where the Submitting Author wishes to make the Work available on an Open Access basis (and intends to pay the relevant APC), the terms of reuse of such Open Access shall be governed by a Creative Commons licence – details of these licences and which Creative Commons licence will apply to this Work are set out in our licence referred to above.

Other than as permitted in any relevant BMJ Author's Self Archiving Policies, I confirm this Work has not been accepted for publication elsewhere, is not being considered for publication elsewhere and does not duplicate material already published. I confirm all authors consent to publication of this Work and authorise the granting of this licence.

For peer review only

Rate pressure product as a novel predictor of long-term adverse outcomes in patients after percutaneous coronary intervention: A retrospective cohort study

Zhi-Hui Jiang[1], Abudula Aierken[1], Ting-Ting Wu[1], Ying-Ying Zheng[1,2], Yi-Tong Ma[1], Xiang Xie[1*]

[*] Correspondence: xiangxie999@sina.com

[1] Department of Cardiology, First Affiliated Hospital of Xinjiang Medical University, Urumqi 830011, People's Republic of China

[2] Department of Cardiology, First Affiliated Hospital of Zhengzhou University, Zhengzhou 450052, People's Republic of China

For peer review only

Abstract

Objectives:

Previous studies have suggested that heart rate and blood pressure play important roles in the development of adverse outcomes in patients with coronary artery disease (CAD) who underwent percutaneous coronary intervention (PCI). However, the relationship between the rate pressure product (RPP) and long-term outcomes has rarely been investigated. This study investigated the effects of RPP on the clinical outcomes of CAD patients who underwent PCI.

Methods:

In this study, a total of 6,015 CAD patients undergoing PCI were enrolled. All patients were from the CORFCHD-PCI study. They were divided into two groups according to RPP (RPP<10,269, *n*=4,018 and RPP≥10,269, *n*=1,997). In addition, the median follow-up time was 32 months. The primary endpoint was long-term mortality, including all-cause mortality (ACM) and cardiac mortality (CM). The secondary endpoints were major adverse cardiovascular events (MACEs) and major adverse cardiovascular and cerebrovascular events (MACCEs).

Results:

We found that there were significant differences between the two groups in the incidences of ACM, CM, MACCEs and MACEs (all *P*<0.05). Among the CAD patients with ACM, CM, MACCEs and MACEs, the mean survival time of the low-value group was significantly higher than that of the high-value group. After multivariate Cox regression analysis, the respective risks of ACM (hazard ratio (*HR*)=1.598, 95% confidence interval (*CI*): 1.262-2.024, *P*<0.001), CM (*HR*=1.678, 95% *CI*: 1.289-2.184, *P*<0.001), MACCEs (*HR*=1.263, 95% *CI*: 1.092-1.462, *P*=0.002) and MACEs (*HR*=1.281, 95% *CI*: 1.099-1.492, *P*=0.001) that occurred in the high-value group were still significantly higher than those in the low value group.

Conclusion:

In summary, rate pressure product is an independent predictor of long-term prognosis in CAD patients undergoing PCI. A higher baseline RPP before PCI increased the risk of adverse outcomes. Compared with heart rate and blood pressure alone, RPP has a higher predictive value for adverse clinical outcomes.

Keywords: Coronary artery disease, Rate pressure product, Mortality, Outcomes, Percutaneous coronary intervention

Strengths and limitations of this study

There are many advantages. The first is the larger sample size, which can improve the

scientific nature of the statistics. The second is the long follow-up time, which can further improve the reliability of the statistical results. The third is that RPP combines the information of two indicators and is simple and easy to obtain. However, its limitations cannot be ignored. First, we only collected heart rate and blood pressure date at the first hospitalization, thus lacking information on dynamic changes in these variables. This study had a single-centre retrospective cohort design, and our results need to be further validated through large multi-centre randomized controlled experiments. Finally, follow-up was mainly based on telephone contacts, which may bias the events.

Introduction

Coronary atherosclerotic heart disease refers to the occurrence of atherosclerosis in the coronary arteries, which narrows or obstructs the arteries, causing local myocardial ischemia, hypoxia, and even necrosis, resulting in changes in heart structure and loss of function. Together with coronary artery spasms are collectively called coronary artery disease (CAD)[1]. CAD is one of the main causes of death and disability in China, and is a serious threat to health. The WHO classifies CAD into 5 clinical types: silent myocardial ischemia (latent coronary artery disease), angina pectoris, myocardial infarction, ischemia heart failure (ischemic heart disease) and sudden cardiac death. The related risk factors include sex, age, smoking, insufficient exercise, obesity, diabetes, dyslipidemia and metabolic disorders, hypertension, family history of CAD and others[2-5]. At present, percutaneous coronary intervention (PCI) is one of the most important methods for the treatment of coronary artery disease, and can significantly reduce the mortality and risk of reischemic attacks[6-8]. As an established measure of myocardial load, rate pressure product (RPP) may provide a more reliable assessment of cardiac workload[9]. Previous studies have shown that RPP can be used as an auxiliary reference index for coronary artery disease, which is negatively correlated with the degree of coronary artery disease and the number of diseased branches and negatively correlated with age and sex[10]. RPP indirectly reflects myocardial blood supply and oxygen consumption. Cook et al. [11] found that PCI can increase myocardial workload. The change observed immediately following PCI was caused by the abolition of stenosis resistance. Yazdani et al.[12] found that compared with systolic blood pressure (SBP), diastolic blood pressure (DBP), and heart rate (HR), RPP was a stronger predictor of all-cause mortality (ACM) and cardiac mortality (CM) in the general population and in patients with coronary artery disease, especially in the cohort of patients at high risk of cardiovascular disease. Interestingly, RPP also predicted cardiac mortality in heart failure with preserved ejection fraction (HFrEF) patients in a stronger way, while SBP and HR alone showed no significant association. Furthermore, in 3-vessel CAD patients RPP was a stronger predictor of cardiac mortality than SBP or HR alone. Therefore, especially in patients with severe coronary artery disease and heart failure, the cardiac workload given as RPP should be considered in clinical practice[12]. However, they did not clarify the predictability of RPP for the prognosis of CAD patients who underwent PCI. RPP is the product of HR and SBP, because it combines the information of the two indicators, and both indicators are related to the prognosis of CAD patients. Therefore, we speculate that RPP may affect the long-term prognosis of CAD patients who underwent PCI. Compared with HR and SBP, RPP may have a higher predictive value for adverse clinical outcomes.

In this research, 6,015 CAD patients were included to investigate the relationship between the RPP and clinical outcomes.

For peer review only - http://bmjopen.bmj.com/site/about/guidelines.xhtml

2. Methods

2.1 Study design and population

In this study, 6,050 CAD patients who were hospitalized at the First Affiliated Hospital of Xinjiang Medical University from January 2008 to December 2016 were evaluated initially. Regarding the inclusion criteria, coronary angiography confirmed that at least one major coronary artery showed stenosis ≥70%, including the left main coronary artery, left anterior descending artery, left circumflex artery, and right coronary artery, or other more important branches. All patients underwent PCI and received at least one stent via implantation. Regarding the exclusion criteria, first, patients with the presence of acute infections, malignant tumor, hepatobiliary disease, alcohol abuse or blood disease were excluded. Second, patients who had incomplete clinical or follow-up data were also excluded. In the end, 6,015 CAD patients were enrolled in the present study. All CAD patients were from the Clinical Outcomes and Risk Factors of Patients with Coronary Heart Disease after PCI (CORFCHD-PCI) study. The study is a large single-centre retrospective cohort study based on medical records and the follow-up information collected at the First Affiliated Hospital of Xinjiang Medical University. The CORFCHD-PCI study complied with the Declaration of Helsinki and the protocol was approved by the Ethics Committee of the First Affiliated Hospital of Xinjiang Medical University. The details of the design have been registered at http://www.chictr.org.cn (identifier: ChiCTR-ORC-16010153). Since the study was designed retrospectively, the Ethics Committee considered that it was not necessary to obtain informed consent form the eligible patients. **Figure 1** shows the flow chart of the inclusion and exclusion criteria for the selection of 6,050 CAD patients undergoing PCI.

2.2 Data collection

The cardiovascular-related risk factors, clinical data, laboratory data and interventional therapy data of all patients were collected retrospectively, including: age, sex, heart rate, blood pressure, smoking status, alcohol consumption, history of diagnosed diabetes, history of hypertension, family history of CAD, history of medication, blood urea nitrogen (BUN), serum creatinine (Cr), uric acid (UA), glucose (GLU), triglycerides (TG), total cholesterol (TC), low density lipoprotein cholesterol (LDL-C), and high-density lipoprotein cholesterol (HDL-C). The purpose of this study was to investigate the relationship between the RPP and clinical outcomes. For this reason, HR and blood pressure at admission were measured accurately at the first medical contact by physicians using calibrated mercury sphygmomanometers. The RPP was defined as the product of SBP (mmHg) and HR (beats per minute). All laboratory tests were carried out in the Medical Laboratory Centre of the First Affiliated Hospital of Xinjiang Medical University.

2.3 Clinical diagnosis

The diagnostic criteria for diabetes mellitus were a clear history of diabetes, the use of hypoglycemic agents, fasting plasma glucose ≥7.0 mmol/L, 2-h plasma glucose ≥11.1 mmol/L during oral glucose tolerance test (OGTT), A1C ≥ 6.5%（48 mmol/mol）, or a random plasma glucose ≥11.1 mmol/L in a patient with classic symptoms of hyperglycemia or hyperglycemic crisis[13]. Hypertension was diagnosed when SBP was ≥140 mmHg and/or diastolic blood pressure (DBP) was ≥ 90 mmHg following repeated examination, or treatment with antihypertensive drugs[14].

2.4 Endpoints

The primary endpoint was long-term mortality, including long-term all-cause mortality (ACM) and cardiac mortality (CM). The secondary endpoints were major adverse cardiovascular events (MACEs) and major adverse cardiovascular and cerebrovascular

For peer review only - http://bmjopen.bmj.com/site/about/guidelines.xhtml

events (MACCEs). MACEs referred to the composite endpoint of cardiac death, recurrent myocardial infarction and target vessel reconstruction. MACCEs was defined as MACEs plus nonfatal stroke[15].

2.5 Follow-Up

In this study, 6,050 CAD patients started receiving follow-up when they underwent percutaneous coronary intervention, and regular follow-up was performed at the end of 1 month, 3 months, 6 months, 1 year, 3 years, and 5 years after discharge. Outpatient review and telephone were utilized to follow-up until the end point events occurred or the study was concluded. The median follow-up time was 32 months, and the longest follow-up time was 10 years.

2.6 Statistical analyses

All statistical analyses were performed using SPSS version 26.0 (SPSS Inc, Chicago, Illinois, United States). Continuous data are presented as the mean ± standard deviation ($\bar{x}$ ± s), and categorical variables are presented as frequencies and percentages. RPP is a continuous numerical variable. Because there is no established optimal threshold for RPP, receiver operator characteristic (ROC) curve analysis was performed to assess the predictive ability of RPP in clinical outcomes. The point where the sensitivity and specificity were maximized was determined as the best cut-off point. Two samples *t*-tests or chi-square tests were used to compare the clinical data of patients in different groups and the characteristics of adverse outcomes. Kaplan-Meier analysis was used to compare the cumulative incidence rates of long-term adverse outcomes and the log-rank test was used to compare between groups. Multivariate Cox regression analysis was performed to evaluate the predictive value of RPP for clinical outcomes. The fully adjusted models for RPP included age, hypertension, diabetes mellitus, sex, GLU, LDL-C, TC, smoking, alcohol consumption. Hazard ratios (*HRs*) and 95% confidence intervals (*CIs*) were also calculated. A *P*-Value of < 0.05 was considered statistically significant.

Patient and Public Involvement: No patient involved.

3. Results

3.1 Baseline data and procedural characteristics

Among the 6,015 patients included in this study, 74.3% were males, 40.0% were smokers, 29.3% were drinkers, 24.1% had diabetes mellitus, 42.3% had hypertension, and the mean age of the patients was 59.48 years. During the follow-up period, a history of using calcium antagonists accounted for 11.5%, and patients using β-receptor blockers, ACEI/ARB drugs and statins accounted for 40.4%, 22.8% and 54.4%, respectively. During the 10-year follow-up, 309 (5.1%) deaths occurred, of which 251 (4.2%) patients died due to cardiovascular disease. MACCEs occurred in 859 (14.3%) patients, including 782 (13.0%) MACEs and 77 (1.28%) nonfatal strokes.

In this study, the ROC curve analysis showed that a RPP value of 10,269 was the cut-off with the highest sensitivity and specificity in terms of prognostic significance. RPP<10,269 was considered the low-value group, and RPP≥10,269 was considered the high-value group. The population clinical characteristics were grouped by dichotomy of RPP, which was based on the ROC cut-off (as shown in **Table 1**). There were 4,018 CAD patients in the low-value group and 1,997 CAD patients in the high-value group. In this study, we found that there were significant differences in many variables between the two groups (all *P*<0.05), specifically, age (*P*<0.001), BUN (*P*=0.023), GLU (*P*<0.001), TC (*P*=0.021), and LDL-C (*P*=0.007) in the high-value group were higher than those in the low-value group. The number of diabetes mellitus and hypertension patients was also higher in the high-value group than the low-value group.

For peer review only - http://bmjopen.bmj.com/site/about/guidelines.xhtml

Sex (P<0.001), smoking (P<0.001), alcohol consumption (P=0.014) and the number of statins used (P=0.019) in the high-value group were lower than those in the low-value group. We did not find statistically significant differences between the two groups in terms of Cr, UA, TG, HDL-C, or history of using CCB drugs, β-receptor blocker drugs or ACBI/ARB drugs (all P >0.05).

Table 1 Characteristics of participants of the two groups

Variables	RPP<10269 (*n*=4018)	RPP≥10269 (*n*=1997)	x^2or *t*	*P* value
Age, years	58.92±10.90	60.62±10.61	-5.751	**<0.001**
Male, n (%)	3057 (76.1)	1415 (70.9)	19.105	**<0.001**
Smoking, n (%)	1686 (42.0)	718 (36.0)	20.064	**<0.001**
Alcohol drinking, n (%)	1217 (30.3)	544 (27.2)	5.985	**0.014**
Diabetes, n (%)	847 (21.1)	600 (30.0)	58.684	**<0.001**
Hypertension, n (%)	1478 (36.8)	1067 (53.4)	151.429	**<0.001**
BUN, mmol/L	5.48±1.65	5.59±1.72	-2.271	**0.023**
Cr, umol/L	75.78±19.55	76.03±21.97	-0.447	0.655
UA, mmol/L	323.96±89.56	322.21±91.03	0.697	0.486
GLU, mmol/L	6.41±2.99	6.92±3.39	-5.888	**<0.001**
TG, mmol/L	1.88±1.23	1.94±1.35	-1.751	0.080
TC, mmol/L	3.94±1.10	4.01±1.13	-2.305	**0.021**
LDL-C, mmol/L	2.44±0.90	2.51±0.95	-2.689	**0.007**
HDL-C, mmol/L	1.02±0.46	1.02±0.52	-0.196	0.845
CCB, n (%)	462 (11.6)	227 (11.4)	0.022	0.883
β-blockers, n (%)	1631 (40.8)	785 (39.5)	0.930	0.335
ACEI or ARB, n (%)	894 (22.4)	472 (23.8)	1.525	0.217
Statins, n (%)	2207 (55.5)	1035 (52.2)	5.480	**0.019**

Note: *BUN, Blood urea nitrogen; UA, Uric acid; Cr, Creatinine; GLU, Glucose; TG, Triglyceride; TC, Total cholesterol; LDL-C, Low density lipoprotein cholesterol; HDL-C, High density lipoprotein cholesterol.*

3.2 Clinical outcomes

A comparison of clinical outcomes between the two groups of CAD patients is shown in **Table 2**. We found that, in terms of major endpoints, the incidence rate of all-cause death was 6.4% in the high-value group and 4.5% in the low-value group, which showed that ACM in the high-value group was higher than that in the low-value group. CM in the high and low value group was 5.3% and 3.6%, respectively, and CM in the high-value group was also higher than that in the low-value group. The differences that we were discussed above, were statistically significant (all P=0.002). For the secondary endpoints, the incidence rates of MACCEs in the high-value group and the low-value group were 15.8% and 13.5%, respectively, while the incidence rates of MACEs were 14.4% and 12.3%, respectively. That is, the MACCEs and MACEs in the high-value group were also higher than those in the low-value group, and the differences were statistically significant (all P<0.05).

Table 2. Outcomes comparison between groups.

Outcomes	RPP<10269 (*n*=4018)	RPP≥10269 (*n*=1997)	x^2	*P* value
ACM, n (%)	181(4.5)	128 (6.4)	9.933	**0.002**

For peer review only

CM, n (%)	145 (3.6)	106 (5.3)	9.632	**0.002**
MACCE, n (%)	544 (13.5)	315 (15.8)	5.441	**0.020**
MACE, n (%)	494 (12.3)	288 (14.4)	5.336	**0.021**

Note: *ACM, All-cause mortality; CM, Cardiac mortality; MACE, Major adverse cardiovascular events; MACCE, Major adverse cardiovascular and cerebrovascular events.*

In this study, baseline RPP before PCI was used as an independent variable, and ACM, CM, MACEs and MACCEs were used as the endpoints. Kaplan-Meier analysis was performed, and the log-rank test was used for comparisons between groups. The results are shown in **Table 3**. Among the CAD patients with ACM, CM, MACCEs and MACEs, the mean survival time of the low-value group was significantly higher than that of the high-value group. As shown in **Figure 2**, with the extension of the follow-up time, the cumulative survival rate of the patients showed a downward trend, and the decreasing rate of the high-value group was more significant than that of the low-value group. While the cumulative risk showed an upward trend, the increasing rate of the high-value group was significantly higher than that of the low-value group. The above differences were statistically significant (all P<0.001).

Table 3. Mean Survival Time for Outcomes comparison between groups.

Outcomes	Mean Survival Time（m）		
	RPP <10269 (n=4018)	RPP≥10269 (n=1997)	Overall
ACM	109.512	103.379	107.681
CM	111.362	105.887	109.731
MACCE	92.150	85.849	90.204
MACE	94.267	88.089	92.375

Note: *ACM, All-cause mortality; CM, Cardiac mortality; MACE, Major adverse cardiovascular events; MACCE, Major adverse cardiovascular and cerebrovascular events.*

Tables 4-7 show select variables based on the aforementioned analysis. The results of collinearity analysis showed that there was no significant collinearity among age, hypertension, diabetes mellitus, sex, GLU, LDL-C, TC, smoking, alcohol consumption and RPP (all VIF<10). In the multivariate Cox regression analysis of ACM and CM, only age and RPP significantly positively affected mortality. That is, as age or the RPP value increased, the long-term mortality rate of CAD patients who underwent PCI also increased. In the multivariate Cox regression analysis of MACCEs, hypertension, diabetes mellitus and RPP significantly positively affected the incidence rate of MACCEs, while smoking significantly negatively affected their incidence rate. In the MACEs multivariate Cox regression analysis, hypertension and RPP also significantly positively affected the incidence rate. Multivariate Cox regression analysis was performed to assess the prognostic value of the RPP for adverse outcomes after adjusting for the influence of age, hypertension, diabetes mellitus, sex, GLU, LDL-C, TC, smoking, alcohol consumption and other confounding factors. In total, the results

For peer review only

showed that higher preoperative RPP value was a risk factor for clinical outcomes in CAD patients undergoing PCI. The respective risks of ACM (*HR*=1.598, 95% *CI*: 1.262-2.024, *P*<0.001), CM (*HR*=1.678, 95% *CI*: 1.289-2.184, *P*<0.001), MACCEs (*HR*=1.263, 95% *CI*: 1.092-1.462, P=0.002) and MACEs (*HR*=1.281, 95% *CI*: 1.099-1.492, *P*=0.001) that occurred in the high-value group were still significantly higher than those in the low value group.

Table 4. Multivariable Cox regression analysis for ACM.

Variables	B	SE	Wald	*P* values	*HR* (95% *CI*)
Age	0.029	0.006	23.420	**0.000**	1.029 (1.017-1.041)
Hypertension	0.140	0.123	1.292	0.256	1.150 (0.904-1.463)
GLU	-0.002	0.020	0.008	0.927	0.998 (0.960-1.038)
Diabetes	0.005	0.148	0.001	0.971	1.005 (0.752-1.344)
Gender	-0.056	0.147	0.144	0.704	0.946 (0.709-1.262)
LDL-c	-0.183	0.108	2.865	0.091	0.833 (0.673-1.029)
TC	0.103	0.087	1.402	0.236	1.108 (0.935-1.313)
Smoking	0.010	0.154	0.004	0.949	1.010 (0.747-1.366)
Alcohol	-0.056	0.163	0.119	0.730	0.945 (0.687-1.301)
RPP	0.469	0.121	15.098	**0.000**	1.598 (1.262-2.024)

Note: *GLU, Glucose; TC, Total cholesterol; LDL-C, Low density lipoprotein cholesterol; RPP, Rate pressure product.*

Table 5. Multivariable Cox regression analysis for CM.

Variables	B	SE	Wald	*P* value	*HR* (95% *CI*)
Age	0.021	0.007	10.244	**0.001**	1.021 (1.008-1.034)
Hypertension	0.069	0.138	0.254	0.614	1.072 (0.818-1.405)
GLU	-0.023	0.024	0.899	0.343	0.978 (0.933-1.024)
Diabetes	0.143	0.163	0.776	0.378	1.154 (0.839-1.588)
Gender	-0.073	0.164	0.196	0.658	0.930 (0.674-1.283)
LDL-c	-0.227	0.118	3.706	0.054	0.797 (0.633-1.004)
TC	0.162	0.093	3.017	0.082	1.176 (0.979-1.413)
Smoking	-0.108	0.173	0.391	0.532	0.897 (0.639-1.260)
Alcohol	0.002	0.182	0.000	0.990	1.002 (0.701-1.432)
RPP	0.518	0.134	14.812	**0.000**	1.678 (1.289-2.184)

Note: *GLU, Glucose; TC, Total cholesterol; LDL-C, Low density lipoprotein cholesterol; RPP, Rate pressure product.*

Table 6. Multivariable Cox regression analysis for MACCEs.

Variables	B	SE	Wald	*P* value	*HR* (95% *CI*)
Age	0.000	0.003	0.000	0.991	1.000 (0.993-1.007)
Hypertension	0.294	0.074	15.816	**0.000**	1.341 (1.161-1.550)
GLU	0.001	0.012	0.010	0.922	1.001 (0.978-1.025)
Diabetes	0.173	0.086	4.043	**0.044**	1.189 (1.004-1.408)
Gender	-0.177	0.091	3.740	0.053	0.838 (0.701-1.002)
LDL-c	-0.130	0.067	3.727	0.054	0.878 (0.770-1.002)
TC	0.028	0.054	0.267	0.605	1.029 (0.924-1.144)
Smoking	-0.190	0.091	4.339	**0.037**	0.827 (0.691-0.989)
Alcohol	-0.075	0.096	0.603	0.437	0.928 (0.768-1.121)
RPP	0.234	0.074	9.840	**0.002**	1.263 (1.092-1.462)

For peer review only

Note: GLU, Glucose; TC, Total cholesterol; LDL-C, Low density lipoprotein cholesterol; RPP, Rate pressure product.

Table 7. Multivariable Cox regression analysis for MACEs.

Variables	B	SE	Wald	*P* value	*HR* (95% *CI*)
Age	-0.002	0.004	0.404	0.525	0.998 (0.991-1.005)
Hypertension	0.291	0.077	14.127	**0.000**	1.337 (1.149-1.556)
GLU	0.002	0.012	0.039	0.844	1.002 (0.978-1.027)
Diabetes	0.164	0.090	3.298	0.069	1.179 (0.987-1.407)
Gender	-0.147	0.096	2.315	0.128	0.864 (0.715-1.043)
LDL-c	-0.119	0.070	2.906	0.088	0.887 (0.774-1.018)
TC	0.031	0.057	0.290	0.590	1.031 (0.923-1.152)
Smoking	-0.125	0.095	1.720	0.190	0.883 (0.732-1.064)
Alcohol	-0.084	0.100	0.713	0.399	0.919 (0.755-1.118)
RPP	0.248	0.078	10.090	**0.001**	1.281 (1.099-1.492)

Note: GLU, Glucose; TC, Total cholesterol; LDL-C, Low density lipoprotein cholesterol; RPP, Rate pressure product.

4. Discussion

Among CAD patients who underwent PCI, higher preoperative RPP value led to 59.8%, 67.8%, 26.3%, and 28.1% higher relative risks in ACM, CM, MACCEs and MACEs, respectively. Compared with heart and blood pressure alone, RPP has a higher predictive value for adverse clinical outcomes.

Rate pressure product is a very reliable indicator of myocardial oxygen uptake (MVO_2). MVO_2 depends on systolic blood pressure, heart rate and ventricular wall tension. RPP is often used to evaluate workload and exercise load, and an increase in RPP indicates that subjects need to increase MVO_2 to meet the metabolic needs of the body[16, 17]. RPP in healthy normotensive young adults has a normative circadian pattern, and the RPP value is greater during the day than at night, especially in the afternoon. The circadian pattern of THE large amplitude in the RPP and its sex-based differences must be taken into account when using the RPP to assess cardiac workload, risk of left ventricular hypertrophy, and efficiency of antihypertensive therapy[18]. Stegehuis et al.[19] performed intracoronary doppler flow velocity measurements to obtain Coronary Flow Reserve (CFR) and Coronary Flow Capacity (CFC) after inducing hyperemia in 390 non-obstructed vessels of patients who were scheduled for elective percutaneous coronary intervention of another vessel. Akaike's Information Criterion (AIC) revealed that the RPP is an independent predictor of CFR and CFC. Since CFR and CFC are physiological indices for the assessment of myocardial blood flow damage caused by coronary artery disease, we can clarify the correlation between the RPP and the prognosis of CAD patients who underwent PCI. Furthermore, Pathophysiological studies indicate that a relatively high HR has direct detrimental effects on the progression of coronary atherosclerosis, on the occurrence of myocardial ischemia and ventricular arrhythmias, and on left ventricular function[20]. Previous studies have demonstrated that elevated heart rate was an independent risk factor for long-term adverse prognosis in CAD patients undergoing PCI who had postoperative stable angina or acute coronary syndrome[21-23]. O'Brien et al.[24] observed a correlation between HR and the prognosis of CAD patients who underwent PCI and found that HR before PCI was an independent predictor of adverse 30-day cardiovascular outcomes. Systolic blood pressure was found to be a risk factor for cardiac death in CAD patients.

For peer review only

Blood pressure before PCI was associated with long-term prognosis in CAD patients undergoing PCI, and the long-term mortality rate of CAD patients with low systolic and high throbbing pressure was significantly reduced. In other words, a wider pulse pressure could be used as a risk factor for CAD patients who underwent PCI[25-27]. RPP combines the information of HR and SBP, and both the indicators are related to the prognosis of CAD patients. Compared with SBP, DBP, MAP (average arterial pressure), HR and other indicators, RPP may have a higher predictive value[12].

At present, most studies mainly take RPP as one of the important evaluation indicators of disease-related rehabilitation training, which provides patients with reasonable exercise training guidelines for rehabilitation[28, 29]. Moore et al.[30] found that the overall risk of cancer mortality increased significantly in patients with no physical activity but with a higher RPP. In this study, we investigated the predictive value of the RPP on the clinical outcomes of CAD patients who underwent PCI.

There is a problem worth exploring. Tan Xu et al.[31] found that high RPP was associated with MACEs for ACS patients who underwent PCI. However, the predictive value of the RPP weakened when adjusting for HR. As a result, they have shown that RPP may reflect the predictive power of HR for ACS patients undergoing PCI, which was inconsistent with our study. This may be because the study involved a follow-up for of only two years, and the study population and results were different; in particular, cardiogenic shock patients and inpatients with MACEs in that study were excluded.

5. Conclusion

In summary, rate pressure product is an independent predictor of long-term prognosis in CAD patients who underwent PCI. A higher baseline RPP before PCI increased the risk of adverse outcomes. Therefore, the risk of disease can be stratified according to the RPP before PCI, which provides a basis for the treatment strategy of CAD patients, and a new concept for preventing the occurrence of clinical outcomes.

Acknowledgements: The authors are grateful to the Department of Cardiology at the First Affiliated Hospital of Xinjiang Medical University for their support and expertise in conducting this study.
Contributorship statement: Conceived and designed the research: Xiang Xie, Yi -Tong Ma. Performed the research: Zhi-hui Jiang, Analysed the data: Zhi-hui Jiang, Abudula Aierken, Ting-Ting Wu, Ying-Ying Zheng. Quality control of the study and revision: Xiang Xie, Yi -Tong Ma. Wrote the paper: Zhi-hui Jiang.
Competing interests: The authors declare that there are no conflicts of interest regarding the publication of this paper.
Funding: This work was funded by the National Natural Science Foundation of China (82170345).
Data sharing statement: The data used to support the findings of this research are available from the corresponding author upon request.
Patient and Public Involvement: No patient involved.

Reference

1. De Backer G, Ambrosioni E, Borch-Johnsen K, et al. European Society of Cardiology Committee for Practice Guidelines. European guidelines on cardiovascular disease prevention in clinical practice: third joint task force of European and other societies on cardiovascular disease prevention in clinical practice (constituted by representatives of eight societies and by invited experts). Eur J Cardiovasc Prev Rehabil. 2003 Aug;10(4):S1-S10.

For peer review only

2. Sia CH, Ko J, Zheng H, et al. Association between smoking status and outcomes in myocardial infarction patients undergoing percutaneous coronary intervention. Sci Rep. 2021 Mar 19;11(1):6466.
3. Kuehnemund L, Koeppe J, Feld J,et al. Gender differences in acute myocardial infarction-A nationwide German real-life analysis from 2014 to 2017. Clin Cardiol. 2021 Jul;44(7):890-898.
4. Xu JJ, Jiang L, Song Y, et al. [Related factors and the long-term outcome after percutaneous coronary intervention of premature acute myocardial infarction]. Zhonghua Xin Xue Guan Bing Za Zhi. 2020 Aug 24;48(8):655-660.
5. Malakar AK, Choudhury D, Halder B, et al. A review on coronary artery disease, its risk factors, and therapeutics. J Cell Physiol. 2019 Aug;234(10):16812-16823.
6. Nishihira K, Watanabe N, Kuriyama N, et al. Clinical outcomes of nonagenarians with acute myocardial infarction who undergo percutaneous coronary intervention. Eur Heart J Acute Cardiovasc Care. 2020 Aug;9(5):488-495.
7. Ahmad Y, Howard JP, Arnold A, et al. Complete Revascularization by Percutaneous Coronary Intervention for Patients With ST-Segment-Elevation Myocardial Infarction and Multivessel Coronary Artery Disease: An Updated Meta-Analysis of Randomized Trials. J Am Heart Assoc. 2020 Jun 16;9(12):e015263.
8. Oh YS, Lee HJ, Lee J, et al. Long-term clinical outcomes after a percutaneous coronary intervention with a drug-eluting stent in patients with unprotected left main coronary artery disease excluded from clinical trials. Coron Artery Dis. 2019 Jun;30(4):239-248.
9. Whitman M, Jenkins C, Sabapathy S, et al. Comparison of Heart Rate Blood Pressure Product Versus Age-Predicted Maximum Heart Rate as Predictors of Cardiovascular Events During Exercise Stress Echocardiography. Am J Cardiol. 2019 Aug 15;124(4):528-533.
10. Berényi I, Hajduczki IS, Böszörményi E. Quantitative evaluation of exercise-induced ST-segment depression for estimation of degree of coronary artery disease. Eur Heart J. 1984 Apr;5(4):289-94.
11. Cook CM, Ahmad Y, Howard JP, et al. Impact of Percutaneous Revascularization on Exercise Hemodynamics in Patients With Stable Coronary Disease. J Am Coll Cardiol. 2018 Aug 28;72(9):970-983.
12. Yazdani B, Kleber ME, Yücel G, et al. Association of double product and pulse pressure with cardiovascular and all-cause mortality in the LURIC study. J Clin Hypertens (Greenwich). 2020 Dec;22(12):2332-2342.
13. American Diabetes Association. 2. Classification and Diagnosis of Diabetes: Standards of Medical Care in Diabetes-2021. Diabetes Care. 2021 Jan;44(Suppl 1):S15-S33.
14. Unger T, Borghi C, Charchar F, et al. 2020 International Society of Hypertension Global Hypertension Practice Guidelines. Hypertension. 2020 Jun;75(6):1334-1357.
15. Park JY, Choi BG, Rha SW, et al. Five-year outcomes in patients with anemia on admission undergoing a coronary intervention for acute myocardial infarction in Koreans: propensity score matching analysis. Coron Artery Dis. 2018 Dec;29(8):647-651.
16. Gobel FL, Norstrom LA, Nelson RR, et al. The rate-pressure product as an index of myocardial oxygen consumption during exercise in patients with angina pectoris. Circulation. 1978 Mar;57(3):549-56.
17. Guzun R, Kaambre T, Bagur R, et al. Modular organization of cardiac energy metabolism: energy conversion, transfer and feedback regulation. Acta Physiol

For peer review only

(Oxf). 2015 Jan;213(1):84-106.
18. Hermida RC, Fernández JR, Ayala DE, et al. Circadian rhythm of double (rate-pressure) product in healthy normotensive young subjects. Chronobiol Int. 2001 May;18(3):475-89.
19. Stegehuis VE, Wijntjens GWM, Bax M, et al. Impact of clinical and haemodynamic factors on coronary flow reserve and invasive coronary flow capacity in non-obstructed coronary arteries: a patient-level pooled analysis of the DEBATE and ILIAS studies. EuroIntervention. 2021 Apr 2;16(18):e1503-e1510.
20. Fox K, Borer JS, Camm AJ, et al. Resting heart rate in cardiovascular disease. J Am Coll Cardiol. 2007 Aug 28;50(9):823-30.
21. Jensen MT, Kaiser C, Sandsten KE, et al. Heart rate at discharge and long-term prognosis following percutaneous coronary intervention in stable and acute coronary syndromes--results from the BASKET PROVE trial. Int J Cardiol. 2013 Oct 9;168(4):3802-6.
22. Zheng YY, Wu TT, Chen Y, et al. Resting Heart Rate and Long-Term Outcomes in Patients with Percutaneous Coronary Intervention: Results from a 10-Year Follow-Up of the CORFCHD-PCI Study. Cardiol Res Pract. 2019 Apr 1;2019:5432076.
23. Fox K, Ford I, Steg PG, et al. Heart rate as a prognostic risk factor in patients with coronary artery disease and left-ventricular systolic dysfunction (BEAUTIFUL): a subgroup analysis of a randomised controlled trial. Lancet. 2008 Sep 6;372(9641):817-21.
24. O'Brien J, Reid CM, Andrianopoulos N, et al. Heart Rate as a Predictor of Outcome Following Percutaneous Coronary Intervention. Am J Cardiol. 2018 Oct 1;122(7):1113-1120.
25. Warren J, Nanayakkara S, Andrianopoulos N, et al. Impact of PreProcedural Blood Pressure on Long-Term Outcomes Following Percutaneous Coronary Intervention. J Am Coll Cardiol. 2019 Jun 11;73(22):2846-2855.
26. Kawashiri MA, Sakata K, Hayashi K, et al. Impact of combined lipid lowering and blood pressure control on coronary plaque: myocardial ischemia treated by percutaneous coronary intervention and plaque regression by lipid lowering and blood pressure controlling assessed by intravascular ultrasonography (MILLION) study. Heart Vessels. 2017 May;32(5):539-548.
27. Shiraishi J, Kohno Y, Sawada T, et al. Prognostic impact of systolic blood pressure at admission on in-hospital outcome after primary percutaneous coronary intervention for acute myocardial infarction. J Cardiol. 2012 Aug;60(2):139-44.
28. Carter SJ, Hunter GR, McAuley E, et al. Lower rate-pressure product during submaximal walking: a link to fatigue improvement following a physical activity intervention among breast cancer survivors. J Cancer Surviv. 2016 Oct;10(5):927-34.
29. Brown KD, Nguyen HL, Adams J. Continuous blood pressure monitoring during high-intensity resistance training after myocardial infarction or percutaneous coronary intervention in a phase 2 cardiac rehabilitation setting. Proc (Bayl Univ Med Cent). 2020 Apr 3;33(3):342-345.
30. Moore JX, Carter SJ, Williams V, et al. Physical health composite and risk of cancer mortality in the REasons for Geographic and Racial Differences in Stroke Study. Prev Med. 2020 Mar;132:105989.
31. Xu T, Zhan Y, Lu N, et al. Double product reflects the association of heart rate with MACEs in acute coronary syndrome patients treated with percutaneous coronary intervention. BMC Cardiovasc Disord. 2017 Dec 2;17(1):284.

For peer review only

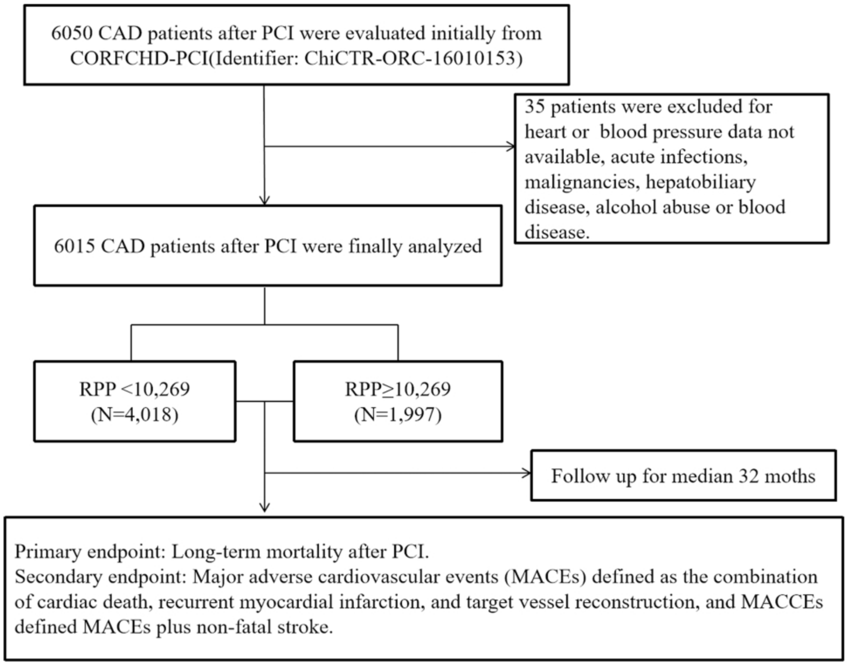

Figure 1 The participant flow chart

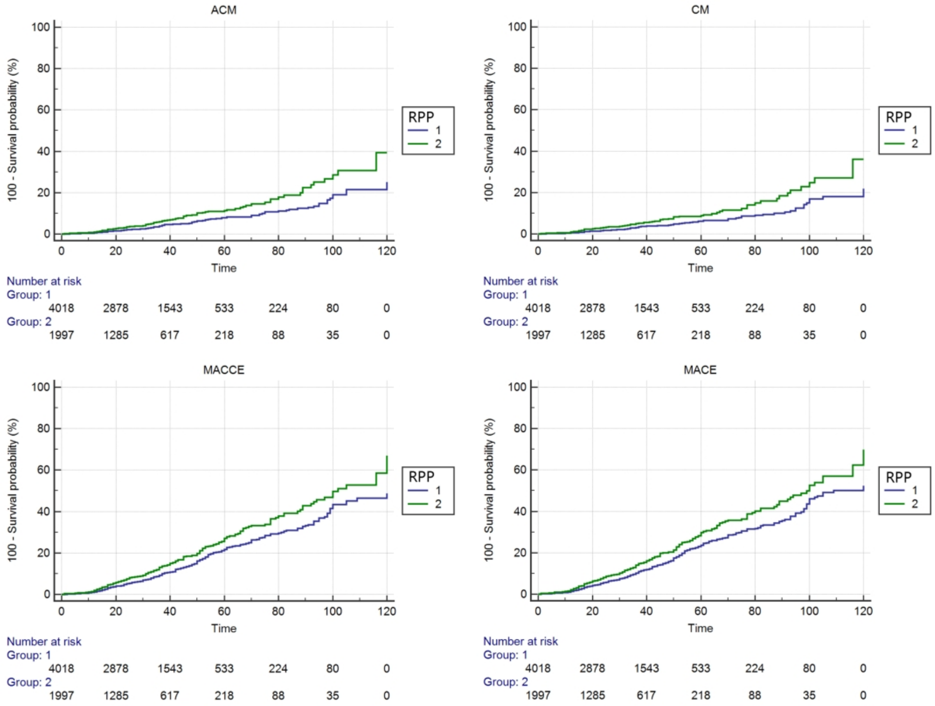

Figure 2 Cumulative Kaplan-Meier estimates of the time to the first adjudicated occurrence of primary endpoints and secondary endpoints

Rate pressure product as a novel predictor of long-term adverse outcomes in patients after percutaneous coronary intervention: A retrospective cohort study

Journal:	*BMJ Open*
Manuscript ID	bmjopen-2022-067951.R1
Article Type:	Original research
Date Submitted by the Author:	20-Jan-2023

Complete List of Authors:	Jiang, Zhi-Hui; Xinjiang Medical University Affiliated First Hospital, Department of Cardiology<br>Aierken, Abudula; Xinjiang Medical University Affiliated First Hospital<br>Wu, Ting-Ting; Xinjiang Medical University Affiliated First Hospital<br>Zheng, Ying-Ying; Xinjiang Medical University Affiliated First Hospital<br>Ma, Yi-Tong ; Xinjiang Medical University Affiliated First Hospital<br>Xie, Xiang; Xinjiang Medical University Affiliated First Hospital, Department of Cardiology

**Primary Subject Heading**:	Cardiovascular medicine
Secondary Subject Heading:	Cardiovascular medicine, Geriatric medicine, Medical management
Keywords:	Coronary heart disease < CARDIOLOGY, Coronary intervention < CARDIOLOGY, Cardiology < INTERNAL MEDICINE, Myocardial infarction < CARDIOLOGY, GERIATRIC MEDICINE

SCHOLARONE™
Manuscripts

I, the Submitting Author has the right to grant and does grant on behalf of all authors of the Work (as defined in the below author licence), an exclusive licence and/or a non-exclusive licence for contributions from authors who are: i) UK Crown employees; ii) where BMJ has agreed a CC-BY licence shall apply, and/or iii) in accordance with the terms applicable for US Federal Government officers or employees acting as part of their official duties; on a worldwide, perpetual, irrevocable, royalty-free basis to BMJ Publishing Group Ltd ("BMJ") its licensees and where the relevant Journal is co-owned by BMJ to the co-owners of the Journal, to publish the Work in this journal and any other BMJ products and to exploit all rights, as set out in our licence.

The Submitting Author accepts and understands that any supply made under these terms is made by BMJ to the Submitting Author unless you are acting as an employee on behalf of your employer or a postgraduate student of an affiliated institution which is paying any applicable article publishing charge ("APC") for Open Access articles. Where the Submitting Author wishes to make the Work available on an Open Access basis (and intends to pay the relevant APC), the terms of reuse of such Open Access shall be governed by a Creative Commons licence – details of these licences and which Creative Commons licence will apply to this Work are set out in our licence referred to above.

Other than as permitted in any relevant BMJ Author's Self Archiving Policies, I confirm this Work has not been accepted for publication elsewhere, is not being considered for publication elsewhere and does not duplicate material already published. I confirm all authors consent to publication of this Work and authorise the granting of this licence.

For peer review only

Rate pressure product as a novel predictor of long-term adverse outcomes in patients after percutaneous coronary intervention: A retrospective cohort study

Zhi-Hui Jiang, Abudula Aierken, Ting-Ting Wu, Ying-Ying Zheng, Yi-Tong Ma, Xiang Xie*

* Correspondence: xiangxie999@sina.com

Department of Cardiology, First Affiliated Hospital of Xinjiang Medical University, Urumqi 830011, People's Republic of China

Abstract

Objectives:

Previous studies have suggested that heart rate (HR) and blood pressure (BP) play important roles in the development of adverse outcomes in patients with coronary artery disease (CAD) who underwent percutaneous coronary intervention (PCI). However, the relationship between the rate pressure product (RPP) and long-term outcomes has rarely been investigated. This study investigated the effects of RPP on the clinical outcomes of CAD patients who underwent PCI.

Methods:

In this study, a total of 6,015 CAD patients were enrolled. All patients were from the CORFCHD-PCI study. They were divided into two groups according to RPP (RPP<10,269, *n*=4,018 and RPP≥10,269, *n*=1,997). In addition, the median follow-up time was 32 months. The primary endpoint was long-term mortality, including all-cause mortality (ACM) and cardiac mortality (CM). The secondary endpoints were major adverse cardiovascular events (MACEs) and major adverse cardiovascular and cerebrovascular events (MACCEs).

Results:

We found that there were significant differences between the two groups in the incidences of ACM, CM, MACCEs and MACEs (all $P<0.05$). Among the CAD patients with ACM, CM, MACCEs and MACEs, the mean survival time of the low-value group was significantly higher than that of the high-value group. Multivariate Cox regression analyses showed that RPP was an independent predictor for ACM (hazard ratio (*HR*)=1.605, 95% confidence interval (*CI*): 1.215-2.120, *P*=0.001), CM (*HR*=1.733, 95% *CI*: 1.267-2.369, *P*=0.001), MACCEs (*HR*=1.271, 95% *CI*: 1.063-1.518, *P*=0.008) and MACEs (*HR*=1.315, 95% *CI*: 1.092-1.584, *P*=0.004) in stable CAD patients. While there was no significant correlation between the RPP and the adverse outcomes in acute coronary syndrome (ACS) patients.

Conclusion:

In summary, RPP is an independent predictor of long-term prognosis in CAD patients who underwent PCI. A higher baseline RPP before PCI increased the risk of adverse outcomes. Compared with heart rate and blood pressure alone, RPP has a higher predictive value for adverse clinical outcomes.

Keywords: Coronary artery disease, Rate pressure product, Mortality, Outcomes, Percutaneous coronary intervention

Strengths and limitations of this study

a) The present study was a single-centre retrospective cohort study with a larger sample number and a long follow-up time, which can improve the scientific nature

For peer review only

of the results.

b) The present study only collected heart rate and blood pressure data at the first hospitalization, thus lacking information on dynamic changes in these variables.
c) The present study did not rule out the effect of the patient's current medication such as antihypertensive drugs on blood pressure levels.
d) Follow-up was mainly based on telephone contacts and the method of measurement was mainly based on manual, which may have impacted the results.

1 Introduction

Coronary atherosclerotic heart disease refers to the occurrence of atherosclerosis in the coronary arteries, which narrows or obstructs the arteries, causing local myocardial ischemia, hypoxia, and even necrosis, resulting in changes in heart structure and loss of function. Together with coronary artery spasms are collectively called coronary artery disease (CAD)[1]. CAD is one of the main causes of death and disability in China, and is a serious threat to health. The WHO classifies CAD into 5 clinical types: silent myocardial ischemia (latent coronary artery disease), angina pectoris, myocardial infarction, ischemia heart failure (ischemic heart disease) and sudden cardiac death. The related risk factors include sex, age, smoking, insufficient exercise, obesity, diabetes, dyslipidemia and metabolic disorders, hypertension, family history of CAD and others[2-5]. At present, percutaneous coronary intervention (PCI) is one of the most important methods for the treatment of coronary artery disease, and can significantly reduce the mortality and risk of reischemic attacks[6-8]. As an established measure of myocardial load, rate pressure product (RPP) may provide a more reliable assessment of cardiac workload[9]. Previous studies have shown that RPP can be used as an auxiliary reference index for coronary artery disease, which is negatively correlated with the degree of coronary artery disease and the number of diseased branches and negatively correlated with age and sex[10]. RPP indirectly reflects myocardial blood supply and oxygen consumption. Cook et al. [11] found that PCI can increase myocardial workload. The change observed immediately following PCI was caused by the abolition of stenosis resistance. Yazdani et al.[12] found that compared with systolic blood pressure (SBP), diastolic blood pressure (DBP), and HR, RPP was a stronger predictor of all-cause mortality (ACM) and cardiac mortality (CM) in the general population and in patients with coronary artery disease, especially in the cohort of patients at high risk of cardiovascular disease. Interestingly, RPP also predicted cardiac mortality in heart failure with preserved ejection fraction (HFrEF) patients in a stronger way, while SBP and HR alone showed no significant association. Furthermore, in 3-vessel CAD patients RPP was a stronger predictor of cardiac mortality than SBP or HR alone. Therefore, especially in patients with severe coronary artery disease and heart failure, the cardiac workload given as RPP should be considered in clinical practice[12]. However, they did not clarify the predictability of RPP for the prognosis of CAD patients who underwent PCI. The RPP was defined as the product of SBP (mmHg) and HR (beats per minute), because it combined the information of the two indicators, and both indicators were related to the prognosis of CAD patients. Therefore, we speculated that RPP may affect the long-term prognosis of CAD patients after PCI. Compared with HR and SBP, RPP may have a higher predictive value for adverse clinical outcomes.

In this research, 6,015 CAD patients were included to investigate the relationship between the RPP and the clinical outcomes.

2 Methods

2.1 Study design and population

For peer review only - http://bmjopen.bmj.com/site/about/guidelines.xhtml

In this study, 6,050 CAD patients who were hospitalized at the First Affiliated Hospital of Xinjiang Medical University from January 2008 to December 2016 were evaluated. Regarding the inclusion criteria, coronary angiography confirmed that at least one major coronary artery showed stenosis ≥70%, including the left main coronary artery, left anterior descending artery, left circumflex artery, and right coronary artery, or other more important branches. All patients underwent PCI and received at least one stent via implantation. Regarding the exclusion criteria, patients with the presence of serious heart failure, rheumatic heart disease, valvular heart disease, congenital heart disease, pulmonary heart disease, serious dysfunction of the liver or kidney, acute infections, malignant tumor, alcohol abuse or blood disease were excluded. To investigate the relation between the RPP and the outcomes in CAD patients who underwent PCI, a total of 6,050 patients were evaluated initially according to the inclusion and exclusion criteria. Thirty-five patients were excluded due to incomplete follow-up data. Finally, 6,015 patients were enrolled in this study, including 2,029 ACS patients and 3,986 stable CAD patients. All CAD patients were from the Clinical Outcomes and Risk Factors of Patients with Coronary Heart Disease after PCI (CORFCHD-PCI) study. The study is a large single-centre retrospective cohort study based on medical records and the follow-up information collected at the First Affiliated Hospital of Xinjiang Medical University. The CORFCHD-PCI study complied with the Declaration of Helsinki and the protocol was approved by the Ethics Committee of the First Affiliated Hospital of Xinjiang Medical University. The details of the design have been registered at http://www.chictr.org.cn (identifier: ChiCTR-ORC-16010153). Since the study was designed retrospectively, the Ethics Committee considered that it was not necessary to obtain informed consent form the eligible patients. **Figure 1** shows the flow chart of the inclusion and exclusion criteria for the selection of 6,050 CAD patients after PCI.

Patient and Public Involvement: No patient involved.

2.2 Data collection

The cardiovascular-related risk factors, clinical data, laboratory data and interventional therapy data of all patients were collected retrospectively, including: age, sex, heart rate, blood pressure, smoking status, alcohol consumption, history of diagnosed diabetes, history of hypertension, family history of CAD, history of medication, blood urea nitrogen (BUN), serum creatinine (Cr), uric acid (UA), glucose (GLU), triglycerides (TG), total cholesterol (TC), low density lipoprotein cholesterol (LDL-C), high-density lipoprotein cholesterol (HDL-C), and left ventricular ejection fractions (LVEF). HR and BP at admission were measured accurately according to a highly standardized protocol at the first medical contact by experienced physicians using calibrated manual mercury sphygmomanometers with an appropriately sized cuff. One sphygmomanometer was placed on the right arm, and after at least 5 min of rest in the sitting position. Two HR and BP measurements were obtained and taking the average, allowing for a 1 min interval between measurements. All laboratory tests were carried out in the Medical Laboratory Centre of the First Affiliated Hospital of Xinjiang Medical University.

2.3 Clinical diagnosis

The diagnostic criteria for diabetes mellitus were a clear history of diabetes, the use of hypoglycemic agents, fasting plasma glucose ≥7.0 mmol/L, 2-h plasma glucose ≥11.1 mmol/L during oral glucose tolerance test (OGTT), A1C ≥ 6.5%(48 mmol/mol), or a random plasma glucose ≥11.1 mmol/L in a patient with classic symptoms of hyperglycemia or hyperglycemic crisis[13]. Hypertension was diagnosed when SBP was ≥140 mmHg and/or DBP was ≥ 90 mmHg following repeated examination, or treatment with antihypertensive drugs[14].

For peer review only

2.4 Endpoints

The primary endpoint was long-term mortality, including long-term all-cause mortality (ACM) and cardiac mortality (CM). The secondary endpoints were major adverse cardiovascular events (MACEs) and major adverse cardiovascular and cerebrovascular events (MACCEs). MACEs referred to the composite endpoint of cardiac death, recurrent myocardial infarction and target vessel reconstruction. MACCEs was defined as MACEs plus nonfatal stroke[15].

2.5 Follow-Up

In this study, 6,050 CAD patients started receiving follow-up when they underwent percutaneous coronary intervention, and regular follow-up was performed at the end of 1 month, 3 months, 6 months, 1 year, 3 years, and 5 years after discharge. Outpatient review and telephone were utilized to follow-up until the end point events occurred or the study was concluded. The median follow-up time was 32 months, and the longest follow-up time was 10 years.

2.6 Statistical analyses

All statistical analyses were performed using SPSS version 26.0 (SPSS Inc, Chicago, Illinois, United States). Continuous data are presented as the mean ± standard deviation ($\bar{x} \pm s$), and categorical variables are presented as frequencies and percentages. RPP is a continuous numerical variable. Because there is no established optimal threshold for RPP, receiver operating characteristic (ROC) curve analysis was performed to assess the predictive ability of RPP in clinical outcomes. The point where the sensitivity and specificity were maximized was determined as the best cut-off point. Two samples *t*-tests or chi-square tests were used to compare the clinical data of patients in different groups and the characteristics of adverse outcomes. Kaplan-Meier analysis was used to compare the cumulative incidence rates of long-term adverse outcomes and the log-rank test was used to compare between groups. Multivariate Cox regression analysis was performed to evaluate the predictive value of RPP for clinical outcomes. The fully adjusted models for RPP included age, hypertension, diabetes mellitus, sex, LDL-C, TC, smoking, alcohol consumption. Hazard ratios (*HRs*) and 95% confidence intervals (*CIs*) were also calculated. A *P*-Value of < 0.05 was considered statistically significant.

3 Results

3.1 Baseline data

Among the 6,015 patients included in this study, 74.3% were males, 40.0% were smokers, 29.3% were drinkers, 24.1% had diabetes mellitus, 42.3% had hypertension, and the mean age of the patients was 59.48 years. During the follow-up period, a history of using calcium antagonists accounted for 11.5%, and patients using β-receptor blockers, ACEI/ARB drugs and statins accounted for 40.4%, 22.8% and 54.4%, respectively. During the 10-year follow-up, 309 (5.1%) deaths occurred, of which 251 (4.2%) patients died due to cardiovascular disease. MACCEs occurred in 859 (14.3%) patients, including 782 (13.0%) MACEs and 77 (1.28%) nonfatal strokes.

In this study, the ROC curve analysis showed that a RPP value of 10,269 was the cut-off with the highest sensitivity and specificity in terms of prognostic significance. The population clinical characteristics were grouped by dichotomy of RPP, which was based on the ROC cut-off. RPP<10,269 was considered the low-value group, and RPP≥10,269 was considered the high-value group. There were 4,018 CAD patients in the low-value group and 1,997 CAD patients in the high-value group. As shown in **Table 1**, we found that there were significant differences in many variables between the two groups (all $P<0.05$), specifically, age, BUN, GLU, TC, LDL-C, diabetes

For peer review only - http://bmjopen.bmj.com/site/about/guidelines.xhtml

mellitus and hypertension in the high-value group were higher than those in the low-value group. Sex, smoking, alcohol consumption and the number of statins used in the high-value group were lower than those in the low-value group. We did not find statistically significant differences between the two groups in terms of Cr, UA, TG, HDL-C, LVEF, or the therapy of CCB drugs, β-receptor blocker drugs or ACBI/ARB drugs (all P>0.05). In the ACS patients, we found that diabetes mellitus, hypertension, GLU, TG and TC were significantly different between the two groups. In the stable CAD patients, we found that age, sex, smoking, alcohol drinking, diabetes, hypertension, BUN, UA and GLU were significantly different between the two groups (all P<0.05). As shown in **Table 2**, several variables were significantly different between these two groups, especially in the ACS patients. These variables included post-dilatation pressure, chronic total occlusion lesions (CTO), multi-vessel lesions (ML) as well as the number of vascular lesions (all P<0.05).

3.2 Clinical outcomes

A comparison of clinical outcomes between the two groups of CAD patients is shown in **Table 3**. For the primary endpoints, the incidence rate of all-cause death was 6.4% in the high-value group and 4.5% in the low-value group. The difference wea significant (P=0.002); also, the incidence rate of cardiac death between the two groups showed a significant difference (5.3% vs. 3.6%, P=0.002). For the secondary endpoints, we found that there were significant differences between the two groups in the incidence of MACCEs (15.8% vs. 13.5%, P=0.020) and MACEs (14.4% vs. 12.3%, P=0.020).

Sub-group analysis suggested that there were significant differences in the incidence of ACM (6.9% vs. 4.8%, P=0.004), CM (5.7% vs. 3.7%, P=0.003) and MACEs (14.4% vs. 12.2%, P=0.040) between the high-value group and the low-value group for the stable CAD patients. For the ACS patients, we found no significant difference in adverse outcomes (all P>0.05).

For peer review only

Table 1 Characteristics of participants of the two groups

Variables	Total				ACS				Stable CAD			
	RPP<10,269 (*n*=4,018)	RPP≥10,269 (*n*=1,997)	x^2or *t*	*P* value	RPP<10,269 (*n*=1,444)	RPP≥10,269 (*n*=585)	x^2or *t*	*P* value	RPP<10,269 (*n*=2,574)	RPP≥10,269 (*n*=1,412)	x^2or *t*	*P* value
Age, years	58.92±10.90	60.62±10.61	-5.751	**<0.001**	59.57±10.89	60.60±11.10	-1.930	0.054	56.56±10.89	60.63±10.40	-0.835	**<0.001**
Male, n (%)	3057(76.1)	1415(70.9)	19.105	**<0.001**	358(24.8)	169(28.9)	3.634	0.057	603(23.4)	413(29.2)	16.277	**<0.001**
Smoking, n (%)	1686(42.0)	718(36.0)	20.064	**<0.001**	607(42.0)	228(39.0)	1.611	0.204	1079(41.9)	490(34.7)	19.895	**<0.001**
Alcohol drinking, n (%)	1217(30.3)	544(27.2)	5.985	**0.014**	434(30.1)	167(28.5)	0.454	0.500	783(30.4)	377(26.7)	6.115	**0.013**
Diabetes, n (%)	847(21.1)	600(30.0)	58.684	**<0.001**	301(20.8)	177(30.3)	20.478	**<0.001**	546(21.2)	423(30.0)	37.900	**<0.001**
Hypertension, n (%)	1478(36.8)	1067(53.4)	151.429	**<0.001**	555(38.4)	309(52.8)	35.239	**<0.001**	923(35.9)	758(53.7)	118.784	**<0.001**
BUN, mmol/L	5.48±1.65	5.59±1.72	-2.271	**0.023**	5.50±1.71	5.56±1.84	-0.695	0.487	5.48±1.61	5.60±1.67	-2.306	**0.021**
Cr, umol/L	75.78±19.55	76.03±21.97	-0.447	0.655	76.00±20.77	77.59±21.79	-1.510	0.131	75.65±18.84	75.40±22.02	0.382	0.703
UA, mmol/L	323.96±89.56	322.21±91.03	0.697	0.486	320.62±89.64	328.53±92.28	-1.750	0.080	325.83±89.49	319.64±90.43	2.051	**0.040**
GLU, mmol/L	6.41±2.99	6.92±3.39	-5.888	**<0.001**	6.41±3.02	7.22±3.39	-5.209	**<0.001**	6.41±2.97	6.80±3.39	-3.706	**<0.001**
TG, mmol/L	1.88±1.23	1.94±1.35	-1.751	0.080	1.88±1.25	2.04±1.39	-2.531	**0.011**	1.88±1.22	1.90±1.33	-0.470	0.638
TC, mmol/L	3.94±1.10	4.01±1.13	-2.305	**0.021**	3.92±1.07	4.04±1.10	-2.089	**0.037**	3.95±1.11	4.00±1.14	-1.371	0.170
LDL-C, mmol/L	2.44±0.90	2.51±0.95	-2.689	**0.007**	2.41±0.87	2.50±0.96	-1.908	0.057	2.45±0.91	2.51±0.95	-1.866	0.062
HDL-C, mmol/L	1.02±0.46	1.02±0.52	-0.196	0.845	1.01±0.44	1.01±0.46	0.288	0.774	1.02±0.48	1.03±0.54	-0.361	0.718
CCB, n (%)	462(11.6)	227(11.4)	0.022	0.883	184(12.8)	67(11.5)	0.644	0.422	278(10.9)	160(11.4)	0.270	0.603
β-blockers, n (%)	1631(40.8)	785(39.5)	0.930	0.335	580(40.4)	234(40.2)	0.004	0.948	1051(41.0)	551(39.2)	1.268	0.260
ACEI or ARB, n (%)	894(22.4)	472(23.8)	1.525	0.217	325(22.6)	142(24.4)	0.760	0.383	569(22.2)	330(23.5)	0.878	0.349
Statins, n (%)	2207(55.5)	1035(52.2)	5.480	**0.019**	797(55.8)	303(52.2)	2.123	0.145	1410(55.3)	732(52.2)	3.284	0.070
LVEF, n (%)	61.10±7.09	61.00±6.97	0.454	0.650	61.09±6.88	60.84±7.01	0.707	0.479	61.10±7.21	61.07±6.96	0.111	0.911

Note: *BUN, blood urea nitrogen; UA, uric acid; Cr, creatinine; GLU, glucose; TG, triglyceride; TC, total cholesterol; LDL-C, low density lipoprotein cholesterol; HDL-C, high density lipoprotein cholesterol; LVEF, left ventricular ejection fractions.*

For peer review only

Table 2 Baseline treatments and procedure characteristics

Variables	Total				ACS				Stable CAD			
	RPP<10,269 (*n*=4,018)	RPP≥10,269 (*n*=1,997)	x^2 or *t*	*P* value	RPP<10,269 (*n*=1,444)	RPP≥10,269 (*n*=585)	x^2 or *t*	*P* value	RPP<10,269 (*n*=2,574)	RPP≥10,269 (*n*=1,412)	x^2 or *t*	*P* value
New generation stent, *n* (%)	3793(94.4)	1875(93.9)	0.699	0.403	1369(94.9)	553(94.5)	0.098	0.754	2424(94.2)	1322(93.6)	0.481	0.488
Diameter of stents, mm	2.85±0.37	2.85±0.37	-0.676	0.499	2.87±0.38	2.85±0.87	0.858	0.391	2.84±0.37	2.86±0.38	-1.548	0.122
Length of stents, mm	28.05±6.96	27.88±6.97	0.843	0.399	28.11±6.96	27.51±6.89	1.778	0.075	28.01±6.96	28.04±7.01	-0.143	0.886
No. of stents, *n*	1.04±0.22	1.04±0.24	-0.858	0.391	1.04±0.22	1.07±0.28	-1.901	0.057	1.04±0.22	1.04±0.22	0.193	0.847
Pre-dilatation, *n* (%)	3476(86.5)	1729(86.6)	0.003	0.959	1259(87.2)	505(86.3)	0.314	0.575	2217(86.1)	1224(86.7)	0.238	0.626
Post-dilatation, *n* (%)	2542(63.3)	1217(60.9)	3.115	0.078	913(63.3)	355(60.7)	1.189	0.276	1629(63.3)	862(61.0)	1.949	0.163
Post-dilatation pressure, atm	13.53±3.59	14.61±3.31	-9.342	**<0.001**	13.41±3.33	14.41±3.45	-5.105	**<0.001**	13.60±3.72	14.68±3.24	-7.658	**<0.001**
CTO, *n* (%)	873(21.7)	529(26.5)	16.885	**<0.001**	284(19.7)	201(34.4)	49.284	**<0.001**	589(22.9)	328(23.2)	0.062	0.804
ML, *n* (%)	2573(64.1)	1333(66.8)	4.263	**0.039**	972(67.4)	428(73.2)	6.556	**0.010**	1601(62.2)	905(64.1)	1.402	0.236
No. of vascular lesions, *n*	1.99±0.84	2.05±0.85	-2.617	**0.009**	2.06±0.84	2.18±0.83	-2.928	**0.003**	1.96±0.84	2.00±0.85	-1.606	0.108

Note: *CTO, chronic total occlusion lesions; RPP, rate pressure product; ML, multi-vessel lesions.*

Table 3 Outcomes comparison between groups

Outcomes	Total				ACS				Stable CAD			
	RPP<10,269 (*n*=4,018)	RPP≥10,269 (*n*=1,997)	x^2	*P* value	RPP<10,269 (*n*=1,444)	RPP≥10,269 (*n*=585)	x^2	*P* value	RPP<10,269 (*n*=2,574)	RPP≥10,269 (*n*=1,412)	x^2	*P* value
ACM, n (%)	181(4.5)	128(6.4)	9.933	**0.002**	58(4.0)	30(5.1)	1.240	0.265	123(4.8)	98(6.9)	8.138	**0.004**
CM, n (%)	145 (3.6)	106(5.3)	9.632	**0.002**	49(3.4)	25(4.3)	0.918	0.338	96(3.7)	81(5.7)	8.655	**0.003**
MACCE, n (%)	544 (13.5)	315(15.8)	5.441	**0.020**	198(13.7)	94(16.1)	1.876	0.171	346(13.4)	221(15.7)	3.648	0.056
MACE, n (%)	494 (12.3)	288(14.4)	5.336	**0.021**	181(12.5)	84(14.4)	1.220	0.269	313(12.2)	204(14.4)	4.227	**0.040**

Note: *ACM, all-cause mortality; CM, cardiac mortality; MACE, major adverse cardiovascular events; MACCE, major adverse cardiovascular and cerebrovascular events.*

For peer review only

In this study, baseline RPP before PCI was used as an independent variable, and ACM, CM, MACEs and MACCEs were used as the endpoints. Kaplan-Meier analysis was performed, and the log-rank test was used for comparisons between groups. The results are shown in **Table 4**. Among the CAD patients with ACM, CM, MACCEs and MACEs, the mean survival time of the low-value group was significantly higher than that of the high-value group. As shown in **Figure 2**, with the extension of the follow-up time, the cumulative survival rate of the patients showed a downward trend, and the decreasing rate of the high-value group was more significant than that of the low-value group. While the cumulative risk showed an upward trend, the increasing rate of the high-value group was significantly higher than that of the low-value group. The above differences were statistically significant (all $P<0.05$).

Tables 5-8 show select variables based on the aforementioned analysis. The results of collinearity analysis showed that there was no significant collinearity among age, hypertension, diabetes mellitus, sex, GLU, BUN, LDL-C, TC, smoking, alcohol consumption, post-dilatation, the number of vascular lesions, CTO, ML and RPP. Multivariate Cox regression analysis was performed to assess the prognostic value of the RPP and adverse outcomes after adjusting for the influence of age, hypertension, diabetes mellitus, sex, GLU, BUN, LDL-C, TC, smoking, alcohol consumption and other confounding factors. In total, the results showed that higher preoperative RPP value was a risk factor for clinical outcomes in CAD patients who underwent PCI. The respective risks of ACM (HR=1.598, 95% CI: 1.262-2.024, $P<0.001$), CM (HR=1.678, 95% CI: 1.289-2.184, $P<0.001$), MACCEs (HR=1.263, 95% CI: 1.092-1.462, P=0.002) and MACEs (HR=1.281, 95% CI: 1.099-1.492, P=0.001) that occurred in the high-value group were still significantly higher than those in the low value group. In the stable CAD patients, after performing an adjustment of confounders, the ACM, CM, MACCEs and MACEs remained significantly different. While in the ACS group, there was no significant difference in adverse outcomes after adjusting for confounders.

Taking cardiac death as an example. **Figure 3** shows the ROC curves of RPP as well as HR and SBP for CAD patients with cardiac death. The results of the ROC curve analysis for different parameters are given in **Table 9**. The AUC of RPP (AUC 0.586) was larger compared to HR (AUC 0.554) or SBP (AUC 0.511).

For peer review only

Table 4 Mean Survival Time for Outcomes comparison between groups.

Outcomes	Mean Survival Time(m)		
	RPP <10,269 (*n*=4,018)	RPP≥10,269 (*n*=1,997)	Overall
ACM	109.512	103.379	107.681
CM	111.362	105.887	109.731
MACCE	92.150	85.849	90.204
MACE	94.267	88.089	92.375

Note: *ACM, all-cause mortality; CM, cardiac mortality; MACE, major adverse cardiovascular events; MACCE, major adverse cardiovascular and cerebrovascular events.*

Table 5 Multivariable Cox regression analysis for ACM

Variables	Total					ACS					Stable CAD				
	B	SE	Wald	*P* values	*HR* (95% *CI*)	B	SE	Wald	*P* values	*HR* (95% *CI*)	B	SE	Wald	*P* values	*HR* (95% *CI*)
Age	0.026	0.006	19.314	**<0.001**	1.027(1.015-1.039)	0.027	0.011	5.473	**0.019**	1.027(1.004-1.050)	0.027	0.007	14.141	**<0.001**	1.027(1.013-1.041)
Male	-0.010	0.149	0.005	0.944	0.990(0.739-1.325)	-0.091	0.295	0.095	0.758	0.913(0.513-1.627)	<0.001	0.173	<0.001	0.998	1.000(0.713-1.404)
Smoking	-0.035	0.153	0.053	0.817	0.965(0.715-1.303)	0.077	0.294	0.069	0.793	1.080(0.607-1.922)	-0.030	0.182	0.028	0.867	0.970(0.679-1.386)
Alcohol drinking	-0.017	0.162	0.011	0.915	0.983(0.715-1.351)	0.124	0.301	0.169	0.681	1.132(0.627-2.044)	-0.056	0.196	0.082	0.774	0.945(0.645-1.387)
Diabetes	0.032	0.149	0.047	0.828	1.033(0.772-1.382)	0.079	0.275	0.083	0.773	1.082(0.631-1.855)	-0.034	0.178	0.037	0.848	0.966(0.681-1.371)
Hypertension	0.186	0.123	2.282	0.131	1.205(0.946-1.534)	0.286	0.237	1.458	0.227	1.331(0.837-2.116)	0.098	0.146	0.448	0.503	1.103(0.828-1.470)
GLU	-0.012	0.020	0.361	0.548	0.988(0.949-1.028)	-0.012	0.039	0.099	0.753	0.988(0.915-1.066)	-0.016	0.024	0.461	0.497	0.984(0.939-1.031)
TC	0.088	0.087	1.027	0.311	1.092(0.921-1.296)	-0.177	0.159	1.231	0.267	0.838(0.613-1.145)	0.175	0.092	3.606	0.058	1.191(0.994-1.427)
BUN	0.079	0.032	6.088	**0.014**	1.082(1.016-1.153)	0.051	0.061	0.714	0.398	1.053(0.935-1.185)	0.09	0.038	5.459	0.019	1.094(1.015-1.180)
LDL-C	-0.172	0.109	2.480	0.115	0.842(0.679-1.043)	0.116	0.186	0.389	0.533	1.123(0.779-1.619)	-0.262	0.121	4.717	**0.030**	0.769(0.607-0.975)
Post-dilatation	0.132	0.124	1.140	0.286	1.141(0.896-1.454)	-0.085	0.231	0.135	0.713	0.919(0.584-1.444)	0.257	0.148	3.005	0.083	1.293(0.967-1.728)
No. of vascular lesions	0.180	0.143	1.575	0.209	1.197(0.904-1.584)	0.287	0.273	1.111	0.292	1.333(0.781-2.275)	0.190	0.170	1.243	0.265	1.209(0.866-1.687)
CTO	0.409	0.134	9.272	**0.002**	1.505(1.157-1.957)	0.600	0.253	5.609	**0.018**	1.823(1.109-2.995)	0.263	0.161	2.681	0.102	1.301(0.949-1.783)
ML	0.032	0.262	0.015	0.903	1.032(0.618-1.724)	-0.002	0.515	<0.001	0.997	0.998(0.363-2.741)	0.038	0.306	0.016	0.900	1.039(0.570-1.894)
RPP	0.469	0.121	15.098	**<0.001**	1.598(1.262-2.024)	0.249	0.242	1.058	0.304	1.283(0.798-2.064)	0.473	0.142	11.09	**0.001**	1.605(1.215-2.120)

Note: *GLU, glucose; TC, total cholesterol; BUN, blood urea nitrogen; LDL-C, low density lipoprotein cholesterol; CTO, chronic total occlusion lesions; ML, multi-vessel lesions; RPP, rate pressure product.*

For peer review only

Table 6 Multivariable Cox regression analysis for CM

Variables	Total					ACS					Stable CAD				
	B	SE	Wald	*P* values	*HR* (95% *CI*)	B	SE	Wald	*P* values	*HR* (95% *CI*)	B	SE	Wald	*P* values	*HR* (95% *CI*)
Age	0.018	0.007	7.267	**0.007**	1.018(1.005-1.031)	0.020	0.013	2.456	0.117	1.020(0.995-1.045)	0.017	0.008	4.805	**0.028**	1.017(1.002-1.033)
Male	-0.013	0.166	0.006	0.937	0.987(0.712-1.368)	-0.243	0.324	0.561	0.454	0.784(0.415-1.481)	0.065	0.195	0.110	0.740	1.067(0.728-1.564)
Smoking	-0.157	0.173	0.824	0.364	0.855(0.609-1.199)	-0.182	0.322	0.319	0.572	0.833(0.443-1.568)	-0.102	0.207	0.246	0.620	0.903(0.602-1.353)
Alcohol drinking	0.049	0.181	0.072	0.788	1.050(0.736-1.498)	0.073	0.337	0.047	0.828	1.076(0.555-2.085)	0.065	0.218	0.089	0.766	1.067(0.696-1.636)
Diabetes	0.176	0.164	1.151	0.283	1.192(0.865-1.643)	0.216	0.300	0.520	0.471	1.241(0.690-2.234)	0.093	0.197	0.224	0.636	1.098(0.747-1.614)
Hypertension	0.120	0.139	0.748	0.387	1.127(0.859-1.479)	0.301	0.260	1.337	0.248	1.351(0.811-2.249)	-0.024	0.166	0.021	0.884	0.976(0.706-1.350)
GLU	-0.037	0.025	2.212	0.137	0.964(0.919-1.012)	-0.067	0.051	1.755	0.185	0.935(0.847-1.033)	-0.030	0.028	1.156	0.282	0.971(0.919-1.025)
TC	0.144	0.094	2.343	0.126	1.155(0.960-1.388)	0.024	0.189	0.015	0.901	1.024(0.707-1.483)	0.178	0.104	2.936	0.087	1.195(0.975-1.464)
BUN	0.109	0.035	9.712	**0.002**	1.115(1.041-1.194)	0.070	0.065	1.159	0.282	1.073(0.944-1.219)	0.127	0.042	9.039	**0.003**	1.135(1.045-1.232)
LDL-C	-0.209	0.119	3.088	0.079	0.811(0.642-1.024)	-0.078	0.231	0.115	0.735	0.925(0.587-1.455)	-0.242	0.136	3.173	0.075	0.785(0.602-1.025)
Post-dilatation	0.135	0.138	0.956	0.328	1.145(0.873-1.501)	-0.066	0.255	0.066	0.797	0.936(0.568-1.544)	0.255	0.166	2.358	0.125	1.291(0.932-1.789)
No. of vascular lesions	0.277	0.161	2.955	0.086	1.319(0.962-1.810)	0.456	0.310	2.165	0.141	1.577(0.860-2.894)	0.264	0.191	1.907	0.167	1.302(0.895-1.893)
CTO	0.466	0.148	9.861	**0.002**	1.594(1.191-2.132)	0.629	0.279	5.057	**0.025**	1.875(1.084-3.242)	0.338	0.178	3.613	0.057	1.403(0.990-1.989)
ML	-0.105	0.298	0.124	0.724	0.900(0.502-1.614)	-0.376	0.585	0.413	0.520	0.686(0.218-2.162)	-0.030	0.348	0.007	0.932	0.971(0.491-1.920)
RPP	0.518	0.134	14.812	**<0.001**	1.678(1.289-2.184)	0.249	0.268	0.862	0.353	1.282(0.758-2.168)	0.550	0.160	11.867	**0.001**	1.733(1.267-2.369)

Note: *GLU, glucose; TC, total cholesterol; BUN, blood urea nitrogen; LDL-C, low density lipoprotein cholesterol; CTO, chronic total occlusion lesions; ML, multi-vessel lesions; RPP, rate pressure product.*

For peer review only

Table 7 Multivariable Cox regression analysis for MACCEs

Variables	Total					ACS					Stable CAD				
	B	SE	Wald	*P* values	*HR* (95% *CI*)	B	SE	Wald	*P* values	*HR* (95% *CI*)	B	SE	Wald	*P* values	*HR* (95% *CI*)
Age	-0.002	0.004	0.332	0.565	0.998(0.991-1.005)	-0.001	0.006	0.037	0.847	0.999(0.987-1.011)	-0.002	0.004	0.249	0.617	0.998(0.989-1.006)
Male	-0.144	0.092	2.454	0.117	0.866(0.723-1.037)	-0.060	0.155	0.150	0.698	0.942(0.694-1.277)	-0.182	0.115	2.529	0.112	0.833(0.665-1.043)
Smoking	-0.217	0.091	5.704	**0.017**	0.805(0.673-0.962)	-0.298	0.160	3.441	0.064	0.743(0.542-1.017)	-0.158	0.112	2.015	0.156	0.854(0.686-1.062)
Alcohol drinking	-0.048	0.096	0.250	0.617	0.953(0.790-0.151)	-0.031	0.169	0.033	0.857	0.970(0.696-1.351)	-0.054	0.118	0.208	0.649	0.948(0.752-1.194)
Diabetes	0.176	0.086	4.123	**0.042**	1.192(1.006-1.412)	0.078	0.152	0.261	0.610	1.081(0.803-1.455)	0.207	0.106	3.817	0.051	1.230(0.999-1.514)
Hypertension	0.314	0.074	18.238	**<0.001**	1.369(1.185-1.581)	0.384	0.128	8.964	**0.003**	1.468(1.142-1.887)	0.252	0.091	7.566	**0.006**	1.286(1.075-1.539)
GLU	-0.005	0.012	0.143	0.705	0.995(0.972-1.019)	-0.023	0.023	0.977	0.323	0.978(0.935-1.023)	<0.001	0.014	0.001	0.978	1.000(0.972-1.028)
TC	0.019	0.054	0.121	0.728	1.019(1.916-1.134)	-0.051	0.094	0.287	0.592	0.951(0.790-1.144)	0.048	0.065	0.553	0.457	1.049(0.925-1.191)
BUN	0.055	0.020	7.464	**0.006**	1.057(1.016-1.099)	0.054	0.034	2.537	0.111	1.055(0.988-1.128)	0.052	0.025	4.272	0.039	1.054(1.003-1.107)
LDL-C	-0.112	0.067	2.746	0.097	0.894(0.784-1.021)	-0.036	0.114	0.099	0.753	0.965(0.771-1.207)	-0.158	0.081	3.796	0.051	0.854(0.728-1.001)
Post-dilatation	0.040	0.073	0.292	0.589	1.040(1.9011-.201)	0.077	0.129	0.354	0.552	1.080(0.838-1.391)	0.038	0.090	0.181	0.671	1.039(0.871-1.239)
No. of vascular lesions	0.185	0.087	4.513	**0.034**	1.204(1.014-1.428)	0.256	0.151	2.865	0.091	1.291(0.960-1.736)	0.177	0.108	2.696	0.101	1.194(0.966-1.476)
CTO	0.200	0.084	5.754	**0.016**	1.222(1.037-1.439)	0.178	0.149	1.423	0.233	1.195(0.892-1.602)	0.203	0.102	3.928	**0.047**	1.225(1.002-1.497)
ML	-0.032	0.157	0.042	0.838	0.968(0.712-1.317)	-0.095	0.276	0.117	0.732	0.910(0.529-1.564)	-0.035	0.192	0.034	0.854	0.965(0.662-1.407)
RPP	0.234	0.074	9.840	**0.002**	1.263(1.092-1.462)	0.196	0.136	2.088	0.148	1.216(0.933-1.587)	0.239	0.091	6.956	**0.008**	1.271(1.063-1.518)

Note: *GLU, glucose; TC, total cholesterol; BUN, blood urea nitrogen; LDL-C, low density lipoprotein cholesterol; CTO, chronic total occlusion lesions; ML, multi-vessel lesions; RPP, rate pressure product.*

For peer review only

Table 8 Multivariable Cox regression analysis for MACEs

Variables	Total					ACS					Stable CAD				
	B	SE	Wald	*P* values	*HR* (95% *CI*)	B	SE	Wald	*P* values	*HR* (95% *CI*)	B	SE	Wald	*P* values	*HR* (95% *CI*)
Age	-0.004	0.004	1.34	0.247	0.996(0.989-1.003)	-0.003	0.006	0.201	0.654	0.997(0.985-1.01)	-0.005	0.005	1.069	0.301	0.995(0.986-1.004)
Male	-0.115	0.097	1.391	0.238	0.892(0.737-1.079)	-0.051	0.164	0.095	0.758	0.951(0.689-1.312)	-0.147	0.121	1.47	0.225	0.864(0.681-1.095)
Smoking	-0.156	0.095	2.721	0.099	0.855(0.710-1.030)	-0.230	0.167	1.894	0.169	0.795(0.573-1.102)	-0.101	0.116	0.751	0.386	0.904(0.720-1.136)
Alcohol drinking	-0.054	0.100	0.289	0.591	0.948(0.780-1.152)	-0.045	0.176	0.066	0.797	0.956(0.677-1.349)	-0.052	0.122	0.183	0.669	0.949(0.747-1.206)
Diabetes	0.167	0.091	3.366	0.067	1.181(1.989-1.411)	0.068	0.160	0.181	0.670	1.070(0.783-1.464)	0.194	0.111	3.059	0.080	1.214(0.977-1.510)
Hypertension	0.313	0.077	16.525	**<0.001**	1.368(1.176-1.591)	0.395	0.134	8.625	**0.003**	1.484(1.140-1.932)	0.247	0.096	6.622	**0.010**	1.28(1.061-1.544)
GLU	-0.004	0.013	0.091	0.763	0.996(0.972-1.021)	-0.027	0.024	1.229	0.268	0.973(0.928-1.021)	0.002	0.015	0.019	0.889	1.002(0.974-1.031)
TC	0.020	0.057	0.130	0.719	1.021(1.913-1.141)	-0.055	0.100	0.303	0.582	0.947(0.778-1.151)	0.050	0.067	0.568	0.451	1.052(0.923-1.199)
BUN	0.050	0.021	5.477	**0.019**	1.051(1.008-1.096)	0.050	0.036	1.915	0.166	1.051(0.980-1.127)	0.045	0.027	2.832	0.092	1.046(0.993-1.102)
LDL-C	-0.101	0.070	2.050	0.152	0.904(0.788-1.038)	-0.034	0.121	0.078	0.780	0.967(0.763-1.225)	-0.142	0.084	2.828	0.093	0.868(0.736-1.024)
Post-dilatation	0.032	0.077	0.169	0.681	1.032(1.888-1.199)	0.005	0.134	0.001	0.971	1.005(0.773-1.307)	0.060	0.094	0.406	0.524	1.062(0.883-1.278)
No. of vascular lesions	0.185	0.091	4.142	**0.042**	1.203(1.007-1.437)	0.307	0.158	3.804	0.051	1.360(0.998-1.852)	0.153	0.112	1.864	0.172	1.166(0.935-1.453)
CTO	0.234	0.087	7.296	**0.007**	1.264(1.066-1.498)	0.200	0.155	1.668	0.197	1.221(0.902-1.654)	0.240	0.106	5.093	**0.024**	1.271(1.032-1.565)
ML	0.011	0.164	0.004	0.948	1.011(1.733-1.393)	-0.096	0.291	0.109	0.741	0.908(0.514-1.607)	0.029	0.200	0.021	0.884	1.030(0.696-1.524)
RPP	0.248	0.078	10.090	**0.001**	1.281(1.099-1.492)	0.17	0.143	1.423	0.233	1.186(0.896-1.569)	0.274	0.095	8.326	**0.004**	1.315(1.092-1.584)

Note: *GLU, glucose; TC, total cholesterol; BUN, blood urea nitrogen; LDL-C, low density lipoprotein cholesterol; CTO, chronic total occlusion lesions; ML, multi-vessel lesions; RPP, rate pressure product.*

Table 9 ROC analysis of different parameters for CM

Variables	AUC (95%CI)	Difference between areas (95%CI)	*Z value*	*P value*
RPP	0.586(0.573-0.598)	-	-	-
HR	0.554(0.541-0.566)	0.032(0.003-0.061)	2.183	**0.029**
SBP	0.511(0.498-0.524)	0.075(0.025-0.125)	2.923	**0.004**

Note: *RPP, rate pressure product; HR, heart rate; SBP, systolic blood pressure, AUC, area under curve.*

For peer review only

4 Discussion

Among stable CAD patients who underwent PCI, higher preoperative RPP value led to 60.5%, 73.3%, 27.1%, and 31.5% higher relative risks in ACM, CM, MACCEs and MACEs, respectively. However, there was no significant correlation between the RPP and the adverse outcomes in ACS patients. Compared with HR and BP alone, RPP has a higher predictive value for adverse clinical outcomes.

Rate pressure product is a very reliable indicator of myocardial oxygen uptake (MVO_2). MVO_2 depends on SBP, HR and ventricular wall tension. RPP is often used to evaluate workload and exercise load, and an increase in RPP indicates that subjects need to increase MVO_2 to meet the metabolic needs of the body[16, 17]. RPP in healthy normotensive young adults has a normative circadian pattern, and the RPP value is greater during the day than at night, especially in the afternoon. The circadian pattern of the large amplitude in the RPP and its sex-based differences must be taken into account when using the RPP to assess cardiac workload, risk of left ventricular hypertrophy, and efficiency of antihypertensive therapy[18]. Stegehuis et al.[19] performed intracoronary doppler flow velocity measurements to obtain Coronary Flow Reserve (CFR) and Coronary Flow Capacity (CFC) after inducing hyperemia in 390 non-obstructed vessels of patients who were scheduled for elective percutaneous coronary intervention of another vessel. Akaike's Information Criterion (AIC) revealed that the RPP is an independent predictor of CFR and CFC. Since CFR and CFC are physiological indices for the assessment of myocardial blood flow damage caused by coronary artery disease, we can clarify the correlation between the RPP and the prognosis of CAD patients who underwent PCI.

Furthermore, Pathophysiological studies indicated that a relatively high HR has direct detrimental effects on the progression of coronary atherosclerosis, on the occurrence of myocardial ischemia and ventricular arrhythmias, and on left ventricular function[20]. Previous studies have demonstrated that elevated HR was an independent risk factor for long-term adverse prognosis in CAD patients after PCI who had postoperative stable angina or acute coronary syndrome[21-23]. O'Brien et al.[24] observed a correlation between the HR and the prognosis of CAD patients who underwent PCI and found that HR before PCI was an independent predictor of adverse 30-day cardiovascular outcomes. In addition, BP and pulse pressure (PP) were well-known independent cardiovascular risk factors. Compared brachial PP, aortic PP was better to evaluate the extent of coronary artery disease[25]. SBP was found to be a risk factor for cardiac death in CAD patients. BP before PCI was associated with long-term prognosis in CAD patients, and the long-term mortality rate of CAD patients with low systolic and high throbbing pressure was significantly reduced. In other words, a wider PP could be used as a risk factor for CAD patients who underwent PCI[26-28]. A previous study indicated that "the oscillatory gap", which was defined by the difference between the oscillatory systolic blood pressure (OSBP) and the auscultatory systolic blood pressure (AUSBP), can be used to predict the presence of CAD[29]. RPP combined the information of HR and SBP, and both the indicators were related to the prognosis of CAD patients. Compared with SBP, DBP, MAP (average arterial pressure), HR and other indicators, RPP may have a higher predictive value[12], which was consistent with our study.

At present, most studies mainly took RPP as one of the important evaluation indicators of disease-related rehabilitation training, which provided patients with reasonable exercise training guidelines for rehabilitation[30, 31]. Moore et al.[32] found that the overall risk of cancer mortality increased significantly in patients with no physical activity but with a higher RPP. In this study, we found that the CAD patients

For peer review only - http://bmjopen.bmj.com/site/about/guidelines.xhtml

who underwent PCI in the high-value group had higher incidence of mortality, MACCEs and MACEs.

There are many advantages. The first is the larger sample size, which can improve the scientific nature of the statistics. The second is the long follow-up time, which can further improve the reliability of the statistical results. The third is that RPP combines the information of two indicators and is simple and easy to obtain. However, its limitations cannot be ignored. First, we only collected heart rate and blood pressure data at the first hospitalization, thus lacking information on dynamic changes in these variables. Second, the present study did not collect the data of LVMI (left ventricular mass indix) and not rule out the effect of the patient's current medication such as antihypertensive drugs on blood pressure levels, which may have impacted the results. Third, this study had a single-centre retrospective cohort design, and our results need to be further validated through large multi-centre randomized controlled experiments. And follow-up was mainly based on telephone contacts, as well as the method of measurement was mainly based on manual, which may have biased the events. Finally, the linearity cannot be fully addressed for covariates in the Cox model.

There is a problem worth exploring. Tan Xu et al.[33] found that high RPP was associated with MACEs for ACS patients who underwent PCI. However, the predictive value of the RPP weakened when adjusting for HR. As a result, they have shown that RPP may reflect the predictive power of HR for ACS patients who underwent PCI, which was inconsistent with our study. This may be because the study involved a follow-up for of only two years, and the study population and results were different; in particular, cardiogenic shock patients and inpatients with MACEs in that study were excluded.

5 Conclusion

In summary, RPP is an independent predictor of long-term prognosis in CAD patients who underwent PCI. Therefore, the risk of disease can be stratified according to the RPP before PCI, which provides a basis for the treatment strategy of CAD patients, and a new concept for preventing the occurrence of clinical outcomes.

Contributors: Conceived and designed the research: Xiang Xie and Yi -Tong Ma. Performed the research: Zhi-hui Jiang, Analysed the data: Zhi-hui Jiang, Abudula Aierken, Ting-Ting Wu, Ying-Ying Zheng. Quality control of the study and revision: Xiang Xie, Yi -Tong Ma. Wrote the paper: Zhi-hui Jiang.

Funding: This work was funded by the National Natural Science Foundation of China (82170345).

Competing interests: The authors declare that there are no conflicts of interest regarding the publication of this paper.

Patient consent for publication: Not applicable.

Ethics approval：Ethics approval was provided by the Ethical Review Board of the First Affiliated Hospital of Xinjiang Medical University. Because the study was a retrospective cohort study based on real-world situations, there was no need to obtain informed consent from the patients.

Data availability statement: The data used to support the findings of this research are available from the corresponding author upon request.

Acknowledgements: The authors are grateful to the Department of Cardiology at the First Affiliated Hospital of Xinjiang Medical University for their support and expertise in conducting this study.

Open access: This is an open access article distributed in accordance with the Creative

Commons Attribution Non Commercial (CC BY-NC 4.0) license, which permits others to distribute, remix, adapt, build upon this work non-commercially, and license their derivative works on different terms, provided the original work is properly cited, appropriate credit is given, any changes made indicated, and the use is non-commercial. See: http://creativecommons.org/licenses/by-nc/4.0/.

Reference

1. De Backer G, Ambrosioni E, Borch-Johnsen K, et al. European Society of Cardiology Committee for Practice Guidelines. European guidelines on cardiovascular disease prevention in clinical practice: third joint task force of European and other societies on cardiovascular disease prevention in clinical practice (constituted by representatives of eight societies and by invited experts). *Eur J Cardiovasc Prev Rehabil.* 2003;10(4):S1-S10.
2. Sia CH, Ko J, Zheng H, et al. Association between smoking status and outcomes in myocardial infarction patients undergoing percutaneous coronary intervention. *Sci Rep.* 2021;11(1):6466.
3. Kuehnemund L, Koeppe J, Feld J, et al. Gender differences in acute myocardial infarction-A nationwide German real-life analysis from 2014 to 2017. *Clin Cardiol.* 2021;44(7):890-898.
4. Xu JJ, Jiang L, Song Y, et al. [Related factors and the long-term outcome after percutaneous coronary intervention of premature acute myocardial infarction]. *Zhonghua Xin Xue Guan Bing Za Zhi.* 2020;48(8):655-660.
5. Malakar AK, Choudhury D, Halder B, et al. A review on coronary artery disease, its risk factors, and therapeutics. *J Cell Physiol.* 2019;234(10):16812-16823.
6. Nishihira K, Watanabe N, Kuriyama N, et al. Clinical outcomes of nonagenarians with acute myocardial infarction who undergo percutaneous coronary intervention. *Eur Heart J Acute Cardiovasc Care.* 2020;9(5):488-495.
7. Ahmad Y, Howard JP, Arnold A, et al. Complete Revascularization by Percutaneous Coronary Intervention for Patients With ST-Segment-Elevation Myocardial Infarction and Multivessel Coronary Artery Disease: An Updated Meta-Analysis of Randomized Trials. *J Am Heart Assoc.* 2020;9(12):e015263.
8. Oh YS, Lee HJ, Lee J, et al. Long-term clinical outcomes after a percutaneous coronary intervention with a drug-eluting stent in patients with unprotected left main coronary artery disease excluded from clinical trials. *Coron Artery Dis.* 2019;30(4):239-248.
9. Whitman M, Jenkins C, Sabapathy S, et al. Comparison of Heart Rate Blood Pressure Product Versus Age-Predicted Maximum Heart Rate as Predictors of Cardiovascular Events During Exercise Stress Echocardiography. *Am J Cardiol.* 2019;124(4):528-533.
10. Berényi I, Hajduczki IS, Böszörményi E. Quantitative evaluation of exercise-induced ST-segment depression for estimation of degree of coronary artery disease. *Eur Heart J.* 1984;5(4):289-294.
11. Cook CM, Ahmad Y, Howard JP, et al. Impact of Percutaneous Revascularization on Exercise Hemodynamics in Patients With Stable Coronary Disease. *J Am Coll Cardiol.* 2018;72(9):970-983.
12. Yazdani B, Kleber ME, Yücel G, et al. Association of double product and pulse pressure with cardiovascular and all-cause mortality in the LURIC study. *J Clin Hypertens (Greenwich).* 2020;22(12):2332-2342.
13. American Diabetes Association. 2. Classification and Diagnosis of Diabetes: Standards of Medical Care in Diabetes-2021. *Diabetes Care.* 2021;44(Suppl

For peer review only - http://bmjopen.bmj.com/site/about/guidelines.xhtml

1):S15-S33.
14. Unger T, Borghi C, Charchar F, et al. 2020 International Society of Hypertension Global Hypertension Practice Guidelines. *Hypertension.* 2020;75(6):1334-1357.
15. Park JY, Choi BG, Rha SW, et al. Five-year outcomes in patients with anemia on admission undergoing a coronary intervention for acute myocardial infarction in Koreans: propensity score matching analysis. *Coron Artery Dis.* 2018;29(8):647-651.
16. Gobel FL, Norstrom LA, Nelson RR, et al. The rate-pressure product as an index of myocardial oxygen consumption during exercise in patients with angina pectoris. *Circulation.* 1978;57(3):549-556.
17. Guzun R, Kaambre T, Bagur R, et al. Modular organization of cardiac energy metabolism: energy conversion, transfer and feedback regulation. *Acta Physiol (Oxf).* 2015;213(1):84-106.
18. Hermida RC, Fernández JR, Ayala DE, et al. Circadian rhythm of double (rate-pressure) product in healthy normotensive young subjects. *Chronobiol Int.* 2001;18(3):475-489.
19. Stegehuis VE, Wijntjens GWM, Bax M, et al. Impact of clinical and haemodynamic factors on coronary flow reserve and invasive coronary flow capacity in non-obstructed coronary arteries: a patient-level pooled analysis of the DEBATE and ILIAS studies. *EuroIntervention.* 2021;16(18):e1503-e1510.
20. Fox K, Borer JS, Camm AJ, et al. Resting heart rate in cardiovascular disease. *J Am Coll Cardiol.* 2007;50(9):823-830.
21. Jensen MT, Kaiser C, Sandsten KE, et al. Heart rate at discharge and long-term prognosis following percutaneous coronary intervention in stable and acute coronary syndromes--results from the BASKET PROVE trial. *Int J Cardiol.* 2013;168(4):3802-6.
22. Zheng YY, Wu TT, Chen Y, et al. Resting Heart Rate and Long-Term Outcomes in Patients with Percutaneous Coronary Intervention: Results from a 10-Year Follow-Up of the CORFCHD-PCI Study. *Cardiol Res Pract.* 2019;2019:5432076.
23. Fox K, Ford I, Steg PG, et al. Heart rate as a prognostic risk factor in patients with coronary artery disease and left-ventricular systolic dysfunction (BEAUTIFUL): a subgroup analysis of a randomised controlled trial. *Lancet.* 2008;372(9641):817-821.
24. O'Brien J, Reid CM, Andrianopoulos N, et al. Heart Rate as a Predictor of Outcome Following Percutaneous Coronary Intervention. *Am J Cardiol.* 2018;122(7):1113-1120.
25. El Tahlawi M, Abdelbaset M, Gouda M, et al. Can we predict the presence of coronary lesions from blood pressure measurement? A new clinical method. *Hypertens Res.* 2015;38(4):260-263.
26. Warren J, Nanayakkara S, Andrianopoulos N, et al. Impact of PreProcedural Blood Pressure on Long-Term Outcomes Following Percutaneous Coronary Intervention. *J Am Coll Cardiol.* 2019;73(22):2846-2855.
27. Kawashiri MA, Sakata K, Hayashi K, et al. Impact of combined lipid lowering and blood pressure control on coronary plaque: myocardial ischemia treated by percutaneous coronary intervention and plaque regression by lipid lowering and blood pressure controlling assessed by intravascular ultrasonography (MILLION) study. *Heart Vessels.* 2017;32(5):539-548.
28. Shiraishi J, Kohno Y, Sawada T, et al. Prognostic impact of systolic blood pressure at admission on in-hospital outcome after primary percutaneous coronary intervention for acute myocardial infarction. *J Cardiol.* 2012;60(2):139-144.

For peer review only

29. Philippe F, Chemaly E, Blacher J, et al. Aortic pulse pressure and extent of coronary artery disease in percutaneous transluminal coronary angioplasty candidates. *Am J Hypertens.* 2002;15(8):672-677.
30. Carter SJ, Hunter GR, McAuley E, et al. Lower rate-pressure product during submaximal walking: a link to fatigue improvement following a physical activity intervention among breast cancer survivors. *J Cancer Surviv.* 2016;10(5):927-34.
31. Brown KD, Nguyen HL, Adams J. Continuous blood pressure monitoring during high-intensity resistance training after myocardial infarction or percutaneous coronary intervention in a phase 2 cardiac rehabilitation setting. *Proc (Bayl Univ Med Cent).* 2020;33(3):342-345.
32. Moore JX, Carter SJ, Williams V, et al. Physical health composite and risk of cancer mortality in the REasons for Geographic and Racial Differences in Stroke Study. *Prev Med.* 2020;132:105989.
33. Xu T, Zhan Y, Lu N, et al. Double product reflects the association of heart rate with MACEs in acute coronary syndrome patients treated with percutaneous coronary intervention. *BMC Cardiovasc Disord.* 2017;17(1):284.

For peer review only

Figure caption

Figure 1 The participant flow chart

Figure 2 Cumulative Kaplan-Meier estimates of the time to the first adjudicated occurrence of primary endpoints and secondary endpoints: group 1 is the low-value group with RPP<10,269, and group 2 is the high-value group with RPP≥10,269.

Figure 3 ROC curves of RPP, HR and SBP and their respective area under the curve (AUC, legend in brackets) demonstrating the ability of each parameter to predict the occurrence of cardiac death.

For peer review only

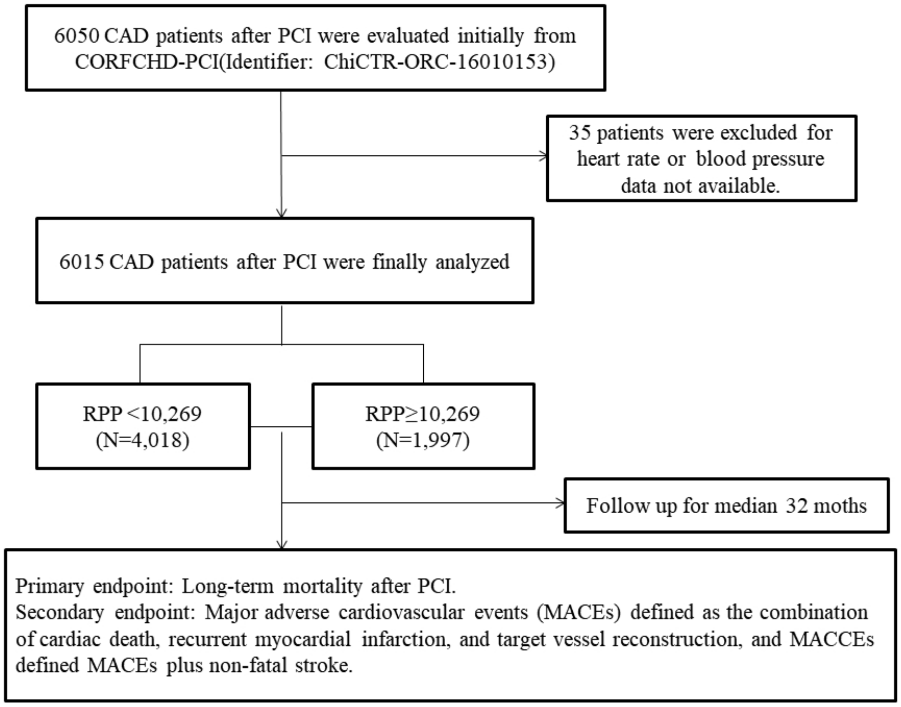

Figure 1 The participant flow chart

254x190mm (96 x 96 DPI)

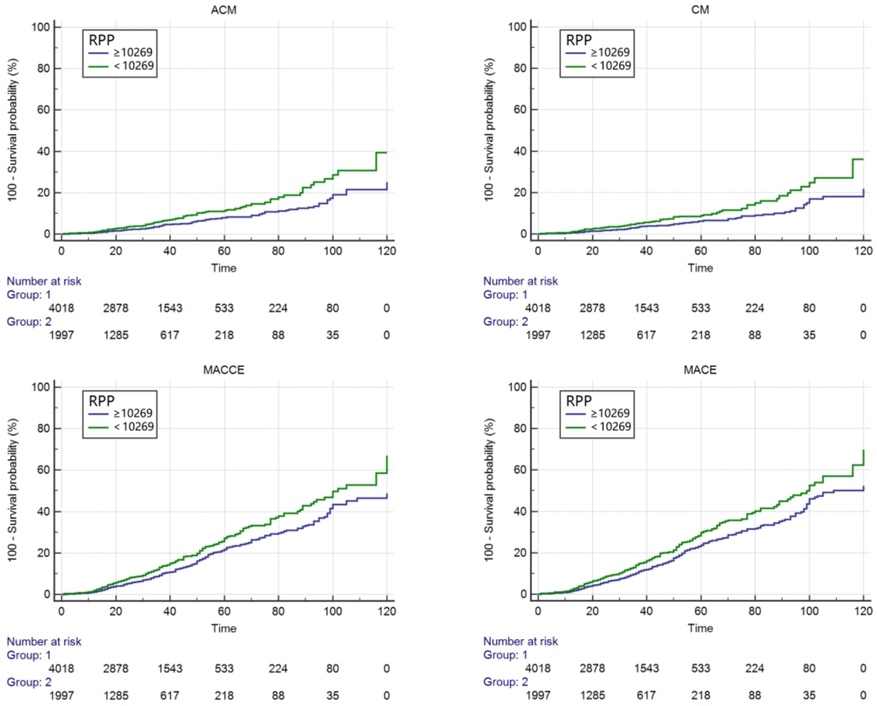

Figure 2 Cumulative Kaplan-Meier estimates of the time to the first adjudicated occurrence of primary endpoints and secondary endpoints

254x190mm (300 x 300 DPI)

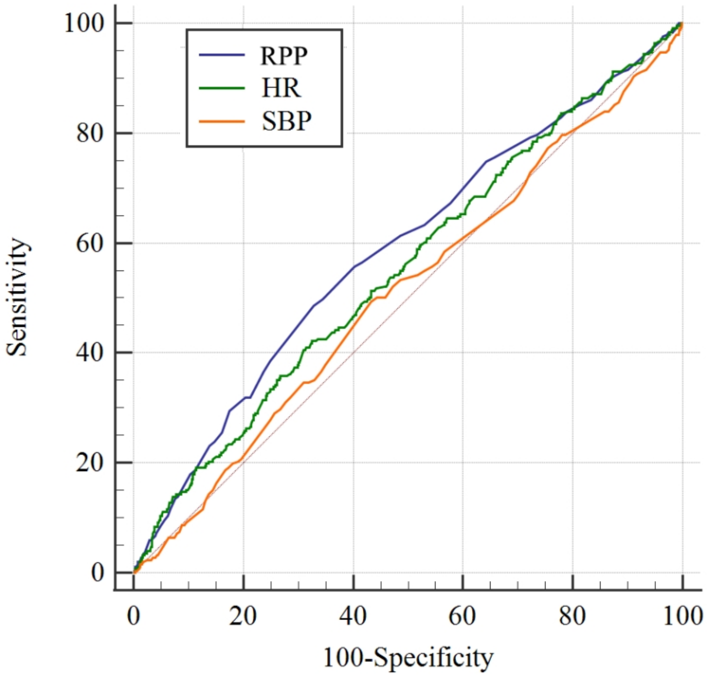

Figure 3 ROC curves of RPP, HR and SBP and their respective area under the curve (AUC, legend in brackets) demonstrating the ability of each parameter to predict the occurrence of cardiac death

101x76mm (300 x 300 DPI)

The RECORD statement: Checklist of items, extended from the STROBE statement, that should be reported in observational studies using routinely collected health data.

	Item No.	STROBE items	Location in manuscript where items are reported	RECORD items	Location in manuscript where items are reported
**Title and abstract**					
	1	(a) Indicate the study's design with a commonly used term in the title or the abstract. (b) Provide in the abstract an informative and balanced summary of what was done and what was found.	1	RECORD 1.1: The type of data used should be specified in the title or abstract. When possible, the name of the databases used should be included. RECORD 1.2: If applicable, the geographic region and time frame within which the study took place should be reported in the title or abstract. RECORD 1.3: If linkage between databases was conducted for the study, this should be clearly stated in the title or abstract.	3
Introduction					
Background rationale	2	Explain the scientific background and rationale for the investigation being reported.	2		
Objectives	3	State specific objectives, including any prespecified hypotheses.	2		
Methods					
Study design	4	Present key elements of study design early in the paper.	3		
Setting	5	Describe the setting, locations, and relevant dates including periods of recruitment, exposure, follow-up, and data collection.	3		
Participants	6	(a) Cohort study: Give the eligibility criteria and the sources and methods of selection of participants. Describe methods of follow-up. Case-control study: Give the eligibility criteria and the sources and methods of case ascertainment and control selection. Give the rationale for the choice of cases and controls. Cross-sectional study: Give the eligibility criteria and the sources and methods of selection of participants. (b) Cohort study: For matched studies, give matching criteria and number of exposed and unexposed. Case-control study: For matched studies, give matching criteria and the number of controls per case.	3	RECORD 6.1: The methods of study population selection (such as codes or algorithms used to identify subjects) should be listed in detail. If this is not possible, an explanation should be provided. RECORD 6.2: Any validation studies of the codes or algorithms used to select the population should be referenced. If validation was conducted for this study and not published elsewhere detailed methods and results should be provided. RECORD 6.3: If the study involved linkage of databases, consider use of a flow diagram or other graphical display to demonstrate the data linkage process, including the number of individuals with linked data at each stage.	Figure 1 The participant flow chart
Variables	7	Clearly define all outcomes, exposures, predictors, potential confounders, and effect modifiers. Give diagnostic criteria, if applicable.	3	RECORD 7.1: A complete list of codes and algorithms used to classify exposures, outcomes, confounders, and effect modifiers should be provided. If these cannot be reported, an explanation should be provided.	
Data sources/ measurement	8	For each variable of interest, give sources of data and details of methods of assessment (measurement). Describe comparability of assessment methods if there is more than one group.	3		
Bias	9	Describe any efforts to address potential sources of bias.	3		
Study size	10	Explain how the study size was arrived at.	3		
Quantitative variables	11	Explain how quantitative variables were handled in the analyses. If applicable, describe which groupings were chosen and why.	4		

For peer review only - http://bmjopen.bmj.com/site/about/guidelines.xhtml

Statistical methods	12	(a) Describe all statistical methods, including those used to control for confounding. (b) Describe any methods used to examine subgroups and interactions. (c) Explain how missing data were addressed. (d) Cohort study: If applicable, explain how loss to follow-up was addressed. Case-control study: If applicable, explain how matching of cases and controls was addressed. Cross-sectional study: If applicable, describe analytical methods taking account of sampling strategy. (e) Describe any sensitivity analyses.	4		
Data access and cleaning methods		N/A	4	RECORD 12.1: Authors should describe the extent to which the investigators had access to the database population used to create the study population. RECORD 12.2: Authors should provide information on the data cleaning methods used in the study. 12.3: State whether the study included person-level, institutional-level, or other data linkage across two or more databases. The methods of linkage and methods of linkage quality evaluation should be provided.	
Results					
Participants	13	(a) Report the numbers of individuals at each stage of the study (e.g., numbers potentially eligible, examined for eligibility, confirmed eligible, included in the study, completing follow-up, and analysed). (b) Give reasons for nonparticipation at each stage. (c) Consider use of a flow diagram.	4	RECORD 13.1: Describe in detail the selection of the persons included in the study (i.e., study population selection), including filtering based on data quality, data availability, and linkage. The selection of included persons can be described in the text and/or by means of the study flow diagram.	Figure 1 The participant flow chart
Descriptive data	14	(a) Give characteristics of study participants (e.g., demographic, clinical, and social) and information on exposures and potential confounders. (b) Indicate the number of participants with missing data for each variable of interest. (c) Cohort study: summarise follow-up time (e.g., average and total amount).	4		
Outcome data	15	Cohort study: Report numbers of outcome events or summary measures over time. Case-control study: Report numbers in each exposure category or summary measures of exposure. Cross-sectional study: Report numbers of outcome events or summary measures.	5-12		
Main results	16	(a) Give unadjusted estimates and, if applicable, confounder-adjusted estimates and their precision (e.g., 95% confidence interval). Make clear which confounders were adjusted for and why they were included. (b) Report category boundaries when continuous variables were categorized. (c) If relevant, consider translating estimates of relative risk into absolute risk for a meaningful time period.	5-12		
Other analyses	17	Report other analyses done—e.g., analyses of subgroups and interactions and sensitivity analyses.	8		
Discussion					

For peer review only

Key results	18	Summarise key results with reference to study objectives.	13		
Limitations	19	Discuss limitations of the study, taking into account sources of potential bias or imprecision. Discuss both direction and magnitude of any potential bias.	14	RECORD 19.1: Discuss the implications of using data that were not created or collected to answer the specific research question(s). Include discussion of misclassification bias, unmeasured confounding, missing data, and changing eligibility over time, as they pertain to the study being reported.	
interpretation	20	Give a cautious overall interpretation of results considering objectives, limitations, multiplicity of analyses, results from similar studies, and other relevant evidence.	14		
Generalisabilit y	21	Discuss the generalisability (extemal validity) of the study results.	13-14		
Other Information					
Funding	22	Give the source of funding and the role of the funders for the present study and, if applicable, for the original study on which the present article is based.	14		
Accessibility of protocol, raw data, and programming code		N/A	14-15	RECORD 22.1: Authors should provide information on how to access any supplemental information such as the study protocol, raw data, or programming code.	
N/A, not applicable					

For peer review only

BMJ Open

Rate pressure product as a novel predictor of long-term adverse outcomes in patients after percutaneous coronary intervention: A retrospective cohort study

Journal:	*BMJ Open*
Manuscript ID	bmjopen-2022-067951.R2
Article Type:	Original research
Date Submitted by the Author:	02-Mar-2023
Complete List of Authors:	Jiang, Zhi-Hui; Xinjiang Medical University Affiliated First Hospital, Department of Cardiology<br>Aierken, Abudula; Xinjiang Medical University Affiliated First Hospital<br>Wu, Ting-Ting; Xinjiang Medical University Affiliated First Hospital<br>Zheng, Ying-Ying; Xinjiang Medical University Affiliated First Hospital<br>Ma, Yi-Tong ; Xinjiang Medical University Affiliated First Hospital<br>Xie, Xiang; Xinjiang Medical University Affiliated First Hospital, Department of Cardiology
**Primary Subject Heading**:	Cardiovascular medicine
Secondary Subject Heading:	Cardiovascular medicine, Geriatric medicine, Medical management
Keywords:	Coronary heart disease < CARDIOLOGY, Coronary intervention < CARDIOLOGY, Cardiology < INTERNAL MEDICINE, Myocardial infarction < CARDIOLOGY, GERIATRIC MEDICINE

SCHOLARONE™
Manuscripts

I, the Submitting Author has the right to grant and does grant on behalf of all authors of the Work (as defined in the below author licence), an exclusive licence and/or a non-exclusive licence for contributions from authors who are: i) UK Crown employees; ii) where BMJ has agreed a CC-BY licence shall apply, and/or iii) in accordance with the terms applicable for US Federal Government officers or employees acting as part of their official duties; on a worldwide, perpetual, irrevocable, royalty-free basis to BMJ Publishing Group Ltd ("BMJ") its licensees and where the relevant Journal is co-owned by BMJ to the co-owners of the Journal, to publish the Work in this journal and any other BMJ products and to exploit all rights, as set out in our licence.

The Submitting Author accepts and understands that any supply made under these terms is made by BMJ to the Submitting Author unless you are acting as an employee on behalf of your employer or a postgraduate student of an affiliated institution which is paying any applicable article publishing charge ("APC") for Open Access articles. Where the Submitting Author wishes to make the Work available on an Open Access basis (and intends to pay the relevant APC), the terms of reuse of such Open Access shall be governed by a Creative Commons licence – details of these licences and which Creative Commons licence will apply to this Work are set out in our licence referred to above.

Other than as permitted in any relevant BMJ Author's Self Archiving Policies, I confirm this Work has not been accepted for publication elsewhere, is not being considered for publication elsewhere and does not duplicate material already published. I confirm all authors consent to publication of this Work and authorise the granting of this licence.

For peer review only

Rate pressure product as a novel predictor of long-term adverse outcomes in patients after percutaneous coronary intervention: A retrospective cohort study

Zhi-Hui Jiang, Abudula Aierken, Ting-Ting Wu, Ying-Ying Zheng, Yi-Tong Ma, Xiang Xie*

* Correspondence: xiangxie999@sina.com

Department of Cardiology, First Affiliated Hospital of Xinjiang Medical University, Urumqi 830011, People's Republic of China

Abstract

Objectives:

Previous studies have suggested that heart rate (HR) and blood pressure (BP) play important roles in the development of adverse outcomes in patients with coronary artery disease (CAD) who underwent percutaneous coronary intervention (PCI). However, the relationship between the rate pressure product (RPP) and long-term outcomes has rarely been investigated. This study investigated the effects of RPP on the clinical outcomes of CAD patients who underwent PCI.

Methods:

In this study, a total of 6,015 CAD patients were enrolled. All patients were from the CORFCHD-PCI study. They were divided into two groups according to RPP (RPP<10,269, *n*=4,018 and RPP≥10,269, *n*=1,997). In addition, the median follow-up time was 32 months. The primary endpoint was long-term mortality, including all-cause mortality (ACM) and cardiac mortality (CM). The secondary endpoints were major adverse cardiovascular events (MACEs) and major adverse cardiovascular and cerebrovascular events (MACCEs).

Results:

We found that there were significant differences between the two groups in the incidences of ACM, CM, MACCEs and MACEs (all *P*<0.05). Among the CAD patients with ACM, CM, MACCEs and MACEs, the mean survival time of the low-value group was significantly higher than that of the high-value group. Multivariate Cox regression analyses showed that RPP was an independent predictor for ACM (hazard ratio (*HR*)=1.605, 95% confidence interval (*CI*): 1.215-2.120, *P*=0.001), CM (*HR*=1.733, 95% *CI*: 1.267-2.369, *P*=0.001), MACCEs (*HR*=1.271, 95% *CI*: 1.063-1.518, *P*=0.008) and MACEs (*HR*=1.315, 95% *CI*: 1.092-1.584, *P*=0.004) in stable CAD patients. While there was no significant correlation between the RPP and the adverse outcomes in acute coronary syndrome (ACS) patients.

Conclusion:

In summary, RPP is an independent predictor of long-term prognosis in CAD patients who underwent PCI. A higher baseline RPP before PCI increased the risk of adverse outcomes. Compared with heart rate and blood pressure alone, RPP has a higher predictive value for adverse clinical outcomes.

Keywords: Coronary artery disease, Rate pressure product, Mortality, Outcomes, Percutaneous coronary intervention

Strengths and limitations of this study

a) The present study was a single-centre retrospective cohort study with a larger sample number and a long follow-up time, which can improve the scientific nature

For peer review only

of the results.

b) The present study only collected heart rate and blood pressure data at the first hospitalization, thus lacking information on dynamic changes in these variables.
c) The present study did not rule out the effect of the patient's current medication such as antihypertensive drugs on blood pressure levels.
d) Follow-up was mainly based on telephone contacts and the method of measurement was mainly based on manual, which may have impacted the results.

1 Introduction

Coronary atherosclerotic heart disease refers to the occurrence of atherosclerosis in the coronary arteries, which narrows or obstructs the arteries, causing local myocardial ischemia, hypoxia, and even necrosis, resulting in changes in heart structure and loss of function. Together with coronary artery spasms are collectively called coronary artery disease (CAD)[1]. CAD is one of the main causes of death and disability in China, and is a serious threat to health. The WHO classifies CAD into 5 clinical types: silent myocardial ischemia (latent coronary artery disease), angina pectoris, myocardial infarction, ischemia heart failure (ischemic heart disease) and sudden cardiac death. The related risk factors include sex, age, smoking, insufficient exercise, obesity, diabetes, dyslipidemia and metabolic disorders, hypertension, family history of CAD and others[2-5]. At present, percutaneous coronary intervention (PCI) is one of the most important methods for the treatment of coronary artery disease, and can significantly reduce the mortality and risk of reischemic attacks[6-8]. As an established measure of myocardial load, rate pressure product (RPP) may provide a more reliable assessment of cardiac workload[9]. Previous studies have shown that RPP can be used as an auxiliary reference index for coronary artery disease, which is negatively correlated with the degree of coronary artery disease and the number of diseased branches and negatively correlated with age and sex[10]. RPP indirectly reflects myocardial blood supply and oxygen consumption. Cook et al. [11] found that PCI can increase myocardial workload. The change observed immediately following PCI was caused by the abolition of stenosis resistance. Yazdani et al.[12] found that compared with systolic blood pressure (SBP), diastolic blood pressure (DBP), and HR, RPP was a stronger predictor of all-cause mortality (ACM) and cardiac mortality (CM) in the general population and in patients with coronary artery disease, especially in the cohort of patients at high risk of cardiovascular disease. Interestingly, RPP also predicted cardiac mortality in heart failure with preserved ejection fraction (HFrEF) patients in a stronger way, while SBP and HR alone showed no significant association. Furthermore, in 3-vessel CAD patients RPP was a stronger predictor of cardiac mortality than SBP or HR alone. Therefore, especially in patients with severe coronary artery disease and heart failure, the cardiac workload given as RPP should be considered in clinical practice[12]. However, they did not clarify the predictability of RPP for the prognosis of CAD patients who underwent PCI. The RPP was defined as the product of SBP (mmHg) and HR (beats per minute), because it combined the information of the two indicators, and both indicators were related to the prognosis of CAD patients. Therefore, we speculated that RPP may affect the long-term prognosis of CAD patients after PCI. Compared with HR and SBP, RPP may have a higher predictive value for adverse clinical outcomes.

In this research, 6,015 CAD patients were included to investigate the relationship between the RPP and the clinical outcomes.

For peer review only

2 Methods

2.1 Study design and population

In this study, 6,050 CAD patients who were hospitalized at the First Affiliated Hospital of Xinjiang Medical University from January 2008 to December 2016 were evaluated. Regarding the inclusion criteria, coronary angiography confirmed that at least one major coronary artery showed stenosis ≥70%, including the left main coronary artery, left anterior descending artery, left circumflex artery, and right coronary artery, or other more important branches. All patients underwent PCI and received at least one stent via implantation. Regarding the exclusion criteria, patients with the presence of serious heart failure, rheumatic heart disease, valvular heart disease, congenital heart disease, pulmonary heart disease, serious dysfunction of the liver or kidney, acute infections, malignant tumor, alcohol abuse or blood disease were excluded. To investigate the relation between the RPP and the outcomes in CAD patients who underwent PCI, a total of 6,050 patients were evaluated initially according to the inclusion and exclusion criteria. Thirty-five patients were excluded due to incomplete follow-up data. Finally, 6,015 patients were enrolled in this study, including 2,029 ACS patients and 3,986 stable CAD patients. All CAD patients were from the Clinical Outcomes and Risk Factors of Patients with Coronary Heart Disease after PCI (CORFCHD-PCI) study. The study is a large single-centre retrospective cohort study based on medical records and the follow-up information collected at the First Affiliated Hospital of Xinjiang Medical University. The CORFCHD-PCI study complied with the Declaration of Helsinki and the protocol was approved by the Ethics Committee of the First Affiliated Hospital of Xinjiang Medical University. The details of the design have been registered at http://www.chictr.org.cn (identifier: ChiCTR-ORC-16010153). Since the study was designed retrospectively, the Ethics Committee considered that it was not necessary to obtain informed consent form the eligible patients. **Figure 1** shows the flow chart of the inclusion and exclusion criteria for the selection of 6,050 CAD patients after PCI.

Patient and Public Involvement: No patient involved.

2.2 Data collection

The cardiovascular-related risk factors, clinical data, laboratory data and interventional therapy data of all patients were collected retrospectively, including: age, sex, heart rate, blood pressure, smoking status, alcohol consumption, history of diagnosed diabetes, history of hypertension, family history of CAD, history of medication, blood urea nitrogen (BUN), serum creatinine (Cr), uric acid (UA), glucose (GLU), triglycerides (TG), total cholesterol (TC), low density lipoprotein cholesterol (LDL-C), high-density lipoprotein cholesterol (HDL-C) and left ventricular ejection fractions (LVEF). HR and BP at admission were measured accurately according to a highly standardized protocol at the first medical contact by experienced physicians using calibrated manual mercury sphygmomanometers with an appropriately sized cuff. One sphygmomanometer was placed on the right arm, and after at least 5 min of rest in the sitting position. Two HR and BP measurements were obtained and taking the average, allowing for a 1 min interval between measurements. All laboratory tests were carried out in the Medical Laboratory Centre of the First Affiliated Hospital of Xinjiang Medical University.

2.3 Clinical diagnosis

The diagnostic criteria for diabetes mellitus were a clear history of diabetes, the use of hypoglycemic agents, fasting plasma glucose ≥7.0 mmol/L, 2-h plasma glucose ≥11.1 mmol/L during oral glucose tolerance test (OGTT), A1C ≥ 6.5%(48 mmol/mol), or a random plasma glucose ≥11.1 mmol/L in a patient with classic symptoms of hyperglycemia or hyperglycemic crisis[13]. Hypertension was diagnosed when SBP was ≥140 mmHg and/or DBP was ≥ 90 mmHg following repeated examination, or treatment

For peer review only - http://bmjopen.bmj.com/site/about/guidelines.xhtml

with antihypertensive drugs[14].

2.4 Endpoints

The primary endpoint was long-term mortality, including long-term all-cause mortality (ACM) and cardiac mortality (CM). The secondary endpoints were major adverse cardiovascular events (MACEs) and major adverse cardiovascular and cerebrovascular events (MACCEs). MACEs referred to the composite endpoint of cardiac death, recurrent myocardial infarction and target vessel reconstruction. MACCEs was defined as MACEs plus nonfatal stroke[15].

2.5 Follow-Up

In this study, 6,050 CAD patients started receiving follow-up when they underwent percutaneous coronary intervention, and regular follow-up was performed at the end of 1 month, 3 months, 6 months, 1 year, 3 years, and 5 years after discharge. Outpatient review and telephone were utilized to follow-up until the end point events occurred or the study was concluded. The median follow-up time was 32 months, and the longest follow-up time was 10 years.

2.6 Statistical analyses

All statistical analyses were performed using SPSS version 26.0 (SPSS Inc, Chicago, Illinois, United States). Continuous data are presented as the mean ± standard deviation ($\bar{x} \pm s$), and categorical variables are presented as frequencies and percentages. RPP is a continuous numerical variable. Because there is no established optimal threshold for RPP, receiver operating characteristic (ROC) curve analysis was performed to assess the predictive ability of RPP in clinical outcomes. The point where the sensitivity and specificity were maximized was determined as the best cut-off point. Two samples *t*-tests or chi-square tests were used to compare the clinical data of patients in different groups and the characteristics of adverse outcomes. Kaplan-Meier analysis was used to compare the cumulative incidence rates of long-term adverse outcomes and the log-rank test was used to compare between groups. Multivariate Cox regression analysis was performed to evaluate the predictive value of RPP for clinical outcomes. The fully adjusted models for RPP included age, hypertension, diabetes mellitus, sex, smoking, alcohol consumption, LDL-C, TC, GUL, BUN, post-dilatation, the number of vascular lesions, chronic total occlusion lesions (CTO), multi-vessel lesions (ML) and aspirin. Hazard ratios (*HRs*) and 95% confidence intervals (*CIs*) were also calculated. A *P*-Value of < 0.05 was considered statistically significant.

3 Results

3.1 Baseline data

Among the 6,015 patients included in this study, 74.3% were males, 40.0% were smokers, 29.3% were drinkers, 24.1% had diabetes mellitus, 42.3% had hypertension, and the mean age of the patients was 59.48 years. During the follow-up period, a history of using calcium antagonists accounted for 11.5%, and patients using β-receptor blockers, ACEI/ARB drugs, statins, aspirin and clopidogrel accounted for 40.2%, 22.7%, 53.9%, 66.9% and 30.4%, respectively. During the 10-year follow-up, 560 (9.3%) deaths occurred, of which 251 (4.2%) patients died due to cardiovascular disease. MACCEs occurred in 859 (14.3%) patients, including 782 (13.0%) MACEs and 77 (1.28%) nonfatal strokes.

In this study, the ROC curve analysis showed that a RPP value of 10,269 was the cut-off with the highest sensitivity and specificity in terms of prognostic significance. The population clinical characteristics were grouped by dichotomy of RPP, which was based on the ROC cut-off. RPP<10,269 was considered the low-value group, and

For peer review only

RPP≥10,269 was considered the high-value group. There were 4,018 CAD patients in the low-value group and 1,997 CAD patients in the high-value group. As shown in **Table 1**, we found that there were significant differences in many variables between the two groups (all *P*<0.05), specifically, age, BUN, GLU, TC and LDL-C in the high-value group were higher than those in the low-value group. Sex, smoking, alcohol consumption, diabetes mellitus, hypertension, the therapy of statins and aspirin in the high-value group were lower than those in the low-value group. We did not find statistically significant differences between the two groups in regards of Cr, UA, TG, HDL-C, LVEF, or the therapy of calcium channel blocker (CCB), β-receptor blocker, angiotensin-converting enzyme inhibitor or angiotensin receptor blocker (ACBI or ARB), as well as clopidogrel (all *P*>0.05). In the ACS patients, we found that diabetes mellitus, hypertension, GLU, TG, TC and clopidogrel were significantly different between the two groups. In the stable CAD patients, we found that age, sex, smoking, alcohol drinking, diabetes, hypertension, BUN, UA, GLU, the therapy of aspirin and clopidogrel were significantly different between the two groups (all *P*<0.05) (online supplemental eTable 1). For PCI procedures themselves, several variables were significantly different between these two groups, especially in the ACS patients. These variables included post-dilatation pressure, CTO, ML as well as the number of vascular lesions (all *P*<0.05) (online supplemental eTable 2).

3.2 Clinical outcomes

A comparison of clinical outcomes between the two groups of CAD patients is shown in **Table 2**. For the primary endpoints, the incidence rate of all-cause death was 6.4% in the high-value group and 4.5% in the low-value group. The difference was significant (*P*=0.002); also, the incidence rate of cardiac death between the two groups showed a significant difference (5.3% vs. 3.6%, *P*=0.002). For the secondary endpoints, we found that there were significant differences between the two groups in the incidence of MACCEs (15.8% vs. 13.5%, *P*=0.020) and MACEs (14.4% vs. 12.3%, *P*=0.020).

Sub-group analysis suggested that there were significant differences in the incidence of ACM (6.9% vs. 4.8%, *P*=0.004), CM (5.7% vs. 3.7%, *P*=0.003) and MACEs (14.4% vs. 12.2%, *P*=0.040) between the high-value group and the low-value group for the stable CAD patients. For the ACS patients, we found no significant difference in adverse outcomes (all *P*>0.05) (online supplemental eTable 3).

For peer review only

Table 1 Characteristics of participants of the two groups

Variables	Total			
	RPP<10,269 (*n*=4,018)	RPP≥10,269 (*n*=1,997)	x^2 or *t*	*P* value
Age, years	58.92±10.90	60.62±10.61	-5.751	**<0.001**
Male, n (%)	3057(76.1)	1415(70.9)	19.105	**<0.001**
Smoking, n (%)	1686(42.0)	718(36.0)	20.064	**<0.001**
Alcohol drinking, n (%)	1217(30.3)	544(27.2)	5.985	**0.014**
Diabetes, n (%)	847(21.1)	600(30.0)	58.684	**<0.001**
Hypertension, n (%)	1478(36.8)	1067(53.4)	151.429	**<0.001**
BUN, mmol/L	5.48±1.65	5.59±1.72	-2.271	**0.023**
Cr, umol/L	75.78±19.55	76.03±21.97	-0.447	0.655
UA, mmol/L	323.96±89.56	322.21±91.03	0.697	0.486
GLU, mmol/L	6.41±2.99	6.92±3.39	-5.888	**<0.001**
TG, mmol/L	1.88±1.23	1.94±1.35	-1.751	0.080
TC, mmol/L	3.94±1.10	4.01±1.13	-2.305	**0.021**
LDL-C, mmol/L	2.44±0.90	2.51±0.95	-2.689	**0.007**
HDL-C, mmol/L	1.02±0.46	1.02±0.52	-0.196	0.845
CCB, n (%)	462(11.6)	227(11.4)	0.022	0.883
β-blocker, n (%)	1631(40.8)	785(39.5)	0.930	0.335
ACEI or ARB, n (%)	894(22.4)	472(23.8)	1.525	0.217
Statins, n (%)	2207(55.5)	1035(52.2)	5.480	**0.019**
LVEF, n (%)	61.10±7.09	61.00±6.97	0.454	0.650
Aspirin, n (%)	2748(68.9)	1275(64.2)	13.062	**<0.001**
Clopidogrel, n (%)	1228(30.8)	599(30.2)	0.237	**0.626**

Note: *BUN, blood urea nitrogen; UA, uric acid; Cr, creatinine; GLU, glucose; TG, triglyceride; TC, total cholesterol; LDL-C, low density lipoprotein cholesterol; HDL-C, high density lipoprotein cholesterol; CCB, calcium channel blocker; ACEI or ARB, angiotensin-converting enzyme inhibitor or angiotensin receptor blocker; LVEF, left ventricular ejection fractions.*

Table 2 Outcomes comparison between groups

Outcomes	Total			
	RPP<10,269 (*n*=4,018)	RPP≥10,269 (*n*=1,997)	x^2	*P* value
ACM, n (%)	181(4.5)	128(6.4)	9.933	**0.002**
CM, n (%)	145 (3.6)	106(5.3)	9.632	**0.002**
MACCE, n (%)	544 (13.5)	315(15.8)	5.441	**0.020**
MACE, n (%)	494 (12.3)	288(14.4)	5.336	**0.021**

Note: *ACM, all-cause mortality; CM, cardiac mortality; MACE, major adverse cardiovascular events; MACCE, major adverse cardiovascular and cerebrovascular events。*

For peer review only

In this study, baseline RPP before PCI was used as an independent variable, and ACM, CM, MACEs and MACCEs were used as the endpoints. Kaplan-Meier analysis was performed, and the log-rank test was used for comparisons between groups. Among the CAD patients with ACM, CM, MACCEs and MACEs, the mean survival time of the low-value group was significantly higher than that of the high-value group (online supplemental eTable 4). As shown in **Figure 2**, with the extension of the follow-up time, the cumulative survival rate of the patients showed a downward trend, and the decreasing rate of the high-value group was more significant than that of the low-value group. While the cumulative risk showed an upward trend, the increasing rate of the high-value group was significantly higher than that of the low-value group. The above differences were statistically significant (all $P<0.05$).

Tables 3-6 show select variables based on the aforementioned analysis. The results of collinearity analysis showed that there was no significant collinearity among age, hypertension, diabetes mellitus, sex, GLU, BUN, LDL-C, TC, smoking, alcohol consumption, post-dilatation, the number of vascular lesions, CTO, ML, aspirin and RPP. Multivariate Cox regression analysis was performed to assess the prognostic value of the RPP and adverse outcomes after adjusting for the influence of age, hypertension, diabetes mellitus, sex, GLU, BUN, LDL-C, TC, smoking, alcohol consumption and other confounding factors. In total, the results showed that higher preoperative RPP value was a risk factor for clinical outcomes in CAD patients who underwent PCI. The respective risks of ACM (HR=1.598, 95% CI: 1.262-2.024, $P<0.001$), CM (HR=1.678, 95% CI: 1.289-2.184, $P<0.001$), MACCEs (HR=1.263, 95% CI: 1.092-1.462, P=0.002) and MACEs (HR=1.281, 95% CI: 1.099-1.492, P=0.001) that occurred in the high-value group were still significantly higher than those in the low-value group. In the stable CAD patients, after performing an adjustment of confounders, the ACM, CM, MACCEs and MACEs remained significantly different. While in the ACS group, there was no significant difference in adverse outcomes after adjusting for confounders (online supplemental eTables 5-8).

Taking cardiac death as an example. **Figure 3** shows the ROC curves of RPP as well as HR and SBP for CAD patients with cardiac death. The AUC of RPP (AUC 0.586) was larger compared to HR (AUC 0.554) or SBP (AUC 0.511) (online supplemental eTable 9).

For peer review only

Table 3 Multivariable Cox regression analysis for ACM

Variables	Total				
	B	SE	Wald	*P* values	*HR* (95% *CI*)
Age	0.026	0.006	19.314	**<0.001**	1.027(1.015-1.039)
Male	-0.010	0.149	0.005	0.944	0.990(0.739-1.325)
Smoking	-0.035	0.153	0.053	0.817	0.965(0.715-1.303)
Alcohol drinking	-0.017	0.162	0.011	0.915	0.983(0.715-1.351)
Diabetes	0.032	0.149	0.047	0.828	1.033(0.772-1.382)
Hypertension	0.186	0.123	2.282	0.131	1.205(0.946-1.534)
GLU	-0.012	0.020	0.361	0.548	0.988(0.949-1.028)
TC	0.088	0.087	1.027	0.311	1.092(0.921-1.296)
BUN	0.079	0.032	6.088	**0.014**	1.082(1.016-1.153)
LDL-C	-0.172	0.109	2.480	0.115	0.842(0.679-1.043)
Post-dilatation	0.132	0.124	1.140	0.286	1.141(0.896-1.454)
No. of vascular lesions	0.180	0.143	1.575	0.209	1.197(0.904-1.584)
CTO	0.409	0.134	9.272	**0.002**	1.505(1.157-1.957)
ML	0.032	0.262	0.015	0.903	1.032(0.618-1.724)
Aspirin	-2.141	0.194	121.798	**<0.001**	0.118(0.080-0.172)
RPP	0.469	0.121	15.098	**<0.001**	1.598(1.262-2.024)

Note: *GLU, glucose; TC, total cholesterol; BUN, blood urea nitrogen; LDL-C, low density lipoprotein cholesterol; CTO, chronic total occlusion lesions; ML, multi-vessel lesions; RPP, rate pressure product.*

Table 4 Multivariable Cox regression analysis for CM

Variables	Total				
	B	SE	Wald	*P* values	*HR* (95% *CI*)
Age	0.018	0.007	7.267	**0.007**	1.018(1.005-1.031)
Male	-0.013	0.166	0.006	0.937	0.987(0.712-1.368)
Smoking	-0.157	0.173	0.824	0.364	0.855(0.609-1.199)
Alcohol drinking	0.049	0.181	0.072	0.788	1.050(0.736-1.498)
Diabetes	0.176	0.164	1.151	0.283	1.192(0.865-1.643)
Hypertension	0.120	0.139	0.748	0.387	1.127(0.859-1.479)
GLU	-0.037	0.025	2.212	0.137	0.964(0.919-1.012)
TC	0.144	0.094	2.343	0.126	1.155(0.960-1.388)
BUN	0.109	0.035	9.712	**0.002**	1.115(1.041-1.194)
LDL-C	-0.209	0.119	3.088	0.079	0.811(0.642-1.024)
Post-dilatation	0.135	0.138	0.956	0.328	1.145(0.873-1.501)
No. of vascular lesions	0.277	0.161	2.955	0.086	1.319(0.962-1.810)
CTO	0.466	0.148	9.861	**0.002**	1.594(1.191-2.132)
ML	-0.105	0.298	0.124	0.724	0.900(0.502-1.614)
Aspirin	-2.074	0.209	98.081	**<0.001**	0.126(0.083-0.189)
RPP	0.518	0.134	14.812	**<0.001**	1.678(1.289-2.184)

Note: *GLU, glucose; TC, total cholesterol; BUN, blood urea nitrogen; LDL-C, low density lipoprotein cholesterol; CTO, chronic total occlusion lesions; ML, multi-vessel lesions; RPP, rate pressure product.*

For peer review only

Table 5 Multivariable Cox regression analysis for MACCEs

Variables	Total				
	B	SE	Wald	*P* values	*HR* (95% *CI*)
Age	-0.002	0.004	0.332	0.565	0.998(0.991-1.005)
Male	-0.144	0.092	2.454	0.117	0.866(0.723-1.037)
Smoking	-0.217	0.091	5.704	**0.017**	0.805(0.673-0.962)
Alcohol drinking	-0.048	0.096	0.250	0.617	0.953(0.790-0.151)
Diabetes	0.176	0.086	4.123	**0.042**	1.192(1.006-1.412)
Hypertension	0.314	0.074	18.238	**<0.001**	1.369(1.185-1.581)
GLU	-0.005	0.012	0.143	0.705	0.995(0.972-1.019)
TC	0.019	0.054	0.121	0.728	1.019(1.916-1.134)
BUN	0.055	0.020	7.464	**0.006**	1.057(1.016-1.099)
LDL-C	-0.112	0.067	2.746	0.097	0.894(0.784-1.021)
Post-dilatation	0.040	0.073	0.292	0.589	1.040(1.9011-.201)
No. of vascular lesions	0.185	0.087	4.513	**0.034**	1.204(1.014-1.428)
CTO	0.200	0.084	5.754	**0.016**	1.222(1.037-1.439)
ML	-0.032	0.157	0.042	0.838	0.968(0.712-1.317)
Aspirin	-0.493	0.082	36.279	**<0.001**	0.611(0.520-0.717)
RPP	0.234	0.074	9.840	**0.002**	1.263(1.092-1.462)

Note: *GLU, glucose; TC, total cholesterol; BUN, blood urea nitrogen; LDL-C, low density lipoprotein cholesterol; CTO, chronic total occlusion lesions; ML, multi-vessel lesions; RPP, rate pressure product.*

Table 6 Multivariable Cox regression analysis for MACEs

Variables	Total				
	B	SE	Wald	*P* values	*HR* (95% *CI*)
Age	-0.004	0.004	1.34	0.247	0.996(0.989-1.003)
Male	-0.115	0.097	1.391	0.238	0.892(0.737-1.079)
Smoking	-0.156	0.095	2.721	0.099	0.855(0.710-1.030)
Alcohol drinking	-0.054	0.100	0.289	0.591	0.948(0.780-1.152)
Diabetes	0.167	0.091	3.366	0.067	1.181(1.989-1.411)
Hypertension	0.313	0.077	16.525	**<0.001**	1.368(1.176-1.591)
GLU	-0.004	0.013	0.091	0.763	0.996(0.972-1.021)
TC	0.020	0.057	0.130	0.719	1.021(1.913-1.141)
BUN	0.050	0.021	5.477	**0.019**	1.051(1.008-1.096)
LDL-C	-0.101	0.070	2.050	0.152	0.904(0.788-1.038)
Post-dilatation	0.032	0.077	0.169	0.681	1.032(1.888-1.199)
No. of vascular lesions	0.185	0.091	4.142	**0.042**	1.203(1.007-1.437)
CTO	0.234	0.087	7.296	**0.007**	1.264(1.066-1.498)
ML	0.011	0.164	0.004	0.948	1.011(1.733-1.393)
Aspirin	-0.583	0.086	46.323	**<0.001**	0.558(0.472-0.660)
RPP	0.248	0.078	10.090	**0.001**	1.281(1.099-1.492)

Note: *GLU, glucose; TC, total cholesterol; BUN, blood urea nitrogen; LDL-C, low density lipoprotein cholesterol; CTO, chronic total occlusion lesions; ML, multi-vessel lesions; RPP, rate pressure product.*

For peer review only

4 Discussion

Among stable CAD patients who underwent PCI, higher preoperative RPP value led to 60.5%, 73.3%, 27.1%, and 31.5% higher relative risks in ACM, CM, MACCEs and MACEs, respectively. However, there was no significant correlation between the RPP and the adverse outcomes in ACS patients. Compared with HR and BP alone, RPP has a higher predictive value for adverse clinical outcomes.

Rate pressure product is a very reliable indicator of myocardial oxygen uptake (MVO_2). MVO_2 depends on SBP, HR and ventricular wall tension. RPP is often used to evaluate workload and exercise load, and an increase in RPP indicates that subjects need to increase MVO_2 to meet the metabolic needs of the body[16, 17]. RPP in healthy normotensive young adults has a normative circadian pattern, and the RPP value is greater during the day than at night, especially in the afternoon. The circadian pattern of the large amplitude in the RPP and its sex-based differences must be taken into account when using the RPP to assess cardiac workload, risk of left ventricular hypertrophy, and efficiency of antihypertensive therapy[18]. Stegehuis et al.[19] performed intracoronary doppler flow velocity measurements to obtain Coronary Flow Reserve (CFR) and Coronary Flow Capacity (CFC) after inducing hyperemia in 390 non-obstructed vessels of patients who were scheduled for elective percutaneous coronary intervention of another vessel. Akaike's Information Criterion (AIC) revealed that the RPP is an independent predictor of CFR and CFC. Since CFR and CFC are physiological indices for the assessment of myocardial blood flow damage caused by coronary artery disease, we can clarify the correlation between the RPP and the prognosis of CAD patients who underwent PCI.

Furthermore, Pathophysiological studies indicated that a relatively high HR has direct detrimental effects on the progression of coronary atherosclerosis, on the occurrence of myocardial ischemia and ventricular arrhythmias, and on left ventricular function[20]. Previous studies have demonstrated that elevated HR was an independent risk factor for long-term adverse prognosis in CAD patients after PCI who had postoperative stable angina or acute coronary syndrome[21-23]. O'Brien et al.[24] observed a correlation between the HR and the prognosis of CAD patients who underwent PCI and found that HR before PCI was an independent predictor of adverse 30-day cardiovascular outcomes. In addition, BP and pulse pressure (PP) were well-known independent cardiovascular risk factors. Compared brachial PP, aortic PP was better to evaluate the extent of coronary artery disease[25]. SBP was found to be a risk factor for cardiac death in CAD patients. BP before PCI was associated with long-term prognosis in CAD patients, and the long-term mortality rate of CAD patients with low systolic and high throbbing pressure was significantly reduced. In other words, a wider PP could be used as a risk factor for CAD patients who underwent PCI[26-28]. A previous study indicated that "the oscillatory gap", which was defined by the difference between the oscillatory systolic blood pressure (OSBP) and the auscultatory systolic blood pressure (AUSBP), can be used to predict the presence of CAD[29]. RPP combined the information of HR and SBP, and both the indicators were related to the prognosis of CAD patients. Compared with SBP, DBP, MAP (average arterial pressure), HR and other indicators, RPP may have a higher predictive value[12], which was consistent with our study.

At present, most studies mainly took RPP as one of the important evaluation indicators of disease-related rehabilitation training, which provided patients with reasonable exercise training guidelines for rehabilitation[30, 31]. Moore et al.[32] found that the overall risk of cancer mortality increased significantly in patients with no physical activity but with a higher RPP. In this study, we found that the CAD patients

For peer review only - http://bmjopen.bmj.com/site/about/guidelines.xhtml

who underwent PCI in the high-value group had higher incidence of mortality, MACCEs and MACEs.

There are many advantages. The first is the larger sample size, which can improve the scientific nature of the statistics. The second is the long follow-up time, which can further improve the reliability of the statistical results. The third is that RPP combines the information of two indicators and is simple and easy to obtain. However, its limitations cannot be ignored. First, we only collected heart rate and blood pressure data at the first hospitalization, thus lacking information on dynamic changes in these variables. Second, the present study did not collect the data of LVMI (left ventricular mass indix) and not rule out the effect of the patient's current medication such as antihypertensive drugs on blood pressure levels, which may have impacted the results. Third, this study had a single-centre retrospective cohort design, and our results need to be further validated through large multi-centre randomized controlled experiments. And follow-up was mainly based on telephone contacts, as well as the method of measurement was mainly based on manual, which may have biased the events. Finally, the linearity cannot be fully addressed for covariates in the Cox model.

There is a problem worth exploring. Tan Xu et al.[33] found that high RPP was associated with MACEs for ACS patients who underwent PCI. However, the predictive value of the RPP weakened when adjusting for HR. As a result, they have shown that RPP may reflect the predictive power of HR for ACS patients who underwent PCI, which was inconsistent with our study. This may be because the study involved a follow-up for of only two years, and the study population and results were different; in particular, cardiogenic shock patients and inpatients with MACEs in that study were excluded.

5 Conclusion

In summary, RPP is an independent predictor of long-term prognosis in CAD patients who underwent PCI. Therefore, the risk of disease can be stratified according to the RPP before PCI, which provides a basis for the treatment strategy of CAD patients, and a new concept for preventing the occurrence of clinical outcomes.

Contributors: Conceived and designed the research: Xiang Xie and Yi -Tong Ma. Performed the research: Zhi-hui Jiang, Analysed the data: Zhi-hui Jiang, Abudula Aierken, Ting-Ting Wu, Ying-Ying Zheng. Quality control of the study and revision: Xiang Xie, Yi -Tong Ma. Wrote the paper: Zhi-hui Jiang.

Funding: This work was funded by the National Natural Science Foundation of China (82170345).

Competing interests: The authors declare that there are no conflicts of interest regarding the publication of this paper.

Patient consent for publication: Not applicable.

Ethics approval：Ethics approval was provided by the Ethical Review Board of the First Affiliated Hospital of Xinjiang Medical University. Because the study was a retrospective cohort study based on real-world situations, there was no need to obtain informed consent from the patients.

Data availability statement: The data used to support the findings of this research are available from the corresponding author upon request.

Acknowledgements: The authors are grateful to the Department of Cardiology at the First Affiliated Hospital of Xinjiang Medical University for their support and expertise in conducting this study.

Open access: This is an open access article distributed in accordance with the Creative

For peer review only

Commons Attribution Non Commercial (CC BY-NC 4.0) license, which permits others to distribute, remix, adapt, build upon this work non-commercially, and license their derivative works on different terms, provided the original work is properly cited, appropriate credit is given, any changes made indicated, and the use is non-commercial. See: http://creativecommons.org/licenses/by-nc/4.0/.

Reference

1. De Backer G, Ambrosioni E, Borch-Johnsen K, et al. European Society of Cardiology Committee for Practice Guidelines. European guidelines on cardiovascular disease prevention in clinical practice: third joint task force of European and other societies on cardiovascular disease prevention in clinical practice (constituted by representatives of eight societies and by invited experts). *Eur J Cardiovasc Prev Rehabil.* 2003;10(4):S1-S10.
2. Sia CH, Ko J, Zheng H, et al. Association between smoking status and outcomes in myocardial infarction patients undergoing percutaneous coronary intervention. *Sci Rep.* 2021;11(1):6466.
3. Kuehnemund L, Koeppe J, Feld J, et al. Gender differences in acute myocardial infarction-A nationwide German real-life analysis from 2014 to 2017. *Clin Cardiol.* 2021;44(7):890-898.
4. Xu JJ, Jiang L, Song Y, et al. [Related factors and the long-term outcome after percutaneous coronary intervention of premature acute myocardial infarction]. *Zhonghua Xin Xue Guan Bing Za Zhi.* 2020;48(8):655-660.
5. Malakar AK, Choudhury D, Halder B, et al. A review on coronary artery disease, its risk factors, and therapeutics. *J Cell Physiol.* 2019;234(10):16812-16823.
6. Nishihira K, Watanabe N, Kuriyama N, et al. Clinical outcomes of nonagenarians with acute myocardial infarction who undergo percutaneous coronary intervention. *Eur Heart J Acute Cardiovasc Care.* 2020;9(5):488-495.
7. Ahmad Y, Howard JP, Arnold A, et al. Complete Revascularization by Percutaneous Coronary Intervention for Patients With ST-Segment-Elevation Myocardial Infarction and Multivessel Coronary Artery Disease: An Updated Meta-Analysis of Randomized Trials. *J Am Heart Assoc.* 2020;9(12):e015263.
8. Oh YS, Lee HJ, Lee J, et al. Long-term clinical outcomes after a percutaneous coronary intervention with a drug-eluting stent in patients with unprotected left main coronary artery disease excluded from clinical trials. *Coron Artery Dis.* 2019;30(4):239-248.
9. Whitman M, Jenkins C, Sabapathy S, et al. Comparison of Heart Rate Blood Pressure Product Versus Age-Predicted Maximum Heart Rate as Predictors of Cardiovascular Events During Exercise Stress Echocardiography. *Am J Cardiol.* 2019;124(4):528-533.
10. Berényi I, Hajduczki IS, Böszörményi E. Quantitative evaluation of exercise-induced ST-segment depression for estimation of degree of coronary artery disease. *Eur Heart J.* 1984;5(4):289-294.
11. Cook CM, Ahmad Y, Howard JP, et al. Impact of Percutaneous Revascularization on Exercise Hemodynamics in Patients With Stable Coronary Disease. *J Am Coll Cardiol.* 2018;72(9):970-983.
12. Yazdani B, Kleber ME, Yücel G, et al. Association of double product and pulse pressure with cardiovascular and all-cause mortality in the LURIC study. *J Clin Hypertens (Greenwich).* 2020;22(12):2332-2342.
13. American Diabetes Association. 2. Classification and Diagnosis of Diabetes: Standards of Medical Care in Diabetes-2021. *Diabetes Care.* 2021;44(Suppl

For peer review only

1):S15-S33.

14. Unger T, Borghi C, Charchar F, et al. 2020 International Society of Hypertension Global Hypertension Practice Guidelines. *Hypertension.* 2020;75(6):1334-1357.
15. Park JY, Choi BG, Rha SW, et al. Five-year outcomes in patients with anemia on admission undergoing a coronary intervention for acute myocardial infarction in Koreans: propensity score matching analysis. *Coron Artery Dis.* 2018;29(8):647-651.
16. Gobel FL, Norstrom LA, Nelson RR, et al. The rate-pressure product as an index of myocardial oxygen consumption during exercise in patients with angina pectoris. *Circulation.* 1978;57(3):549-556.
17. Guzun R, Kaambre T, Bagur R, et al. Modular organization of cardiac energy metabolism: energy conversion, transfer and feedback regulation. *Acta Physiol (Oxf).* 2015;213(1):84-106.
18. Hermida RC, Fernández JR, Ayala DE, et al. Circadian rhythm of double (rate-pressure) product in healthy normotensive young subjects. *Chronobiol Int.* 2001;18(3):475-489.
19. Stegehuis VE, Wijntjens GWM, Bax M, et al. Impact of clinical and haemodynamic factors on coronary flow reserve and invasive coronary flow capacity in non-obstructed coronary arteries: a patient-level pooled analysis of the DEBATE and ILIAS studies. *EuroIntervention.* 2021;16(18):e1503-e1510.
20. Fox K, Borer JS, Camm AJ, et al. Resting heart rate in cardiovascular disease. *J Am Coll Cardiol.* 2007;50(9):823-830.
21. Jensen MT, Kaiser C, Sandsten KE, et al. Heart rate at discharge and long-term prognosis following percutaneous coronary intervention in stable and acute coronary syndromes--results from the BASKET PROVE trial. *Int J Cardiol.* 2013;168(4):3802-6.
22. Zheng YY, Wu TT, Chen Y, et al. Resting Heart Rate and Long-Term Outcomes in Patients with Percutaneous Coronary Intervention: Results from a 10-Year Follow-Up of the CORFCHD-PCI Study. *Cardiol Res Pract.* 2019;2019:5432076.
23. Fox K, Ford I, Steg PG, et al. Heart rate as a prognostic risk factor in patients with coronary artery disease and left-ventricular systolic dysfunction (BEAUTIFUL): a subgroup analysis of a randomised controlled trial. *Lancet.* 2008;372(9641):817-821.
24. O'Brien J, Reid CM, Andrianopoulos N, et al. Heart Rate as a Predictor of Outcome Following Percutaneous Coronary Intervention. *Am J Cardiol.* 2018;122(7):1113-1120.
25. El Tahlawi M, Abdelbaset M, Gouda M, et al. Can we predict the presence of coronary lesions from blood pressure measurement? A new clinical method. *Hypertens Res.* 2015;38(4):260-263.
26. Warren J, Nanayakkara S, Andrianopoulos N, et al. Impact of PreProcedural Blood Pressure on Long-Term Outcomes Following Percutaneous Coronary Intervention. *J Am Coll Cardiol.* 2019;73(22):2846-2855.
27. Kawashiri MA, Sakata K, Hayashi K, et al. Impact of combined lipid lowering and blood pressure control on coronary plaque: myocardial ischemia treated by percutaneous coronary intervention and plaque regression by lipid lowering and blood pressure controlling assessed by intravascular ultrasonography (MILLION) study. *Heart Vessels.* 2017;32(5):539-548.
28. Shiraishi J, Kohno Y, Sawada T, et al. Prognostic impact of systolic blood pressure at admission on in-hospital outcome after primary percutaneous coronary intervention for acute myocardial infarction. *J Cardiol.* 2012;60(2):139-144.

For peer review only

29. Philippe F, Chemaly E, Blacher J, et al. Aortic pulse pressure and extent of coronary artery disease in percutaneous transluminal coronary angioplasty candidates. *Am J Hypertens.* 2002;15(8):672-677.
30. Carter SJ, Hunter GR, McAuley E, et al. Lower rate-pressure product during submaximal walking: a link to fatigue improvement following a physical activity intervention among breast cancer survivors. *J Cancer Surviv.* 2016;10(5):927-34.
31. Brown KD, Nguyen HL, Adams J. Continuous blood pressure monitoring during high-intensity resistance training after myocardial infarction or percutaneous coronary intervention in a phase 2 cardiac rehabilitation setting. *Proc (Bayl Univ Med Cent).* 2020;33(3):342-345.
32. Moore JX, Carter SJ, Williams V, et al. Physical health composite and risk of cancer mortality in the REasons for Geographic and Racial Differences in Stroke Study. *Prev Med.* 2020;132:105989.
33. Xu T, Zhan Y, Lu N, et al. Double product reflects the association of heart rate with MACEs in acute coronary syndrome patients treated with percutaneous coronary intervention. *BMC Cardiovasc Disord.* 2017;17(1):284.

For peer review only

Figure caption

Figure 1 The participant flow chart

Figure 2 Cumulative Kaplan-Meier estimates of the time to the first adjudicated occurrence of primary endpoints and secondary endpoints: group 1 is the low-value group with RPP<10,269, and group 2 is the high-value group with RPP≥10,269.

Figure 3 ROC curves of RPP, HR and SBP and their respective area under the curve (AUC, legend in brackets) demonstrating the ability of each parameter to predict the occurrence of cardiac death.

For peer review only

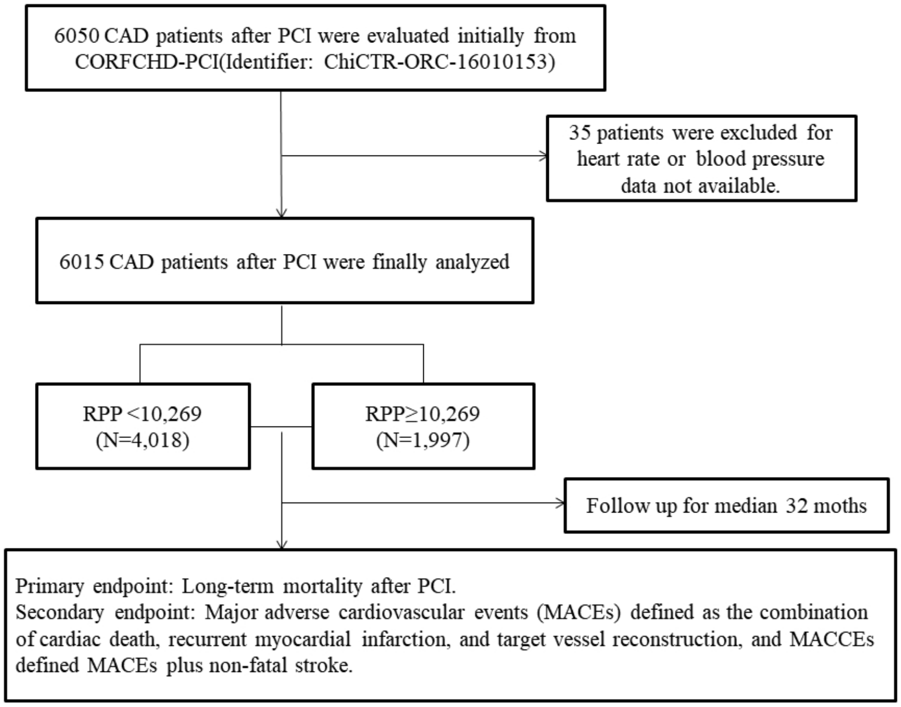

Figure 1 The participant flow chart

254x190mm (96 x 96 DPI)

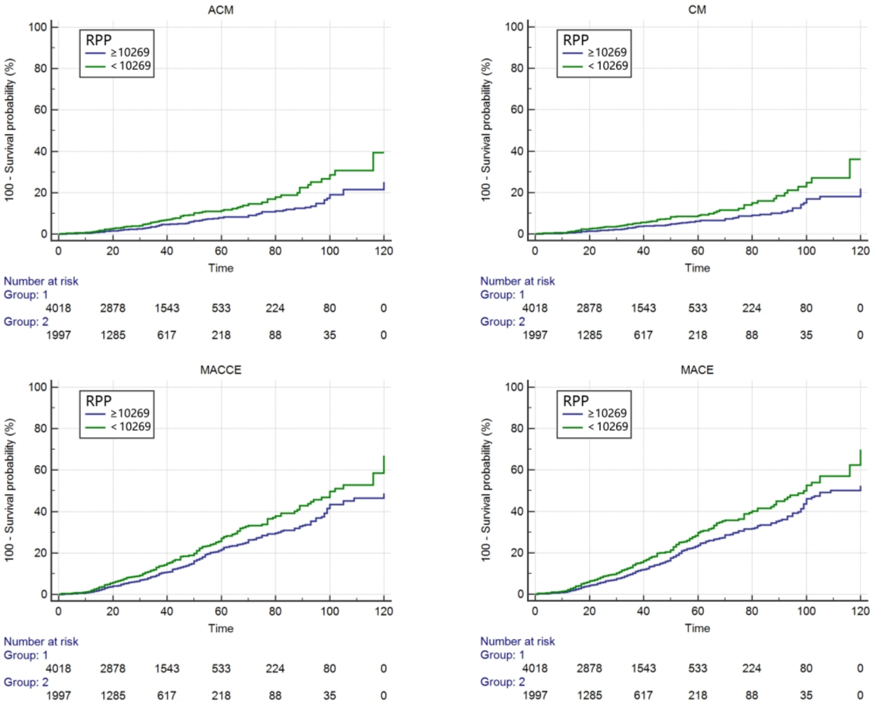

Figure 2 Cumulative Kaplan-Meier estimates of the time to the first adjudicated occurrence of primary endpoints and secondary endpoints

254x190mm (300 x 300 DPI)

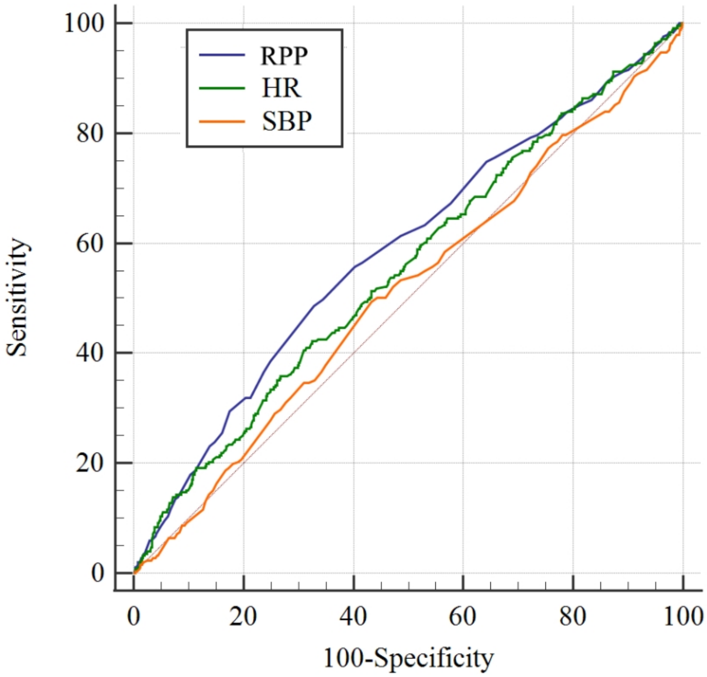

Figure 3 ROC curves of RPP, HR and SBP and their respective area under the curve (AUC, legend in brackets) demonstrating the ability of each parameter to predict the occurrence of cardiac death

101x76mm (300 x 300 DPI)

SUPPLEMENTARY MATERIAL

Supplementary Table 1 Characteristics of participants of the two groups

Variables	ACS				Stable CAD			
	RPP<10,269 (*n*=1,444)	RPP≥10,269 (*n*=585)	x^2or *t*	*P* value	RPP<10,269 (*n*=2,574)	RPP≥10,269 (*n*=1,412)	x^2or *t*	*P* value
Age, years	59.57±10.89	60.60±11.10	-1.930	0.054	56.56±10.89	60.63±10.40	-0.835	**<0.001**
Male, n (%)	358(24.8)	169(28.9)	3.634	0.057	603(23.4)	413(29.2)	16.277	**<0.001**
Smoking, n (%)	607(42.0)	228(39.0)	1.611	0.204	1079(41.9)	490(34.7)	19.895	**<0.001**
Alcohol drinking, n (%)	434(30.1)	167(28.5)	0.454	0.500	783(30.4)	377(26.7)	6.115	**0.013**
Diabetes, n (%)	301(20.8)	177(30.3)	20.478	**<0.001**	546(21.2)	423(30.0)	37.900	**<0.001**
Hypertension, n (%)	555(38.4)	309(52.8)	35.239	**<0.001**	923(35.9)	758(53.7)	118.784	**<0.001**
BUN, mmol/L	5.50±1.71	5.56±1.84	-0.695	0.487	5.48±1.61	5.60±1.67	-2.306	**0.021**
Cr, umol/L	76.00±20.77	77.59±21.79	-1.510	0.131	75.65±18.84	75.40±22.02	0.382	0.703
UA, mmol/L	320.62±89.64	328.53±92.28	-1.750	0.080	325.83±89.49	319.64±90.43	2.051	**0.040**
GLU, mmol/L	6.41±3.02	7.22±3.39	-5.209	**<0.001**	6.41±2.97	6.80±3.39	-3.706	**<0.001**
TG, mmol/L	1.88±1.25	2.04±1.39	-2.531	**0.011**	1.88±1.22	1.90±1.33	-0.470	0.638
TC, mmol/L	3.92±1.07	4.04±1.10	-2.089	**0.037**	3.95±1.11	4.00±1.14	-1.371	0.170
LDL-C, mmol/L	2.41±0.87	2.50±0.96	-1.908	0.057	2.45±0.91	2.51±0.95	-1.866	0.062
HDL-C, mmol/L	1.01±0.44	1.01±0.46	0.288	0.774	1.02±0.48	1.03±0.54	-0.361	0.718
CCB, n (%)	184(12.8)	67(11.5)	0.644	0.422	278(10.9)	160(11.4)	0.270	0.603
β-blocker, n (%)	580(40.4)	234(40.2)	0.004	0.948	1051(41.0)	551(39.2)	1.268	0.260
ACEI or ARB, n (%)	325(22.6)	142(24.4)	0.760	0.383	569(22.2)	330(23.5)	0.878	0.349
Statins, n (%)	797(55.8)	303(52.2)	2.123	0.145	1410(55.3)	732(52.2)	3.284	0.070
LVEF, n (%)	61.09±6.88	60.84±7.01	0.707	0.479	61.10±7.21	61.07±6.96	0.111	0.911
Aspirin, n (%)	988(69.0)	396(68.2)	0.134	0.714	1760(68.8)	879(62.6)	15.742	**<0.001**
Clopidogrel, n (%)	436(30.4)	213(36.7)	7.305	**0.007**	792(31.0)	386(27.5)	5.307	**0.021**

Note: *BUN, blood urea nitrogen; UA, uric acid; Cr, creatinine; GLU, glucose; TG, triglyceride; TC, total cholesterol; LDL-C, low density lipoprotein cholesterol; HDL-C, high density lipoprotein cholesterol; CCB, calcium channel blocker; ACEI or ARB, angiotensin-converting enzyme inhibitor or angiotensin receptor blocker; LVEF, left ventricular ejection fractions.*

For peer review only

Supplementary Table 2 Baseline treatments and procedure characteristics

Variables	Total				ACS				Stable CAD			
	RPP<10,269 (*n*=4,018)	RPP≥10,269 (*n*=1,997)	x^2 or t	*P* value	RPP<10,269 (*n*=1,444)	RPP≥10,269 (*n*=585)	x^2 or t	*P* value	RPP<10,269 (*n*=2,574)	RPP≥10,269 (*n*=1,412)	x^2 or t	*P* value
New generation stent, *n* (%)	3793(94.4)	1875(93.9)	0.699	0.403	1369(94.9)	553(94.5)	0.098	0.754	2424(94.2)	1322(93.6)	0.481	0.488
Diameter of stents, mm	2.85±0.37	2.85±0.37	-0.676	0.499	2.87±0.38	2.85±0.87	0.858	0.391	2.84±0.37	2.86±0.38	-1.548	0.122
Length of stents, mm	28.05±6.96	27.88±6.97	0.843	0.399	28.11±6.96	27.51±6.89	1.778	0.075	28.01±6.96	28.04±7.01	-0.143	0.886
No. of stents, *n*	1.04±0.22	1.04±0.24	-0.858	0.391	1.04±0.22	1.07±0.28	-1.901	0.057	1.04±0.22	1.04±0.22	0.193	0.847
Pre-dilatation, *n* (%)	3476(86.5)	1729(86.6)	0.003	0.959	1259(87.2)	505(86.3)	0.314	0.575	2217(86.1)	1224(86.7)	0.238	0.626
Post-dilatation, *n* (%)	2542(63.3)	1217(60.9)	3.115	0.078	913(63.3)	355(60.7)	1.189	0.276	1629(63.3)	862(61.0)	1.949	0.163
Post-dilatation pressure, atm	13.53±3.59	14.61±3.31	-9.342	**<0.001**	13.41±3.33	14.41±3.45	-5.105	**<0.001**	13.60±3.72	14.68±3.24	-7.658	**<0.001**
CTO, *n* (%)	873(21.7)	529(26.5)	16.885	**<0.001**	284(19.7)	201(34.4)	49.284	**<0.001**	589(22.9)	328(23.2)	0.062	0.804
ML, *n* (%)	2573(64.1)	1333(66.8)	4.263	**0.039**	972(67.4)	428(73.2)	6.556	**0.010**	1601(62.2)	905(64.1)	1.402	0.236
No. of vascular lesions, *n*	1.99±0.84	2.05±0.85	-2.617	**0.009**	2.06±0.84	2.18±0.83	-2.928	**0.003**	1.96±0.84	2.00±0.85	-1.606	0.108

Note: *CTO, chronic total occlusion lesions; RPP, rate pressure product; ML, multi-vessel lesions.*

For peer review only

Supplementary Table 3 Outcomes comparison between groups

Outcomes	ACS				Stable CAD			
	RPP<10,269 (*n*=1,444)	RPP≥10,269 (*n*=585)	x^2	*P* value	RPP<10,269 (*n*=2,574)	RPP≥10,269 (*n*=1,412)	x^2	*P* value
ACM, n (%)	58(4.0)	30(5.1)	1.240	0.265	123(4.8)	98(6.9)	8.138	**0.004**
CM, n (%)	49(3.4)	25(4.3)	0.918	0.338	96(3.7)	81(5.7)	8.655	**0.003**
MACCE, n (%)	198(13.7)	94(16.1)	1.876	0.171	346(13.4)	221(15.7)	3.648	0.056
MACE, n (%)	181(12.5)	84(14.4)	1.220	0.269	313(12.2)	204(14.4)	4.227	**0.040**

Note: *ACM, all-cause mortality; CM, cardiac mortality; MACE, major adverse cardiovascular events; MACCE, major adverse cardiovascular and cerebrovascular events.*

For peer review only

Supplementary Table 4 Mean Survival Time for Outcomes comparison between groups

Outcomes	Mean Survival Time(m)		
	RPP <10,269 (*n*=4,018)	RPP≥10,269 (*n*=1,997)	Overall
ACM	109.512	103.379	107.681
CM	111.362	105.887	109.731
MACCE	92.150	85.849	90.204
MACE	94.267	88.089	92.375

Note: *ACM, all-cause mortality; CM, cardiac mortality; MACE, major adverse cardiovascular events; MACCE, major adverse cardiovascular and cerebrovascular events.*

For peer review only

Supplementary Table 5 Multivariable Cox regression analysis for ACM

Variables	ACS					Stable CAD				
	B	SE	Wald	*P* values	*HR* (95% *CI*)	B	SE	Wald	*P* values	*HR* (95% *CI*)
Age	0.027	0.011	5.473	**0.019**	1.027(1.004-1.050)	0.027	0.007	14.141	**<0.001**	1.027(1.013-1.041)
Male	-0.091	0.295	0.095	0.758	0.913(0.513-1.627)	<0.001	0.173	<0.001	0.998	1.000(0.713-1.404)
Smoking	0.077	0.294	0.069	0.793	1.080(0.607-1.922)	-0.030	0.182	0.028	0.867	0.970(0.679-1.386)
Alcohol drinking	0.124	0.301	0.169	0.681	1.132(0.627-2.044)	-0.056	0.196	0.082	0.774	0.945(0.645-1.387)
Diabetes	0.079	0.275	0.083	0.773	1.082(0.631-1.855)	-0.034	0.178	0.037	0.848	0.966(0.681-1.371)
Hypertension	0.286	0.237	1.458	0.227	1.331(0.837-2.116)	0.098	0.146	0.448	0.503	1.103(0.828-1.470)
GLU	-0.012	0.039	0.099	0.753	0.988(0.915-1.066)	-0.016	0.024	0.461	0.497	0.984(0.939-1.031)
TC	-0.177	0.159	1.231	0.267	0.838(0.613-1.145)	0.175	0.092	3.606	0.058	1.191(0.994-1.427)
BUN	0.051	0.061	0.714	0.398	1.053(0.935-1.185)	0.09	0.038	5.459	0.019	1.094(1.015-1.180)
LDL-C	0.116	0.186	0.389	0.533	1.123(0.779-1.619)	-0.262	0.121	4.717	**0.030**	0.769(0.607-0.975)
Post-dilatation	-0.085	0.231	0.135	0.713	0.919(0.584-1.444)	0.257	0.148	3.005	0.083	1.293(0.967-1.728)
No. of vascular lesions	0.287	0.273	1.111	0.292	1.333(0.781-2.275)	0.190	0.170	1.243	0.265	1.209(0.866-1.687)
CTO	0.600	0.253	5.609	**0.018**	1.823(1.109-2.995)	0.263	0.161	2.681	0.102	1.301(0.949-1.783)
ML	-0.002	0.515	<0.001	0.997	0.998(0.363-2.741)	0.038	0.306	0.016	0.900	1.039(0.570-1.894)
Aspirin	-1.979	0.337	34.553	**<0.001**	0.138(0.071-0.267)	-2.194	0.239	84.425	**<0.001**	0.111(0.070-0.178)
RPP	0.249	0.242	1.058	0.304	1.283(0.798-2.064)	0.473	0.142	11.09	**0.001**	1.605(1.215-2.120)

Note: *GLU, glucose; TC, total cholesterol; BUN, blood urea nitrogen; LDL-C, low density lipoprotein cholesterol; CTO, chronic total occlusion lesions; ML, multi-vessel lesions; RPP, rate pressure product.*

For peer review only

Supplementary Table 6 Multivariable Cox regression analysis for CM

Variables	ACS					Stable CAD				
	B	SE	Wald	*P* values	*HR* (95% *CI*)	B	SE	Wald	*P* values	*HR* (95% *CI*)
Age	0.020	0.013	2.456	0.117	1.020(0.995-1.045)	0.017	0.008	4.805	**0.028**	1.017(1.002-1.033)
Male	-0.243	0.324	0.561	0.454	0.784(0.415-1.481)	0.065	0.195	0.110	0.740	1.067(0.728-1.564)
Smoking	-0.182	0.322	0.319	0.572	0.833(0.443-1.568)	-0.102	0.207	0.246	0.620	0.903(0.602-1.353)
Alcohol drinking	0.073	0.337	0.047	0.828	1.076(0.555-2.085)	0.065	0.218	0.089	0.766	1.067(0.696-1.636)
Diabetes	0.216	0.300	0.520	0.471	1.241(0.690-2.234)	0.093	0.197	0.224	0.636	1.098(0.747-1.614)
Hypertension	0.301	0.260	1.337	0.248	1.351(0.811-2.249)	-0.024	0.166	0.021	0.884	0.976(0.706-1.350)
GLU	-0.067	0.051	1.755	0.185	0.935(0.847-1.033)	-0.030	0.028	1.156	0.282	0.971(0.919-1.025)
TC	0.024	0.189	0.015	0.901	1.024(0.707-1.483)	0.178	0.104	2.936	0.087	1.195(0.975-1.464)
BUN	0.070	0.065	1.159	0.282	1.073(0.944-1.219)	0.127	0.042	9.039	**0.003**	1.135(1.045-1.232)
LDL-C	-0.078	0.231	0.115	0.735	0.925(0.587-1.455)	-0.242	0.136	3.173	0.075	0.785(0.602-1.025)
Post-dilatation	-0.066	0.255	0.066	0.797	0.936(0.568-1.544)	0.255	0.166	2.358	0.125	1.291(0.932-1.789)
No. of vascular lesions	0.456	0.310	2.165	0.141	1.577(0.860-2.894)	0.264	0.191	1.907	0.167	1.302(0.895-1.893)
CTO	0.629	0.279	5.057	**0.025**	1.875(1.084-3.242)	0.338	0.178	3.613	0.057	1.403(0.990-1.989)
ML	-0.376	0.585	0.413	0.520	0.686(0.218-2.162)	-0.030	0.348	0.007	0.932	0.971(0.491-1.920)
Aspirin	-1.937	0.368	27.721	**<0.001**	0.144(0.070-0.297)	-2.116	0.255	68.802	**<0.001**	0.121(0.073-0.199)
RPP	0.249	0.268	0.862	0.353	1.282(0.758-2.168)	0.550	0.160	11.867	**0.001**	1.733(1.267-2.369)

Note: *GLU, glucose; TC, total cholesterol; BUN, blood urea nitrogen; LDL-C, low density lipoprotein cholesterol; CTO, chronic total occlusion lesions; ML, multi-vessel lesions; RPP, rate pressure product.*

For peer review only

Supplementary Table 7 Multivariable Cox regression analysis for MACCEs

Variables	ACS					Stable CAD				
	B	SE	Wald	*P* values	*HR* (95% *CI*)	B	SE	Wald	*P* values	*HR* (95% *CI*)
Age	-0.001	0.006	0.037	0.847	0.999(0.987-1.011)	-0.002	0.004	0.249	0.617	0.998(0.989-1.006)
Male	-0.060	0.155	0.150	0.698	0.942(0.694-1.277)	-0.182	0.115	2.529	0.112	0.833(0.665-1.043)
Smoking	-0.298	0.160	3.441	0.064	0.743(0.542-1.017)	-0.158	0.112	2.015	0.156	0.854(0.686-1.062)
Alcohol drinking	-0.031	0.169	0.033	0.857	0.970(0.696-1.351)	-0.054	0.118	0.208	0.649	0.948(0.752-1.194)
Diabetes	0.078	0.152	0.261	0.610	1.081(0.803-1.455)	0.207	0.106	3.817	0.051	1.230(0.999-1.514)
Hypertension	0.384	0.128	8.964	**0.003**	1.468(1.142-1.887)	0.252	0.091	7.566	**0.006**	1.286(1.075-1.539)
GLU	-0.023	0.023	0.977	0.323	0.978(0.935-1.023)	<0.001	0.014	0.001	0.978	1.000(0.972-1.028)
TC	-0.051	0.094	0.287	0.592	0.951(0.790-1.144)	0.048	0.065	0.553	0.457	1.049(0.925-1.191)
BUN	0.054	0.034	2.537	0.111	1.055(0.988-1.128)	0.052	0.025	4.272	0.039	1.054(1.003-1.107)
LDL-C	-0.036	0.114	0.099	0.753	0.965(0.771-1.207)	-0.158	0.081	3.796	0.051	0.854(0.728-1.001)
Post-dilatation	0.077	0.129	0.354	0.552	1.080(0.838-1.391)	0.038	0.090	0.181	0.671	1.039(0.871-1.239)
No. of vascular lesions	0.256	0.151	2.865	0.091	1.291(0.960-1.736)	0.177	0.108	2.696	0.101	1.194(0.966-1.476)
CTO	0.178	0.149	1.423	0.233	1.195(0.892-1.602)	0.203	0.102	3.928	**0.047**	1.225(1.002-1.497)
ML	-0.095	0.276	0.117	0.732	0.910(0.529-1.564)	-0.035	0.192	0.034	0.854	0.965(0.662-1.407)
Aspirin	-0.463	0.141	10.742	**0.001**	0.629(0.477-0.830)	-0.507	0.101	25.196	**<0.001**	0.602(0.494-0.734)
RPP	0.196	0.136	2.088	0.148	1.216(0.933-1.587)	0.239	0.091	6.956	**0.008**	1.271(1.063-1.518)

Note: *GLU, glucose; TC, total cholesterol; BUN, blood urea nitrogen; LDL-C, low density lipoprotein cholesterol; CTO, chronic total occlusion lesions; ML, multi-vessel lesions; RPP, rate pressure product.*

For peer review only

For peer review only - http://bmjopen.bmj.com/site/about/guidelines.xhtml

Supplementary Table 8 Multivariable Cox regression analysis for MACEs

Variables	ACS					Stable CAD				
	B	SE	Wald	*P* values	*HR* (95% *CI*)	B	SE	Wald	*P* values	*HR* (95% *CI*)
Age	-0.003	0.006	0.201	0.654	0.997(0.985-1.01)	-0.005	0.005	1.069	0.301	0.995(0.986-1.004)
Male	-0.051	0.164	0.095	0.758	0.951(0.689-1.312)	-0.147	0.121	1.47	0.225	0.864(0.681-1.095)
Smoking	-0.230	0.167	1.894	0.169	0.795(0.573-1.102)	-0.101	0.116	0.751	0.386	0.904(0.720-1.136)
Alcohol drinking	-0.045	0.176	0.066	0.797	0.956(0.677-1.349)	-0.052	0.122	0.183	0.669	0.949(0.747-1.206)
Diabetes	0.068	0.160	0.181	0.670	1.070(0.783-1.464)	0.194	0.111	3.059	0.080	1.214(0.977-1.510)
Hypertension	0.395	0.134	8.625	**0.003**	1.484(1.140-1.932)	0.247	0.096	6.622	**0.010**	1.28(1.061-1.544)
GLU	-0.027	0.024	1.229	0.268	0.973(0.928-1.021)	0.002	0.015	0.019	0.889	1.002(0.974-1.031)
TC	-0.055	0.100	0.303	0.582	0.947(0.778-1.151)	0.050	0.067	0.568	0.451	1.052(0.923-1.199)
BUN	0.050	0.036	1.915	0.166	1.051(0.980-1.127)	0.045	0.027	2.832	0.092	1.046(0.993-1.102)
LDL-C	-0.034	0.121	0.078	0.780	0.967(0.763-1.225)	-0.142	0.084	2.828	0.093	0.868(0.736-1.024)
Post-dilatation	0.005	0.134	0.001	0.971	1.005(0.773-1.307)	0.060	0.094	0.406	0.524	1.062(0.883-1.278)
No. of vascular lesions	0.307	0.158	3.804	0.051	1.360(0.998-1.852)	0.153	0.112	1.864	0.172	1.166(0.935-1.453)
CTO	0.200	0.155	1.668	0.197	1.221(0.902-1.654)	0.240	0.106	5.093	**0.024**	1.271(1.032-1.565)
ML	-0.096	0.291	0.109	0.741	0.908(0.514-1.607)	0.029	0.200	0.021	0.884	1.030(0.696-1.524)
Aspirin	-0.551	0.147	13.991	**<0.001**	0.576(0.432-0.769)	-0.597	0.106	31.853	**<0.001**	0.551(0.448-0.677)
RPP	0.17	0.143	1.423	0.233	1.186(0.896-1.569)	0.274	0.095	8.326	**0.004**	1.315(1.092-1.584)

Note: *GLU, glucose; TC, total cholesterol; BUN, blood urea nitrogen; LDL-C, low density lipoprotein cholesterol; CTO, chronic total occlusion lesions; ML, multi-vessel lesions; RPP, rate pressure product.*

For peer review only

Supplementary Table 9 ROC analysis of different parameters for CM

Variables	AUC (95%*CI*)	Difference between areas (95%*CI*)	*Z value*	*P value*
RPP	0.586(0.573-0.598)	-	-	-
HR	0.554(0.541-0.566)	0.032(0.003-0.061)	2.183	**0.029**
SBP	0.511(0.498-0.524)	0.075(0.025-0.125)	2.923	**0.004**

Note: *RPP, rate pressure product; HR, heart rate; SBP, systolic blood pressure, AUC, area under curve.*

For peer review only

The RECORD statement: Checklist of items, extended from the STROBE statement, that should be reported in observational studies using routinely collected health data.

	Item No.	STROBE items	Location in manuscript where items are reported	RECORD items	Location in manuscript where items are reported
**Title and abstract**					
	1	(a) Indicate the study's design with a commonly used term in the title or the abstract. (b) Provide in the abstract an informative and balanced summary of what was done and what was found.	1	RECORD 1.1: The type of data used should be specified in the title or abstract. When possible, the name of the databases used should be included. RECORD 1.2: If applicable, the geographic region and time frame within which the study took place should be reported in the title or abstract. RECORD 1.3: If linkage between databases was conducted for the study, this should be clearly stated in the title or abstract.	3
Introduction					
Background rationale	2	Explain the scientific background and rationale for the investigation being reported.	2		
Objectives	3	State specific objectives, including any prespecified hypotheses.	2		
Methods					
Study design	4	Present key elements of study design early in the paper.	3		
Setting	5	Describe the setting, locations, and relevant dates including periods of recruitment, exposure, follow-up, and data collection.	3		
Participants	6	(a) Cohort study: Give the eligibility criteria and the sources and methods of selection of participants. Describe methods of follow-up. Case-control study: Give the eligibility criteria and the sources and methods of case ascertainment and control selection. Give the rationale for the choice of cases and controls. Cross-sectional study: Give the eligibility criteria and the sources and methods of selection of participants. (b) Cohort study: For matched studies, give matching criteria and number of exposed and unexposed. Case-control study: For matched studies, give matching criteria and the number of controls per case.	3	RECORD 6.1: The methods of study population selection (such as codes or algorithms used to identify subjects) should be listed in detail. If this is not possible, an explanation should be provided. RECORD 6.2: Any validation studies of the codes or algorithms used to select the population should be referenced. If validation was conducted for this study and not published elsewhere detailed methods and results should be provided. RECORD 6.3: If the study involved linkage of databases, consider use of a flow diagram or other graphical display to demonstrate the data linkage process, including the number of individuals with linked data at each stage.	Figure 1 The participant flow chart
Variables	7	Clearly define all outcomes, exposures, predictors, potential confounders, and effect modifiers. Give diagnostic criteria, if applicable.	3	RECORD 7.1: A complete list of codes and algorithms used to classify exposures, outcomes, confounders, and effect modifiers should be provided. If these cannot be reported, an explanation should be provided.	
Data sources/ measurement	8	For each variable of interest, give sources of data and details of methods of assessment (measurement). Describe comparability of assessment methods if there is more than one group.	3		
Bias	9	Describe any efforts to address potential sources of bias.	3		
Study size	10	Explain how the study size was arrived at.	3		
Quantitative variables	11	Explain how quantitative variables were handled in the analyses. If applicable, describe which groupings were chosen and why.	4		

For peer review only

Statistical methods	12	(a) Describe all statistical methods, including those used to control for confounding. (b) Describe any methods used to examine subgroups and interactions. (c) Explain how missing data were addressed. (d) Cohort study: If applicable, explain how loss to follow-up was addressed. Case-control study: If applicable, explain how matching of cases and controls was addressed. Cross-sectional study: If applicable, describe analytical methods taking account of sampling strategy. (e) Describe any sensitivity analyses.	4		
Data access and cleaning methods		N/A	4	RECORD 12.1: Authors should describe the extent to which the investigators had access to the database population used to create the study population. RECORD 12.2: Authors should provide information on the data cleaning methods used in the study. 12.3: State whether the study included person-level, institutional-level, or other data linkage across two or more databases. The methods of linkage and methods of linkage quality evaluation should be provided.	
Results					
Participants	13	(a) Report the numbers of individuals at each stage of the study (e.g., numbers potentially eligible, examined for eligibility, confirmed eligible, included in the study, completing follow-up, and analysed). (b) Give reasons for nonparticipation at each stage. (c) Consider use of a flow diagram.	4	RECORD 13.1: Describe in detail the selection of the persons included in the study (i.e., study population selection), including filtering based on data quality, data availability, and linkage. The selection of included persons can be described in the text and/or by means of the study flow diagram.	Figure 1 The participant flow chart
Descriptive data	14	(a) Give characteristics of study participants (e.g., demographic, clinical, and social) and information on exposures and potential confounders. (b) Indicate the number of participants with missing data for each variable of interest. (c) Cohort study: summarise follow-up time (e.g., average and total amount).	4		
Outcome data	15	Cohort study: Report numbers of outcome events or summary measures over time. Case-control study: Report numbers in each exposure category or summary measures of exposure. Cross-sectional study: Report numbers of outcome events or summary measures.	5-12		
Main results	16	(a) Give unadjusted estimates and, if applicable, confounder-adjusted estimates and their precision (e.g., 95% confidence interval). Make clear which confounders were adjusted for and why they were included. (b) Report category boundaries when continuous variables were categorized. (c) If relevant, consider translating estimates of relative risk into absolute risk for a meaningful time period.	5-12		
Other analyses	17	Report other analyses done—e.g., analyses of subgroups and interactions and sensitivity analyses.	8		
Discussion					

For peer review only

Key results	18	Summarise key results with reference to study objectives.	13		
Limitations	19	Discuss limitations of the study, taking into account sources of potential bias or imprecision. Discuss both direction and magnitude of any potential bias.	14	RECORD 19.1: Discuss the implications of using data that were not created or collected to answer the specific research question(s). Include discussion of misclassification bias, unmeasured confounding, missing data, and changing eligibility over time, as they pertain to the study being reported.	
interpretation	20	Give a cautious overall interpretation of results considering objectives, limitations, multiplicity of analyses, results from similar studies, and other relevant evidence.	14		
Generalisability	21	Discuss the generalisability (extemal validity) of the study results.	13-14		
Other Information					
Funding	22	Give the source of funding and the role of the funders for the present study and, if applicable, for the original study on which the present article is based.	14		
Accessibility of protocol, raw data, and programming code		N/A	14-15	RECORD 22.1: Authors should provide information on how to access any supplemental information such as the study protocol, raw data, or programming code.	
N/A, not applicable					

For peer review only
